# *Phytophthora theobromicola* sp. nov.: A New Species Causing Black Pod Disease on Cacao in Brazil

**DOI:** 10.3389/fmicb.2021.537399

**Published:** 2021-03-15

**Authors:** Jennifer Decloquement, Roberto Ramos-Sobrinho, Samuel Galvão Elias, Dahyana Santos Britto, Alina Sandra Puig, Ailton Reis, Rildo Alexandre Fernandes da Silva, Jaime Honorato-Júnior, Edna Dora Martins Newman Luz, Danilo Batista Pinho, Jean-Philippe Marelli

**Affiliations:** ^1^Departamento de Fitopatologia, Universidade de Brasília, Brasília, Brazil; ^2^Biophyto Plant Health LTDA ME, Maceió, Brazil; ^3^Mars Center for Cocoa Science, Barro Preto, Brazil; ^4^USDA-ARS/Subtropical Horticultural Research Station, Miami, FL, United States; ^5^Embrapa, Centro Nacional de Pesquisa de Hortaliças, Brasília, Brazil; ^6^Centro Multidisciplinar do Campus de Barra, Universidade Federal do Oeste da Bahia, Barra, Brazil; ^7^Centro de Pesquisas do Cacau, Comissão Executiva do Plano da Lavoura Cacaueira, Ilhéus, Brazil; ^8^Mars Plant Sciences Laboratory, Davis, CA, United States

**Keywords:** oomycetes, new taxon, Peronosporales, plant pathology, tropical fungi

## Abstract

Black pod disease, caused by *Phytophthora* species, is among the main limiting factors of cacao (*Theobroma cacao* L.) production. High incidence levels of black pod disease have been reported in Brazil, being induced by *Phytophthora capsici*, *Phytophthora citrophthora*, *Phytophthora heveae*, and *Phytophthora palmivora*. To assess the diversity of *Phytophthora* species affecting cacao in Brazil, 40 new isolates were obtained from cacao pods exhibiting symptoms of black pod disease collected in different smallholder farms in 2017. Further, ten cacao-infecting isolates morphologically identified as *P. citrophthora* and *P. palmivora* were molecularly characterized. The genomic regions beta-tubulin, elongation factor 1 alpha, heat shock protein 90, and internal transcribed spacer, and the mitochondrially encoded cytochrome *c* oxidase I and II genes were PCR-amplified and Sanger-sequenced from the cacao-infecting *Phytophthora* isolates. The morphological characterization and evaluation of the mycelial growth rates for the *Phytophthora* isolates were performed *in vitro*. Based on the molecular analysis and morphological comparisons, 19 isolates were identified as *P. palmivora* (clade 4). Interestingly, 31 isolates grouped together in the phylogenetic tree and were placed apart from previously known species in *Phytophthora* clade 2. Therefore, these isolates are considered as a new species herein referred to as *Phytophthora theobromicola* sp. nov., which produced papillate, semipapillate, and persistent sporangia on simple sporangiophores. The *P. palmivora* isolates were identified as A1 mating type by pairing each isolate with known A1 and A2 tester strains of *P. capsici*, but no oogonia/antheridia were observed when *P. theobromicola* was paired with the different tester strains. The *P. theobromicola* and *P. citrophthora* isolates showed higher mycelial growth rates, when compared to *P. palmivora*, on different media at 10, 15, and 20°C, but similar values were observed when grown on clarified CA media at 25 and 30°C. The pathogenicity tests carried out on pods of four cacao clones (CCN51, PS1319, Cepec2004, and CP49) showed significant variability among the isolates of both *Phytophthora* species, with *P. theobromicola* inducing higher rates of necrotic lesion expansion, when compared to *P. palmivora*. Here, two *Phytophthora* species were found associated with black pod disease in the state of Bahia, Brazil, and the previously undescribed *P. theobromicola* seems to be prevalent in field conditions. This is the first report of *P. theobromicola* on *T. cacao*. Also, these findings are crucial to improve the disease control strategies, and for the development of cacao materials genetically resistant to *Phytophthora*.

## Introduction

Cacao (*Theobroma cacao* L.) is a perennial plant native to the Amazon region belonging to the family Malvaceae, and it is of great socioeconomic importance worldwide (Chinenye et al., 2010; Zhang and Motilal, 2016). Brazil is among the world’s largest producers of cocoa beans (Lopes et al., 2011; Agrianual, 2017), but diseases such as witches’ broom, black pod, and anthracnose, caused by *Moniliophthora perniciosa*, *Phytophthora*, and *Colletotrichum* species, respectively (Rocha and Ram, 1971; Pereira et al., 1980; Ploetz, 2016), are still responsible for significant yield losses. In 2013, the average productivity of dried cocoa beans in Brazil was 0.37 t/ha, while in Ivory Coast, the world’s largest producer, it was 0.58 t/ha (Agrianual, 2017; Faostat, 2017). In Brazil, cacao is an important economic source for many states such as Bahia, Pará, Espírito Santo, Amazonas, and Mato Grosso. Currently, the southern region of the Bahia state is the main cocoa-producing area, at approximately 165,000 tons in 2016, followed by the North region of the country with 104,000 tons. Although the higher production values, a lower productivity (0.34 t/ha) was observed in Bahia, when compared to North Brazil (0.72 t/ha), and it is believed that the low productivity of cocoa beans in that state is mainly due to the high incidence of diseases such as black pod (Luz et al., 2001; Agrianual, 2017).

*Phytophthora* is historically associated with the negative impacts induced by widespread agriculturally and ecologically important plant diseases (Brasier, 1992; Brasier and Hansen, 1992; Erwin and Ribeiro, 1996; Brasier et al., 2003; Yang et al., 2017; Jung et al., 2018; Anandaraj et al., 2020). *Phytophthora* species colonize all parts of the cocoa tree, causing black pod rot, stem canker, and leaf blight at nurseries (Firman and Vernon, 1970; Appiah et al., 2004; Marelli et al., 2019). The black pod disease, caused by *Phytophthora* species, is the most important and widely distributed disease that affects cacao pods, being responsible for about 50% of the losses induced by pests and diseases on this crop (Marelli et al., 2019). The symptoms and progression of the black pod disease depend on the cacao genotype and the *Phytophthora* species involved (Guest, 2007; Puig et al., 2018). In addition, both are influenced by climatic factors such as relative humidity, temperature, and rainfall (Oliveira and Luz, 2005; Puig et al., 2018).

Seven *Phytophthora* species have been reported causing black pod disease on cacao, so far, with *Phytophthora megakarya*, *Phytophthora palmivora*, *Phytophthora capsici*, and *Phytophthora citrophthora* being considered the most important species (Ali et al., 2016; Marelli et al., 2019). In Brazil, *P. capsici*, *P. citrophthora*, *Phytophthora heveae*, and *P. palmivora* have been associated with high incidence levels of black pod disease, but these *Phytophthora* species were identified using only morphological comparisons (Luz et al., 2018; Lessa et al., 2020). It has been shown that molecular approaches, associated with morphological data, are required to resolve plant pathogen species complex, and they have revealed previously uncharacterized species affecting different crops (Cooke et al., 2000; Blair et al., 2008; Bezuidenhout et al., 2010; Martin et al., 2014; Jung et al., 2017; Yang et al., 2017; Ruano-Rosa et al., 2018; Yang and Hong, 2018; Alves et al., 2019). Interestingly, several studies have shown that the genetic resistance to black pod rot in cacao is associated with different QTLs depending on the species of *Phytophthora* (Risterucci et al., 2003; Barreto et al., 2018). Therefore, it is particularly important for cacao breeding studies to correctly identify the *Phytophthora* species that are used during phenotype screenings. In this direction, a PCR-based method was developed to distinguish between *Phytophthora megakarya* and *P. palmivora* (Ali et al., 2016) to help West African breeders using the correct species in their programs. Also, the precise identification of the *Phytophthora* species in each growing area, combined with the phytosanitary measures that prevents circulation of contaminated seedlings and pods, can minimize the damages caused by black pod disease of cacao.

Here, *Phytophthora* isolates causing black pod disease on cacao in Bahia, Brazil, were morphologically and molecularly characterized. Two species, *P. palmivora* and *Phytophthora theobromicola* sp. nov., were identified, and the previously undescribed *P. theobromicola* seems to be prevalent in the cacao-producing areas in the Bahia state. Also, *P. theobromicola* induced higher rates of necrotic lesion expansion on pods of different cacao genetic materials.

## Materials and Methods

### Plant Material

Cacao pods showing typical symptoms of black pod disease were collected between June and December 2017 in the municipalities of Porto Seguro (cacao cultivated under full-sun), Eunápolis (cacao intercropped with coconut), Barro Preto (agroforestry system known as cabruca^1^), and Igrapiúna (cacao intercropped with rubber tree), Bahia, Brazil. Ten pods were collected randomly at distant points in each location (*n* = 40), individually placed into paper bags, shipped to the Mars Center for Cocoa Science (MCCS), Barro Preto, Bahia, Brazil, and stored at room temperature (25 ± 2°C).

### *Phytophthora* Isolation

The symptomatic pods were washed in tap water and liquid soap, and dried using paper towels. Then, pod fragments (5 × 5 mm) were removed from the transition region between diseased and healthy tissues, and superficially disinfested in 70% ethanol for 30 s, followed by 60 s in 1.5% sodium hypochlorite. The plant material was washed in sterile distilled water and deposited on carrot-agar medium amended with the antibiotics pimaricin, ampicillin, and rifampicin, and the fungicide PCNB (CA-PARP), at 10, 250, 10, and 100 mg/L, respectively (Luz et al., 2018). The mycelial growths from the pod husk fragments were individually transferred to new Petri dishes containing carrot-agar (CA) medium, and kept at 25 ± 2°C (Jung et al., 2003; Alfenas et al., 2016; Ruano-Rosa et al., 2018). To ensure the genetic purity of the isolates, hyphal tip cultures were obtained.

Eight cacao-infecting isolates previously identified, based on morphological data, as *P. palmivora* (*n* = 3) and *P. citrophthora* (*n* = 5) (Luz et al., 2018) were included in the molecular analyses reported here. Further, two isolates causing black pod disease on cacao (isolates P0449 and P1839) in Brazil, and two isolates infecting citrus (isolates P0479 and P1159), and previously deposited as *P. citrophthora* (ATTC Mycology Collection) were included in our data set (Table 1).

**TABLE 1:** GenBank accession numbers of DNA sequences of *Phytophthora* isolates obtained in this study and included in the multigenic analysis.

Species	Isolate	*BTUB*	*ITS*	*COXI*	*COXII*	*HSP90*	*EF1*α
*P. citrophthora*	P1159*	MW597329	MW597338	MW597356	MW597365	MW597375	MW597383
*P. palmivora*	CCUB 906	MT074250	MT074270	MW597353	MT074278	MT074294	MT074286
*P. palmivora*	CCUB 920	MT074249	MT074269	MW597354	MT074277	MT074293	MT074285
*P. palmivora*	CCUB 1102	MT074247	MT074267	MW597348	MT074275	MT074291	MT074283
*P. palmivora*	CCUB 1158	MT074248	MT074268	MW597350	MT074276	MT074292	MT074284
*P. palmivora*	CCUB 2700^†^	MW597322	MW597331	MW597340	MW597358	MW597367	MW597376
*P. palmivora*	CCUB 2711^†^	MW597324	MW597333	MW597345	MW597360	MW597369	MW597378
*P. palmivora*	CCUB 2712^†^	MW597327	MW597336	MW597347	MW597363	MW597372	MW597381
*P. theobromicola*	P0449*	MW597330	MW597339	MW597355	MW597366	MW597374	MW597384
*P. theobromicola*	P1839*	MT036260	MT036256	MW597357	MT036257	MT036259	MT036258
*P. theobromicola*	CCUB 1091	MT074223	MT074263	MW597344	MT074271	MT074287	MT074279
*P. theobromicola*	CCUB 1151	MT074226	MT074266	MW597349	MT074274	MT074290	MT074282
*P. theobromicola*	CCUB 1205	MT074224	MT074264	MW597351	MT074272	MT074288	MT074280
*P. theobromicola*	CCUB 1285	MT074225	MT074265	MW597352	MT074273	MT074289	MT074281
*P. theobromicola*	CCUB 2706**	MW597328	MW597337	MW597341	MW597364	MW597373	MW597382
*P. theobromicola*	CCUB 2707**	MW597323	MW597332	MW597342	MW597359	MW597368	MW597377
*P. theobromicola*	CCUB 2708**	MW597325	MW597334	MW597346	MW597361	MW597370	MW597379
*P. theobromicola*	CCUB 2710**	MW597326	MW597335	MW597343	MW597362	MW597371	MW597380

*ATCC Mycology Collection; ^†^Isolates previously identified as Phytophthora palmivora (Luz et al., 2018); ** Isolates previously identified as Phytophthora citrophthora (Luz et al., 2018).

### Total DNA Extraction

The *Phytophthora* isolates were grown on Petri dishes containing CA medium, covered with sterile cellophane paper, at 25 ± 2°C for 3–5 days (Scanu et al., 2014). The mycelial growth was collected using a sterile toothpick and deposited in 1.5 mL microtubes containing 50 μL of Tris-EDTA (TE) buffer, four metal beads (2.8 mm), and 600 μL of Nuclei Lysis Solution (Promega^®^). The total DNA extraction was performed using the Wizard Genomic DNA Purification Kit (Promega^®^) according to the manufacturer’s instructions. The total DNA preparations were analyzed on 1% agarose gel electrophoresis, stained with GelRed (Biotium^®^), and visualized under UV light. The DNA samples were stored at −20°C for later use.

### Amplification and Sequencing

The β-tubulin (*BTUB*) partial sequences were amplified, by Polymerase Chain Reaction (PCR), with the primer set BTub_F1/TUBUR1 (Kroon et al., 2004; Blair et al., 2008), and used as the primary barcode for identification of *Phytophthora* species due to the high PCR success rate and easy alignment of the nucleotide sequences. To attribute definitive species demarcation for the *Phytophthora* isolates, nucleotide sequences of the nuclear genes heat shock protein 90 (*HSP90*) and elongation factor 1 alpha (*EF1*α), the internal transcribed spacers of the nuclear ribosomal DNA (*ITS*), and two mitochondrial genes, cytochrome *c* oxidase subunit I (*COXI*) and cytochrome c oxidase subunit II (*COXII*) were obtained from representative isolates preliminarily identified based on *BTUB* sequence data (Supplementary Table 1). The *HSP90*, *EF1*α, *ITS*, *COXI*, and *COXII* regions were amplified using the primer pairs HSP90_F1/HSP90_R2 (Blair et al., 2008), EF1A_for/EF1A_rev (Blair et al., 2008), DC6/LR0 (Bonants et al., 1997; Choi et al., 2006), OomCoxI-Levup/Fm85mod (Robideau et al., 2011), and COX2-F/COX2-R (Hudspeth et al., 2000), respectively. The PCR amplifications were performed in a final volume of 12.5 μL: 6.25 μL of MyTaq MasterMix 2x (Bioline, EUA), 0.3 μL (10 pmol/μL) of each primer, 4.25 μL of nuclease-free water, and 1 μl of template DNA (25 ng/μl). The cycling conditions were: initial denaturation at 95°C for 1.5 min, followed by 35 cycles at 95°C for 20 s, 60°C (*BTUB*) for 45 s, and 72°C for 1 min, and a final extension at 72°C for 5 min. Different annealing temperatures were used according to the genomic region to be amplified: 62°C (*HSP90*), 60°C (*EF1*α*)*, 50°C (*COXI* and *COXII*), and 58°C (*ITS*). Also, distinct extension times were used: *COXI, COXII*, and *ITS* (45 s), *EF1*α (1 min), and *HSP90* (1.5 min). The PCR products were purified and bidirectionally Sanger sequenced at Macrogen Inc. (Seoul, South Korea). The new sequences were assembled and manually edited using Geneious v.8.1.9^2^.

### Phylogenetic Analyses

To determine the *Phytophthora* species with which the new isolates shared the highest nucleotide identity, the partial nucleotide sequences and the BLASTn algorithm (Altschul et al., 1990) were used to search the NCBI-GenBank non-redundant nucleotide database. A Bayesian phylogenetic tree was initially reconstructed using the *BTUB* sequences from the 50 isolates characterized here, and representative isolates of the *Phytophthora* species belonging to clade 2 (Supplementary Tables 1, 2; Blair et al., 2008; Bezuidenhout et al., 2010; Martin et al., 2014; Yang et al., 2017; Ruano-Rosa et al., 2018; Yang and Hong, 2018; Alves et al., 2019; Abad, 2020). The reference specimen P0633 of *P. palmivora* (clade 4) was used as outgroup. Also, phylogenetic trees were individually inferred from each genomic region analyzed here. Multiple sequence alignments were obtained with MAFFT v7 (Katoh and Standley, 2013). Finally, Bayesian Inference (BI) and Maximum Likelihood (ML) phylogenetic trees were reconstructed using the concatenate data (*COXI*, *COXII*, *BTUB*, *ITS*, *EF1*α, and *HSP90*). For BI, the best nucleotide substitution models were determined, for each partition, with MrModeltest (Posada and Buckley, 2004). The CIPRES web portal (Miller et al., 2010) was used to run MrBayes v3.2.1 (Ronquist and Huelsenbeck, 2003). The Markov Chain Monte Carlo (MCMC) analysis was run with a total of 10 million generations, sampling every 1,000 generations. The convergence of the log likelihoods was confirmed using TRACER v1.7.1 (Rambaut and Drummond, 2018). The first 25% of the sampled trees were discarded as burn-in, with the posterior probability (PP) values calculated with the remaining trees (Rannala and Yang, 1996). The ML tree was reconstructed using RAxML v.8 (Stamatakis, 2014) accessed through the CIPRES web portal (Miller et al., 2010), assuming a general time reversible (GTR) nucleotide substitution model with a gamma (G) rate of heterogeneity, and 1,000 bootstrap replicates. The phylogenetic trees were visualized and edited in FigTree v1.4 (Rambaut, 2018) and Inkscape^3^.

### Morphological and Cultural Characterization

The morphological characterization of eight-representative cacao-infecting *Phytophthora* isolates was performed using hyphal tip cultures grown on CA medium during 7 days at 20 ± 1°C (Jung et al., 2003; Abad and Coffey, 2010; Ruano-Rosa et al., 2018). The sporangiophore morphology was observed by submerging nine 10 mm^2^ disks, obtained from the growing edge of a 7-day-old V8A colony in a 90 mm diameter Petri dish, in non-sterile soil extract (50 g of filtered forest soil in 1,000 mL of distilled water, filtered after 24 h) (Erwin and Ribeiro, 1996). The Petri dishes were incubated at 20 °C in natural daylight and morphological characteristics were observed after 24 h. The formation of clamidospores, and their features, was examined from 30-day-old colonies grown on water-agar (WA) at 20°C in the dark. The mating types were determined by individually pairing the 50 isolates with A1 and A2 tester strains of *P. capsici* and *P. nicotianae* on CA media, kept in the dark for 15 days at 25 ± 1°C. The isolate CCUB1089, previously identified as *P. palmivora* A1 mating type (Decloquement et al., 2019), was also used in the pairing tests. Further, each self-sterile isolate was paired with all isolates reported in this study, and with A1 and A2 mating types of *P. capsici*, *P. nicotianae*, *P. palmivora*, and *P. citrophthora* on Petri dishes containing clarified V8 juice agar, 2% malt-extract-agar (MEA), potato-dextrose-agar (PDA), WA or CA media, with or without β-sitosterol. The cultures were kept at 20 or 25°C in the dark for 30 days. The microscopical characteristics were analyzed by mounting asexual structures in clear lactoglycerol, and 50 measurements for each morphological parameter were carried out at a magnification of × 1,000 using a Leica DM2500 light microscope equipped with a Leica DFC 490 digital camera, coupled to a computer containing the Leica Qwin-Plus software. The dried culture holotypes were deposited at the Universidade de Brasília (UB) Herbarium, while ex-type cultures were stored on CA disks and grains of rye immersed in sterile water in plastic vials kept at 10°C in the Coleção de Culturas da Universidade de Brasília (CCUB; Brasília, Brazil) (Table 1).

The colony growth rates were determined, for representative isolates of *P. theobromicola* sp. nov., *P. citrophthora*, and *P. palmivora*, on clarified 20% V8 juice-agar (V8A), 2% MEA, PDA, and CA during 7 days at 20 ± 1°C (Erwin and Ribeiro, 1996; Scanu et al., 2014; Ruano-Rosa et al., 2018). The radial colony growth was recorded daily along two perpendicular lines that intersected the center of the initial inoculum (mycelial plugs of 5 mm in diameter). To determine the cardinal temperatures, the isolates were initially grown on CA medium for 5 days at 25 ± 1°C. Then, mycelial plugs (5 mm in diameter) were transferred to new Petri dishes (90 mm) containing CA medium, and kept at 5, 10, 15, 20, 25, 30, 35, or 40 ± 1°C for 7 days in the dark. The Petri dishes showing no mycelial growth (5, 35, and 40°C) were returned to 25°C to determine the viability of the isolates. The measurements of colony diameters were performed as described above. The tests were independently repeated twice, and treatments were comprised by four replicates, following the inter-randomly design, with each Petri dish being considered as an experimental unit.

### Pathogenicity Tests

The representative isolates of *P. theobromicola* sp. nov. and *P. palmivora*, identified by multilocus and morphological/cultural comparisons, and the type isolate of *P. citrophthora* (Table 1) were used in pathogenicity tests on pods of different cacao genotypes (CCN51, Cepec2004, CP49, and PS1319). Asymptomatic pods (4–5-month-old) were washed in tap water and liquid soap, and dried using paper towels. The cacao pods were wounded, using a sterile needle, at two distinct points located approximately 5 cm from each peduncle and tip. Then, a mycelial plug (5 mm), from a 5-day-old culture grown on CA at 25 ± 1°C, was deposited on each wounded area. The treatments were comprised by five replicates for each *Phytophthora* isolate and cacao clone. CA plugs (with no mycelial growth) were used on mock-inoculated treatments. The pods were kept in a humid chamber at 20°C ± 1°C for 7 days post inoculation, and the necrotic lesion diameter was evaluated daily, for 5 days, after 48 h of incubation period. The pathogenicity tests were independently repeated twice, and each pod was considered as an experimental unit.

### Statistical Analysis

The data from the pathogenicity assay were processed with primary data validation performed with a logistic regression (logit link function) to identify whether the inoculum position was a determining factor for necrotic lesion expansion (event 1) or not (event 0). This strategy was adopted to verify if putative differences in the ripeness stages between the inoculation points could affect the disease progress. The linear equation used in the logistic fit included a full model containing elapsed time, *Phytophthora* isolates, cacao clones, inoculum position and all interactions among these variables.

Given the multifactorial nature of the experimental design growth kinetics, data from the cultural characterization were fitted through linear mixed-effects models. Two competing models were tested: the null model using the elapsed time, culture media and *Phytophthora* species as fixed effects, and the isolate level as a random effect (*Average mycelial growth* = *time: Phytophthora species: cult. media*) + (1 *| Phytophthora isolate*); and the alternative model, which differed from the null model because it included a *Phytophthora* isolate as a fixed effect instead of an effect at the species level. Both models were compared to verify whether the inclusion of isolate level variation (alternative model) produced a less erroneous model. Pairwise comparisons were performed with adjusted Tukey’s HSD multiplicity test. The statistical analyses were performed in R v 3.5 (Pinheiro et al., 2020) and data is publicly available on GitHub^4^.

## Results

### *Phytophthora* Isolates Infecting Cacao Pods

A specimen was obtained from each cacao pod exhibiting typical symptoms of black pod disease such as brown and circular lesions covered by a white mycelial growth. The forty novel *Phytophthora* isolates were initially identified based on *BTUB* sequences (Supplementary Table 1), and eight representative isolates were selected for morphological and *in vitro* mycelial growth characterization, and evaluation of pathogenicity on pods of different cacao genotypes. Additionally, three and five isolates previously identified, based on morphological data, as *P. palmivora* and *P. citrophthora*, respectively (Luz et al., 2018), and two isolates deposited in the ATCC Mycology Collection were added to our data set. Therefore, a total of 50 cacao-infecting *Phytophthora* isolates were molecularly characterized in the present study.

### Phylogenetic Relationship

**FIGURE 1:**
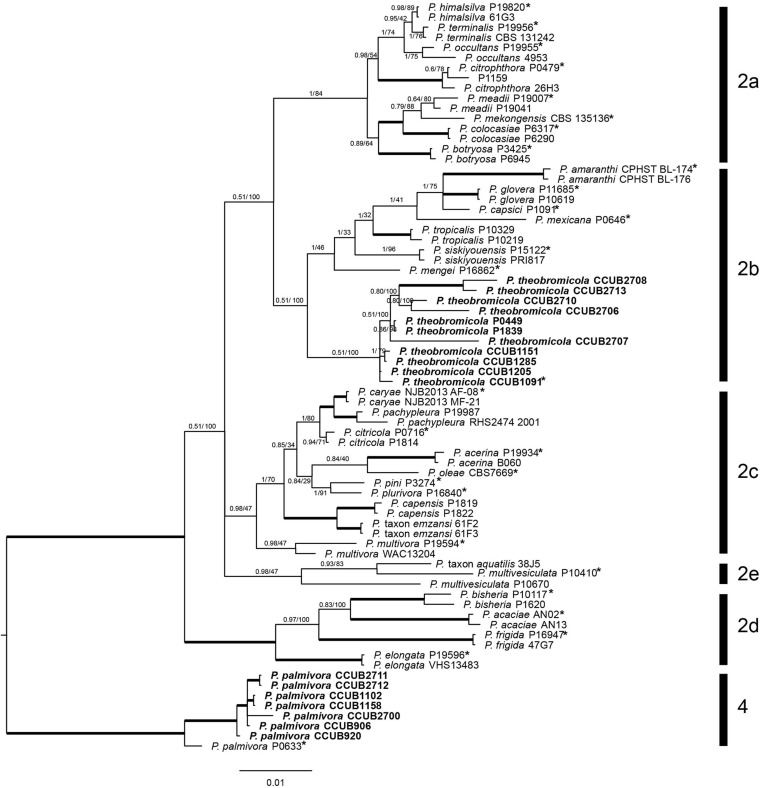
Bayesian phylogenetic tree based on concatenate sequences (*BTUB*, *COXI*, *COXII*, *EF1*α, *ITS*, and *HSP90*) of *Phytophthora* clade 2. Bayesian posterior probabilities and Maximum Likelihood bootstrap support values are indicated at the nodes, and the scale bar represents the number of expected changes per site. Thickened lines indicate a posterior probability = 1.00. Ex-type isolates or reference specimens are indicated with an asterisk, and the isolates reported here are highlighted in bold. The reference specimen P0633 of *Phytophthora palmivora* was used as outgroup.

To perform an initial species identification of the cacao-infecting *Phytophthora* isolates (*n* = 50), *BTUB* partial nucleotide sequences were obtained from each isolate. The fifty new *BTUB* sequences were 1,100 bp in length, and the aligned data set was comprised by 1,106 characters, from which 804 and 206 were classified as conserved and parsimony informative sites, respectively. The BI tree was reconstructed using the GTR + I + G nucleotide substitution model. The *BTUB* tree showed that the *Phytophthora* isolates were grouped into two distinct clades (the nucleotide matrices and phylogenetic tree are available in TreeBASE; study number S25541). Then, 18 isolates from both *BTUB* clades, representing each sampling location in the Bahia state, Brazil, and the *Phytophthora* isolates previously reported, were selected for amplification and sequencing of the *COXI, COXII, EF1*α, *ITS*, and *HSP90* regions, which were 600, 560, 800, 1,000, and 900 bp in length, respectively. The new *COXI, COXII, EF1*α, *ITS*, *BTUB*, and *HSP90* partial sequences were deposited in GenBank (Table 1). To correctly determine the *Phytophthora* species, a multilocus approach was adopted using the *ITS*, *BTUB*, *EF1*α, *COXI*, *HSP90*, and *COXII* sequences. A total of 74 taxa [18 isolates molecularly characterized here, and 56 isolates from GenBank (Supplementary Table 2)] were included in the BI and ML phylogenetic analyses. The *ITS*, *BTUB*, *EF1*α, *COXI*, *HSP90*, and *COXII* individually aligned data sets were 418, 779, 829, 492, 885, and 517 bp in length, respectively (single gene trees are available in TreeBASE; study number S25541). The concatenate alignment was comprised by 3,923 characters, with 3,166 and 745 conserved and variable sites, respectively. Also, 605 sites were determined as phylogenetically informative. The Bayesian phylogenetic tree was reconstructed considering the best nucleotide substitution model for each partition in the concatenate data, HKY + I + G (*ITS*) and GTR + I + G (*BTUB*, *COXII*, *EF1*α, *COXI*, and *HSP90*). The *Phytophthora* isolates reported here were grouped in two different phylogenetic clades (*Phytophthora* clades 2 and 4). Seven isolates clustered with other *P. palmivora* (clade 4), while eleven isolates grouped together in the phylogenetic tree and were placed apart from previously known species in *Phytophthora* clade 2b. Together with the morphological data (see below), these isolates are considered as a new species herein referred to as *Phytophthora theobromicola* sp. nov. Interestingly, the isolates infecting cacao in Brazil, and previously identified as *P. citrophthora* (Table 1; Luz et al., 2018), were placed in the same clade with *P. theobromicola*. Also, the isolates P0449 and P1839 clustered with *P. theobromicola* (Figure 1). The ML and Bayesian phylogenetic trees showed similar topologies (Figure 1).

### Taxonomy

**FIGURE 2:**
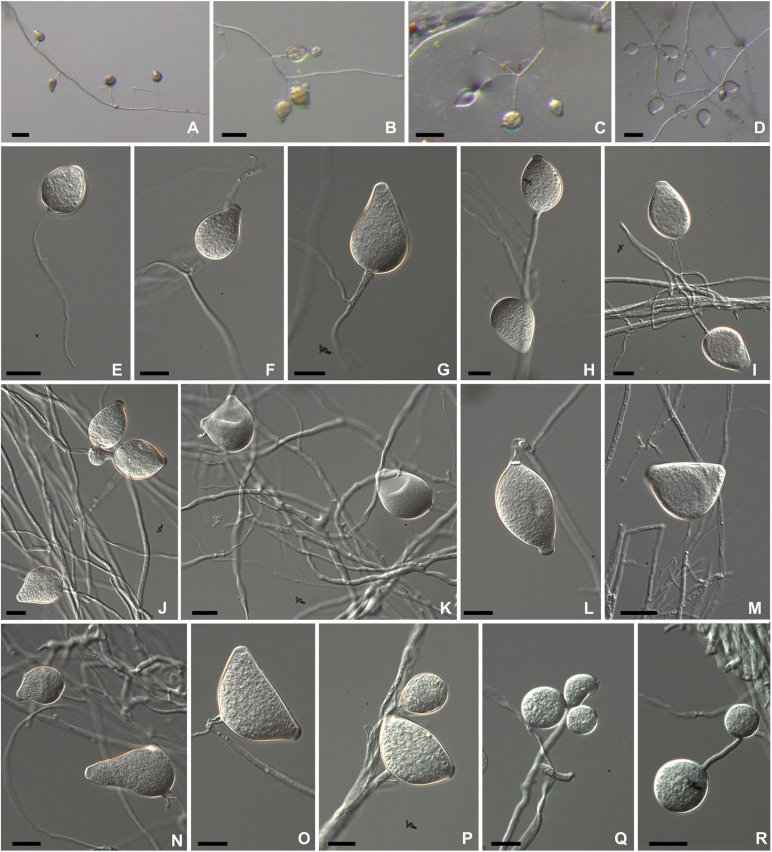
Morphological structures of *Phytophthora theobromicola* isolate CCUB1091 formed on V8-juice-agar flooded with soil extract **(A–D)** and carrot-agar **(E–P)**. Sporangia borne on simple sporangiophores produced in non-sterile soil extract **(A–D)**. Subglobose to globose semipapillate sporangium **(E)**. Obpyriform papillate sporangia **(F,G,I,J,L,N)**. Ovoid semipapillate sporangia **(H,K)**. Turbinate bipapillate sporangia **(M,O,P)**. Globose chlamydospores produced on water-agar **(Q–R)**. Bars: panels **(A–D)** = 500 μm; panels **(E–R)** = 20 μm.

*Phytophthora theobromicola* Pinho, Ramos-Sobrinho and Marelli sp. nov. Figure 2.

MycoBank: MB 833783

*Typification*: Brazil, Bahia: Eunápolis, on pods of *Theobroma cacao*, July 2017, coll. R. Ramos-Sobrinho (holotype UB23904). Ex-type living culture CCUB1091.

GenBank: *ITS* = MT074263; *COXI* = MW597344; *BTUB* = MT074223; *HSP90* = MT074287; *EF1*α = MT074279; *COXII* = MT074271.

*Etymology*: Name refers to only known host so far, *Theobroma cacao*.

**FIGURE 3:**
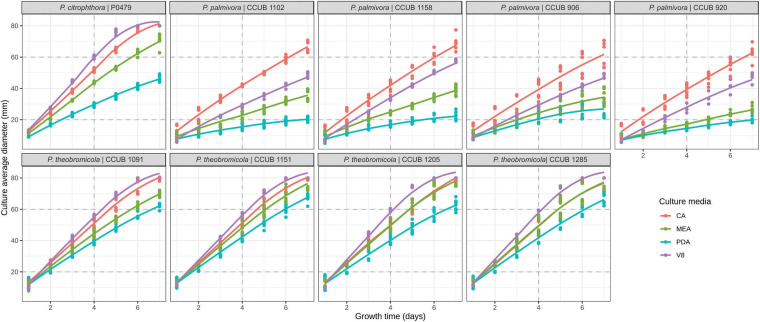
Growth rates of *Phytophthora* species on different culture media. Colony diameter measurements are represented by dots, and the predicted profiles through a linear mixed-effects model are indicated by continuous line. Culture media are highlighted in blue (potato-dextrose-agar; PDA), red (carrot-agar; CA), green (2% malt-extract-agar; MEA), and purple (V8-juice-agar; V8A).

Colonies with chrysanthemum pattern on PDA, MEA, and V8A, and radiate on CA at 20°C (Figure 3). Hyphal swellings not observed. Sporangia persistent, monopapillate (96%; Figures 2F,G,I,J,L,N), bipapillate (4%; Figures 2M,O,P), or semipapillate (1%; Figures 2E,H,K). Variable sporangial shapes, obpyriform, and ovoid are often observed (89%; Figures 2F–L,N), while subglobose to globose (7%; Figure 2E) and turbinate (4%; Figures 2M,O,P) are unusual shapes (Figures 2E,M,O,P), 27–76 μm long (av. 52.3 ± 9.1 μm) and 21.5–45.5 μm wide (av. 35.5 ± 4.3 μm), some with tapered bases, borne in simple sporangiophores (Figures 2A–D). Zoospores discharged through an exit pore 6.0–12 μm wide (av. 8.0 ± 1.5 μm), subglobose or ovoid whilst motile. Chlamydospores formed on water agar, globose, subglobose, lateral, terminal, and intercalary (Figures 2Q–R), measuring 19.5–45 μm in diameter (av. 30.4 ± 5.2 μm) (Table 2). From the 31 isolates examined, all of them are sterile on clarified V8 juice agar, MEA, PDA, WA, or CA media, with or without β-sitosterol.

**TABLE 2:** Morphological characters and dimension relations of *Phytophthora theobromicola* compared to reference isolates of *Phytophthora* species from subclade 2b.

*Phytophthora* species	Sporangia (average)			Papilae	Clamidospore	
	Length	Width	L/W			References
*P. theobromicola*	27.0–76 (52.3)	21.5–45.5 (35.5)	1.2–1.7 (1.5)	3.0–8.5 (5.1)	19.5–47.0 (30.4)	This study
*P. mengei*	37.4–95.0 (62.7)	27.2–44.2 (35.2)	(1.8)	6.6–13.5	NO	Hong et al., 2009
*P. siskiyouensis*	46–70 (55)	30–51 (36)	1.1–2.0 (1.5)	ND	NO	Reeser et al., 2007
*P. tropicalis*	40–55	19–27	1.8–2.4	ND	27–33	Aragaki and Uchida, 2001
*P. amaranthi*	40–70 (52.2)	30–50 (38.2)	1.1–1.86 (1.37)	ND	NO	Ann et al., 2016
*P. capsici*	35–105 (60)	21–56 (36)	1.6–2.2 (1.8)	ND	22–52	Erwin and Ribeiro, 1996
*P. glovera*	43–61 (50)	26–36 (32)	(1.56)	7–9 (8)	NO	Abad et al., 2011
*P. mexicana*	46–77 (45.2)	16–33 (23.1)	1.3	ND	29–44	Erwin and Ribeiro, 1996
*P. acaciae*	28–85 (51)	21–50 (31)	1.4–1.9 (1.6)	6–7	15–55 (32)	Alves et al., 2019

*NO, not observed; ND, not determined.*

Additional specimens examined: BRAZIL, Bahia: Igrapiúna, on pods of *Theobroma cacao*, July 2017, coll. J. Honorato Júnior (culture CCUB1205); BRAZIL, Bahia: Porto Seguro, on pods of *Theobroma cacao*, July 2017, coll. D. B. Pinho (culture CCUB1285); BRAZIL, Bahia: Barro Preto, on pods of *Theobroma cacao*, July 2017, coll. J. Decloquement (culture CCUB1151).

Known substrata: pods of *Theobroma cacao*.

Known geographic distribution: Brazil.

**FIGURE 4:**
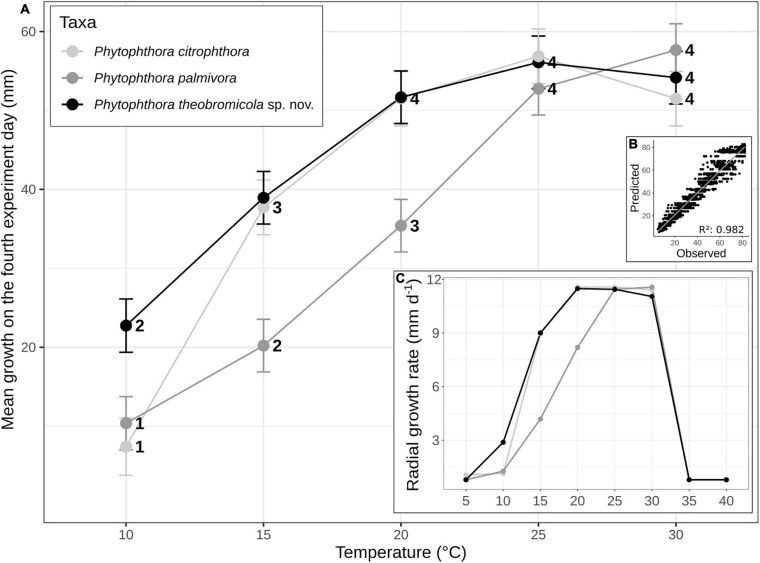
Mean (± SE) of growth rates of *Phytophthora* species on carrot-agar (CA) at different temperature intervals. The statistical significance (95% confidence interval) of paired comparisons, performed between linear mixed-effects model estimates, is indicated by numbers above the horizontal axis **(A)**. Growth rates of *Phytophthora* species vs. predicted rates using the linear mixed-effect model **(B)**. Mean radial growth rates of *Phytophthora citrophthora* (*n* = 1), *Phytophthora palmivora* (*n* = 4), and *Phytophthora theobromicola* (*n* = 4) isolates on CA at eight different temperatures **(C)**.

Notes: *Phytophthora theobromicola* sp. nov. was historically recognized as *P. citrophthora* due to similar mycelial growth in different culture media and temperatures (Figures 3, 4). The proposed new species can be distinguished morphologically from *P. citrophthora* based on persistent sporangia (Figure 2).

### Cultural Characterization

**FIGURE 5:**
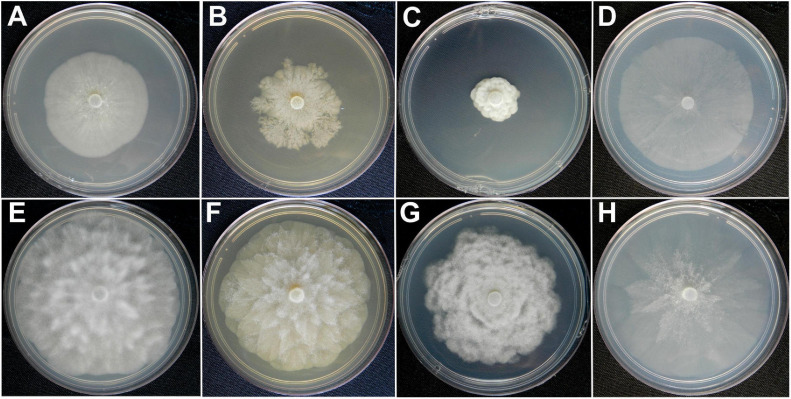
Colony morphologies of *Phytophthora palmivora* (CCUB1102) and *Phytophthora theobromicola* (CCUB1091). Seven-day-old cultures of *P. palmivora* **(A–D)** and *P. theobromicola* **(E–H)** isolates grown on, from left to right, V8-juice-agar (V8A), 2% malt-extract-agar (MEA), potato-dextrose-agar (PDA), and carrot-agar (CA) at 20°C.

The colonies of *P. theobromicola* showed immersed mycelial aspect and chrysanthemum pattern on PDA at 20 ± 1°C, while isolates grown on MEA and V8A have cottony and aerial mycelia with chrysanthemum pattern. Also, the *P. theobromicola* isolates showed cottony and aerial mycelia with radiate pattern on CA at 20 ± 1°C. The *P. palmivora* isolates have colonies with no distinct pattern on V8A and CA, and slow growth on MEA and PDA at 20°C ± 1°C (Figure 5).

The *P. theobromicola* isolates showed higher values of growth rates on CA (10.4 mm/day) and V8A (10.6 mm/day) media, with both being statistically similar, followed by MEA (9.9 mm/day) and PDA (8.4 mm/day). According to the linear mixed-effects model, the mean growth rates of the new taxa were 78, 65, 41, and 21% higher, compared to the *P. palmivora* isolates, on PDA, MEA, V8A, and CA media at 20 ± 1°C, respectively, with all differences being statistically significant (Figure 3).

To determine the cardinal temperatures, the representative isolates from each *P. palmivora* (*n* = 4), *P. theobromicola* (*n* = 4), and *P. citrophthora* (*n* = 1) species were grown on CA at eight different temperatures. The intraspecific variation analysis showed that *P. theobromicola* reached the maximum growth rate of 10.8 mm/day at 20°C, statistically similar to the values observed at 25 and 30°C. The *P. palmivora* isolates showed maximum growth rate of 10.4 mm/day at 25°C, being identical to the values at 30°C. Further, the mycelial growth of *P. theobromicola* and *P. citrophthora* isolates were significantly higher than *P. palmivora* at 20 ± 1°C. But, the *P. theobromicola*, *P. palmivora*, and *P. citrophthora* isolates showed statistically similar rate values at 25 and 30°C (Figure 4). No mycelial growth was observed when colonies of the different species were incubated at 5, 35, or 40°C during 7 days, and not even after the cultures were transferred to 25°C, showing the isolates lost viability when grown at these conditions.

### Pathogenicity Test

**FIGURE 6:**
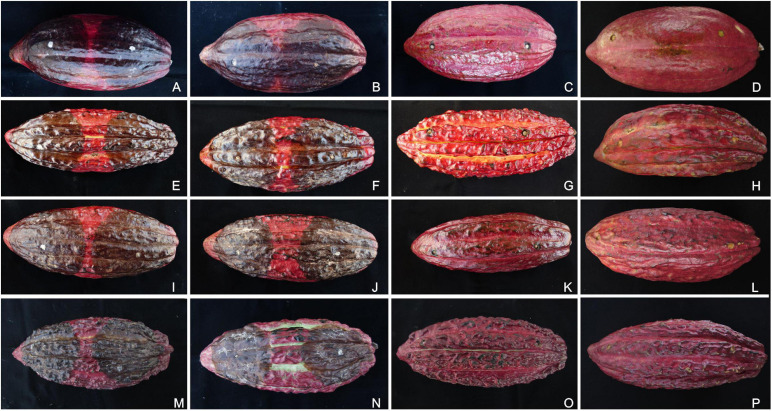
Pathogenicity tests of *Phytophthora* species on artificially inoculated pods of different cacao genotypes. The inoculated pods were kept in a humidity chamber for 7 days at 20°C. The *Phytophthora theobromicola* (CCUB1091; **A,E,I,M**), *Phytophthora palmivora* (CCUB1102; **B,F,J,N**), *Phytophthora citrophthora* (P0479; **C,G,K,O**), and mock treatments **(D,H,L,P)** are shown from left to right, respectively. The cacao clones PS1319 **(A–D)**, Cepec2004 **(E–H)**, CP49 **(I–L)**, and CCN51 **(M–P)** are shown from top to bottom, respectively.

To test the putative effects of the inoculation points on the rates of necrotic lesion development induced by *Phytophthora* isolates, the inoculations were carried out at approximately 5 cm from each polar edge of the cacao pods. The inoculated pods showed symptoms like those observed in the field, and the *Phytophthora* species were successfully reisolated from all symptomatic pods. The mock- and *P. citrophthora* (P0479)-inoculated pods were asymptomatic (Figure 6). The necrotic lesions expanded to a maximum diameter of 134.2 and 165.2 mm when the inoculations were performed near to the pod’s peduncle and tip, respectively. The logistic regression showed no statistical significance in inoculum position effect when evaluated as a single predictor or interacting with all other effects (data available on GitHub; see text footnote 3). Therefore, the different inoculation areas were neglected as a predictor to perform the statistical analysis using the linear mixed-effects model. The alternative model including intraspecific variation of *Phytophthora* aggressiveness produced less erroneous estimates, indicating heterogeneity of both *Phytophthora* species, which was considered in the statistical descriptions reported here.

**FIGURE 7:**
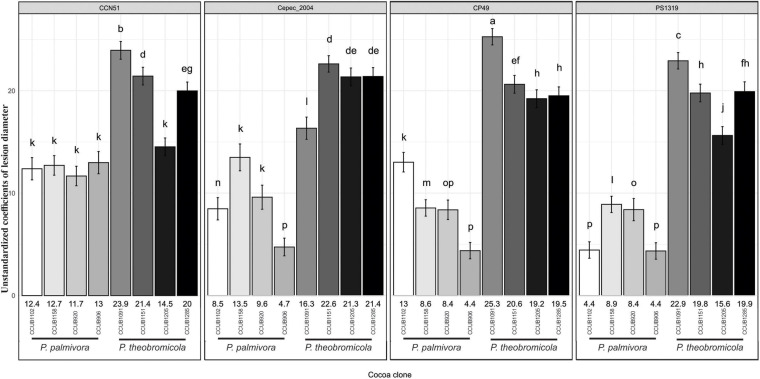
Growth rates of necrotic lesion development induced by *Phytophthora* species on different cacao genotypes. Vertical axis synthesizes mean (± SE) values of unstandardized coefficients generated through linear mixed-effects model. Letters above the error bars indicate statistical significance (95% confidence interval) of global comparisons among coefficients. Numbers at the bottom of each bar represent the mean coefficient values for individual *Phytophthora theobromicola* and *Phytophthora palmivora* isolates.

The aggressiveness of both *Phytophthora* species was highly variable, with the *P. theobromicola* isolates being, in general, more aggressive than *P. palmivora* on pods of the cacao clones CCN51, Cepec2004, CP49, and PS1319 (Figure 7). When considered the intraspecific variation within *P. theobromicola*, the isolates CCUB1151 and CCUB1285 induced statistically homogeneous results among the four cacao genotypes, with necrotic lesions expanding at rates ranging from 19.5 to 22.6 mm/day. The necrotic lesion sizes induced by the CCUB1091 and CCUB1205 isolates were clone dependent. The CCUB1091 isolate induced higher lesion growth rates on the CP49 clone, at 25.3 mm/day, while the lower values were obtained on Cepec2004, at 16.3 mm/day. Contrastingly, the CCUB1205 isolate was more aggressive on Cepec2004, at 21.3 mm/day, with lower values being observed on CCN51, at 14.5 mm/day. The average aggressiveness of the CCUB1205 isolate on Cepec2004 (21.3 mm/day) was statistically identical to the values observed for CCUB1285 and CCUB1151, and higher than the rates observed for CCUB1091 (16.3 mm/day). The PS1319 cacao clone was more susceptible to CCUB1091, at 22.9 mm/day, with the CCUB1205 isolate inducing lower lesion growth rates, at 15.6 mm/day (Figure 7).

The *P. palmivora* isolates induced similar rates of necrotic lesion expansion on pods of the cacao clone CCN51, ranging from 11.7 to 13.0 mm/day. Statistically similar values were observed for the isolates CCUB1158 and CCUB906 when infecting Cepec2004, at 13.5 and 9.6 mm/day, respectively, and lower values were observed for the CCUB920 isolate, at 4.7 mm/day. Finally, the necrotic lesions expanded at rates ranging between 4.4 and 13.0 mm/day on CP49, and 4.4 and 8.9 mm/day on PS1319 pods (Figure 7).

## Discussion

Diseases caused by *Phytophthora* species have been reported in a wide range of economically important crops worldwide and are considered yield-limiting factors (Brasier, 1992; Erwin and Ribeiro, 1996; Spies et al., 2014; Yang et al., 2017; Jung et al., 2018, 2020). Several *Phytophthora* species have been described causing black pod disease on cacao, which poses a great threat to cocoa bean production in the main cultivation areas (Rocha and Ram, 1971; Brasier and Griffin, 1979; Pereira et al., 1980; Erwin and Ribeiro, 1996; Marelli et al., 2019; Armengot et al., 2020). So far, no large-scale study has been carried out using a multilocus approach, associated with morphocultural data, to identify the main *Phytophthora* species infecting this crop in Brazil. In this study, based on morphological and molecular comparisons, at least two *Phytophthora* species are reported associated with black pod disease of cacao in the state of Bahia, Brazil, with one being considered a novel species.

In Brazil, *P. palmivora*, *P. capsici*, *P. citrophthora*, and *P. heveae* have been described associated with black pod disease of cacao (Guest, 2007; Ali et al., 2016; Surujdeo-Maharaj et al., 2016; Luz et al., 2018). Here, the new isolates into the *Phytophthora* clade 2 comprised a monophyletic group separated from previously reported *Phytophthora* species. The new taxon is proposed following the Genealogical Concordance Phylogenetic Species Recognition (GR) criteria (Taylor et al., 2000), and together with morphological and cultural data, is described as a new species referred to as *Phytophthora theobromicola* sp. nov. Interestingly, the cacao-infecting isolates previously recognized as *P. citrophthora* (Luz et al., 2018; ATCC Mycology Collection) were molecularly identified as *P. theobromicola*. The *P. citrophthora* isolates reported infecting cacao in Brazil were initially characterized as belonging to a host-specialized genetic group separated from other *P. citrophthora* affecting a large host range including economically important crops such as *Citrus* spp. (Forster et al., 1990; Oudemans and Coffey, 1991). The similarities between *P. theobromicola* and *P. citrophthora*, especially the mycelial growth rates, probably led to the misidentification of the new species as *P. citrophthora*. Further, the *P. theobromicola* (clade 2b) isolates are phylogenetic distinct from the type isolates of *P. citrophthora* (clade 2a), reinforcing the genetic differences between these two species. *Phytophthora citrophthora* was reported as rarely occurring on cacao in 1981 (Campelo and Luz, 1981), but it is believed that with the expansion of cultivation areas planted with cacao materials resistant to witches’ broom, the population of this pathogen also increased, becoming one of the most important *Phytophthora* species negatively impacting the cacao production in southern Bahia, Brazil (Luz et al., 2018). To date, *P. citrophthora* has not been found in other Brazilian states growing cacao (Luz et al., 2018).

Here, several isolates were identified as *P. palmivora* (clade 4), which are widespread throughout the sampling locations in the Bahia state. The production of cocoa beans in Brazil has been affected by *P. palmivora* since the 1920s (Oliveira and Luz, 2005; Lessa et al., 2020). Southeastern Asia is considered the center of diversity of *P. palmivora*, but it has been found associated with cacao trees worldwide and also being able to infect several wild and cultivated hosts in South America (Wang et al., 2020). *Phytophthora palmivora* has been reported infecting a broad range of hosts in different botanical families (Zentmyer et al., 1973; Erwin and Ribeiro, 1996; Luz et al., 2001; Puig et al., 2021). In Brazil, this pathogen is infecting *Annona squamosa*, *Bactris gasipaes*, *Carica papaya*, *Citrus* sp., *Cocos nucifera*, *Ficus carica*, *Herrania* sp., *Hevea brasiliensis*, *Solanum lycopersicum*, *Piper nigrum*, *T. cacao*, and *T. grandiflorum* (Erwin and Ribeiro, 1996; Luz et al., 2001; Decloquement et al., 2019; Lopes et al., 2019). Recent studies suggest that the host jump of *P. palmivora* from cacao to other plant species was mainly due to the international marketing of cacao seedlings, pods and seeds (Wang et al., 2020). It has been shown that *P. palmivora* is widely distributed in cacao-growing areas around the world, causing annual yield losses of about 20–30% (Ali et al., 2016; Surujdeo-Maharaj et al., 2016; Marelli et al., 2019). Also, *P. palmivora* seems to be prevalent amongst the *Phytophthora* species reported on cacao in field conditions (Erwin and Ribeiro, 1996; Saul Maora et al., 2017; Fernandez Maura et al., 2018; Luz et al., 2018). Here, both *P. palmivora* and *P. theobromicola* were commonly found in the cacao-producing areas located in the state of Bahia. Finally, considering that only the A1 mating type of *P. palmivora* was found in this study, it is possible that a predominant clonal lineage occurs in these growing regions, especially because the presence of both mating types in a same locality would promote hybridization and formation of new isolates (Zentmyer et al., 1973). The black pod disease of cacao induced by *P. palmivora* is known for a long time, and the genetic structure of *P. palmivora* populations showed that one only colonization event was probably responsible for the global pandemic of this pathogen (Wang et al., 2020).

The *P. theobromicola* isolates showed higher values of mean growth rates than *P. palmivora* on different culture media at 20°C. When both species were grown on CA at different temperatures, it was observed that *P. palmivora* isolates reached similar mycelial growth rates, compared to the new taxa, only at 25 and 30°C. These results suggest that *P. theobromicola* may have a wider plasticity when challenged by different substrates and temperatures. Finally, the pathogenicity tests showed that the rates of necrotic lesion expansion are highly dependent on the *Phytophthora* species and isolate, and cacao genotype. However, *P. theobromicola* seems to be more aggressive than *P. palmivora* when inoculated on pods of the cacao clones CCN51, PS1319, Cepec2004, and CP49. Interestingly, the cacao-infecting isolates previously identified as *P. citrophthora* in Brazil, and, here, reclassified as *P. theobromicola*, have also been reported as more aggressive than *P. palmivora* (Luz et al., 2001; Oliveira and Luz, 2005), agreeing with the results observed in the present study. Although further studies are needed to better understand the distribution of *P. theobromicola* in the different cacao-growing regions in Brazil, more restrictive phytosanitary barriers must be adopted to mitigate putative yield losses caused by this pathogen since *P. theobromicola* seems to be more aggressive on different cacao clones and well adapted to a broader temperature range. Also, large-scale studies are required to clarify if *P. theobromicola* emerged from a host jump from a wild plant to cacao and/or by geographical isolation. Finally, the determination of the host range of *P. theobromicola*, and its potential to hybridize with other cacao-infecting *Phytophthora* species, are important epidemiological and evolutionary parameters to be evaluated in future studies.

The guidelines for species demarcation into the genus *Phytophthora* have been changed over the years (Cooke et al., 2000; Forster et al., 2000; Taylor et al., 2000; Martin and Tooley, 2003; Kroon et al., 2004; Schena and Cooke, 2006; Blair et al., 2008; Robideau et al., 2011; Martin et al., 2014; Yang et al., 2017; Abad, 2020). Further, the use of the phylogenetic concept of species has greatly increased the number of species officially recognized, demonstrating that the taxonomical reappraisal of the *Phytophthora* species was crucial (Hong et al., 2009; Jung et al., 2017; Yang et al., 2017; Ruano-Rosa et al., 2018; Alves et al., 2019). Here, the reappraisal of the *Phytophthora* species infecting cacao using a polyphasic approach showed the coexistence of at least two *Phytophthora* species, *P. theobromicola* sp. nov. and *P. palmivora*, widely distributed in the main cultivation areas in Bahia, Brazil. Due to the high number of species, genetic variation, and overlap of morphological characters in *Phytophthora*, the multilocus approach was critical to identify the new *Phytophthora* species proposed here. Also, the precise molecular identification of *P. theobromicola* and *P. palmivora* allowed the establishment of phenotypic parameters separating both species. *Phytophthora theobromicola* has persistent sporangia, while *P. palmivora* has caducous sporangia. *Phytophthora theobromicola* shows higher rates of mycelial growth on CA medium at 20°C, when compared to *P. palmivora*. Finally, the high physiological and pathogenic elasticity of *P. theobromicola* and *P. palmivora* may greatly affect the disease management strategies, including the development of genetically resistant materials and chemical control.

## Data Availability Statement

The datasets generated for this study can be found in Genbank: MT036256-MT036260, MT074223-MT074294, and MW597322-MW597384; Treebase: S25541; GitHub: https://github.com/sgelias/phytophthora-on-cocoa.

## Author Contributions

JD: formal analysis, investigation, data curation, and original draft preparation. RR-S and DP: conceptualization, methodology, investigation, data curation, review and editing, and project administration. SE: formal analysis, data curation, and review and editing. DSB: resources and review and editing. AR and EL: conceptualization and review and editing. RS: formal analysis, investigation, and data curation. JH-J: conceptualization, methodology, investigation, and review and editing. J-PM: conceptualization, resources, review and editing, and project administration. All authors contributed to the article and approved the submitted version.

## Conflict of Interest

RR-S was employed by the company Biophyto Plant Health LTDA ME. J-PM and DSB were employed by the company Mars Wrigley. The remaining authors declare that the research was conducted in the absence of any commercial or financial relationships that could be construed as a potential conflict of interest. The authors declare that this research study was supported by Mars Wrigley, United States.

## Footnotes

1 Cabruca is an agroforest where cacao shrubs replaced the original forest understory, while a few canopy trees are left for shade (Faria et al., 2006, 2007).

2 https://www.geneious.com/

3 www.inkscape.org

4 https://github.com/sgelias/phytophthora-on-cocoa
