# Supplementary material for: Phytophthora theobromicola sp. nov.: A New Species Causing Black Pod Disease on Cacao in Brazil
Source: Front Microbiol. 2021 Mar 15;12:537399. doi: 10.3389/fmicb.2021.537399 (PMC8015942; doi:10.3389/fmicb.2021.537399)
Supplement: Supplementary file 1 [file Data_Sheet_1.docx]

*Phytophthora theobromicola* sp. nov. from cacao

**Supplementary Table S1.** GenBank accession numbers of β-tubulin (*BTUB*) partial sequences of *Phytophthora* isolates included in this study.

| **Species** | **Isolate** | **City, State** | **BTUB** |
| --- | --- | --- | --- |
| *P. palmivora* | CCUB 1145 | Barro Preto, BA | MT074255 |
| *P. palmivora* | CCUB 1158^*^ | Barro Preto, BA | MT074248 |
| *P. palmivora* | CCUB 1147 | Barro Preto, BA | MT074251 |
| *P. palmivora* | CCUB 1188 | Barro Preto, BA | MT074256 |
| *P. palmivora* | CCUB 1154 | Barro Preto, BA | MT074252 |
| *P. palmivora* | CCUB 1130 | Barro Preto, BA | MT074257 |
| *P. palmivora* | CCUB 954 | Barro Preto, BA | MT074253 |
| *P. palmivora* | CCUB 943 | Barro Preto, BA | MT074254 |
| *P. palmivora* | CCUB 1102^*^ | Eunápolis, BA | MT074247 |
| *P. palmivora* | CCUB 1146 | Igrapiúna, BA | MT074259 |
| *P. palmivora* | CCUB 920^*^ | Igrapiúna, BA | MT074249 |
| *P. palmivora* | CCUB 1027 | Igrapiúna, BA | MT074260 |
| *P. palmivora* | CCUB 999 | Igrapiúna, BA | MT074261 |
| *P. palmivora* | CCUB 913 | Igrapiúna, BA | MT074262 |
| *P. palmivora* | CCUB 1166 | Igrapiúna, BA | MT074258 |
| *P. palmivora* | CCUB 906^*^ | Porto Seguro, BA | MT074250 |
| *P. theobromicola* | CCUB 997 | Barro Preto, BA | MT074243 |
| *P. theobromicola* | CCUB 1151^*^ | Barro Preto, BA | MT074226 |
| *P. theobromicola* | CCUB 1269 | Eunápolis, BA | MT074237 |
| *P. theobromicola* | - | Eunápolis, BA | MT074238 |
| *P. theobromicola* | CCUB 1129 | Eunápolis, BA | MT074239 |
| *P. theobromicola* | CCUB 1171 | Eunápolis, BA | MT074240 |
| *P. theobromicola* | CCUB 945 | Eunápolis, BA | MT074241 |
| *P. theobromicola* | CCUB 1168 | Eunápolis, BA | MT074242 |
| *P. theobromicola* | CCUB 1091^*^ | Eunápolis, BA | MT074223 |
| *P. theobromicola* | CCUB 1095 | Igrapiúna, BA | MT074244 |
| *P. theobromicola* | CCUB 1205^*^ | Igrapiúna, BA | MT074224 |
| *P. theobromicola* | CCUB 995 | Igrapiúna, BA | MT074227 |
| *P. theobromicola* | CCUB 1160 | Igrapiúna, BA | MT074228 |
| *P. theobromicola* | CCUB 1285^*^ | Porto Seguro, BA | MT074225 |
| *P. theobromicola* | CCUB 1161 | Porto Seguro, BA | MT074229 |
| *P. theobromicola* | CCUB 891 | Porto Seguro, BA | MT074230 |
| *P. theobromicola* | CCUB 1118 | Porto Seguro, BA | MT074231 |
| *P. theobromicola* | CCUB 1208 | Porto Seguro, BA | MT074232 |
| *P. theobromicola* | CCUB 660 | Porto Seguro, BA | MT074233 |
| *P. theobromicola* | CCUB 661 | Porto Seguro, BA | MT074234 |
| *P. theobromicola* | CCUB 1262 | Porto Seguro, BA | MT074235 |
| *P. theobromicola* | CCUB 1287 | Porto Seguro, BA | MT074236 |
| *P. theobromicola* | CCUB 1153 | Eunápolis, BA | MT074245 |
| *P. theobromicola* | - | Eunápolis, BA | MT074246 |

^*^Isolates selected for multigenic analysis.

**Supplementary Table S2.** GenBank accession numbers of DNA sequences of *Phytophthora* isolates used in the multigenic analysis.

| Species name (Year) | ITS clade | Isolate | ITS | COX1 | tub | HSP90 | tef | COX2 |
| --- | --- | --- | --- | --- | --- | --- | --- | --- |
| *P. botryosa* (1969) (15) | 2ª | P3425* | MK496516 | MH136855 | MH493910 | KX250541 | MH358961 | GU221946 |
| *P. botryosa* (1969) (15) | 2ª | P6945 | HQ261507 | HQ261254 | EU079935 | EU079938 | EU079936 | N618611 |
| *P. citrophthora* (1925) | 2ª | P0479* | MG865476 | MH136872 | MH493923 | MK020289 | MH358975 | JN618573 |
| *P. citrophthora* | 2ª | 26H3 | JN605991 | JN605932 | JN605856 | ND | ND | ND |
| *P. colocasiae* (1900) | 2ª | P6317* | MG865479 | MH136875 | MH493926 | MK020291 | MH358978 | JN618667 |
| *P. colocasiae* (1900) | 2ª | P6290 | JN618788 | HQ261287 | EU080125 | ND | EU080126 | GU221984 |
| *P. himalsilva* (2011) | 2ª | P19820* | MG865507 | MH136901 | KX250573 | KX250576 | KX250574 | ND |
| *P. himalsilva* (2011) | 2ª | 61G3 | ND | ND | KX250580 | KX250583 | KX250581 | ND |
| *P. meadii* (1918) (4) | 2ª | P19007* | MG865529 | MH136924 | MH493969 | MK020336 | MH359023 | JN618626 |
| *P. meadii* (1918) (4) | 2ª | P19041 | MG865530 | MH136925 | MH493970 | ND | MH359024 | JN618628 |
| *P. mekongensis* (2017) | 2ª | CBS135136* | KC875838 | KT366920 | ND | ND | ND | ND |
| *P. occultans* (2015) | 2ª | P19955* | MG865555 | MH477753 | MH493990 | MK020359 | MH359045 | ND |
| *P. occultans* (2015) | 2ª | 4953 | KP742989 | KR028484 | KR028483 | ND | ND | ND |
| *P. terminalis* (2015) | 2ª | P19956* | MG865592 | MH136984 | MH494018 | MK020395 | MH359079 | ND |
| *P. terminalis* (2015) | 2ª | CBS131242 | JX978164 | ND | JX978166 | ND | KF650773 | ND |
| *P. amaranthi* (2016) | 2b | CPHST BL 174* | MG783373 | MH477739 | KJ179949 | ND | MK864032 | ND |
| *P. amaranthi* (2016) | 2b | CPHST BL 176 | MG783375 | MH136994 | ND | ND | ND | ND |
| *P. capsici* (1922) | 2b | P1091* | MG865467 | MH136863 | MH493915 | MK020283 | MH358966 | JF771285 |
| *P. glovera* (2011) | 2b | P11685* | MG865500 | MH136895 | MH493943 | MK020310 | MH358995 | ND |
| *P. glovera* (2011) | 2b | P10619 | HQ261689 | HQ261436 | EU080223 | EU080226 | EU080224 | GU222020 |
| *P. mengei* (2009) | 2b | P16862* | MG865539 | MH136932 | MH49397 | MK020344 | MH359033 | ND |
| *P. mexicana* (1923) | 2b | P0646* | MG865540 | MH136933 | MH493978 | MK020345 | MH359034 | GU222075 |
| *P. siskiyouensis* (2008) | 2b | P15122* | MG865586 | MH136978 | KX250678 | KX250681 | KX250679 | GU222137 |
| *P. siskiyouensis* (2008) | 2b | PRI817 | HQ643345 | HQ708393 | ND | ND | ND | ND |
| *P. tropicalis* (2001) | 2b | P10329* | MG865596 | MH136987 | MH494022 | MK020399 | MH359082 | GU222154 |
| *P. tropicalis* (2001) | 2b | P10219 | FJ801320 | ND | EU079994 | ND | EU079995 | JF771615 |
| *P. acerina* (2014) | 2c | P19934* | MG518642 | MH136845 | MH493901 | MK020268 | MH35895 | ND |
| *P. acerina* (2014) | 2c | B060 | JX951287 | KC156136 | KC201285 | ND | ND | ND |
| *P. capensis* (2010) | 2c | P1819* | MG865466 | MH136862 | GU191328 | MK020282 | GU191199 | JQ439415 |
| *P. capensis* (2010) | 2c | P1822 | GU191219 | ND | GU191325 | ND | GU191196 | ND |
| *P. caryae* (2016) | 2c | NJB2013-AF-08* | KJ631538 | KJ631586 | KJ631572 | ND | KU695515 | ND |
| *P. caryae* (2016) | 2c | NJB2013-MF-21 | KJ631540 | KJ631588 | KJ631574 | ND | KU695517 | ND |
| *P. citricola* (1927) | 2c | P0716* | MG865475 | MH136871 | GQ247653 | MK020288 | GQ247662 | GU221973 |
| *P. citricola* (1927) | 2c | P1814 | GU191217 | ND | GU191321 | ND | GU191192 | JQ439419 |
| *P. multivora* (2009) | 2c | P19594* | MG865546 | MH136939 | MN207273 | KX250779 | MK864047 | ND |
| *P. multivora* (2009) | 2c | WAC13204 | FJ237518 | FJ237507 | FJ665259 | ND | ND | ND |
| *P. pachypleura* (2014) | 2c | P19987* | MG865558 | MH136948 | MH493991 | MK020362 | MH359046 | ND |
| *P. pachypleura* (2014) | 2c | RHS2474.2001 | KC855321 | KC855417 | KC855345 | ND | KC855369 | ND |
| *P. pini* (1925) | 2c | P3274* | MG865565 | MH136957 | MH493998 | MK020369 | MH359055 | ND |
| *P. plurivora* (2009) | 2c | P16840* | MG865568 | MH136959 | MH494001 | MK020372 | MH359058 | ND |
| *P. taxon emzansi* | 2c | 61F2* | MH620115 | ND | KX250860 | KX250863 | KX250861 | ND |
| *P. taxon emzansi* | 2c | 61F3 | GU191228 | ND | KX250867 | KX250870 | KX250868 | ND |
| *P. acaciae* (2019) | 2d | AN02* | KX396303 | KX396267 | KX396338 | ND | KX396326 | KX396279 |
| *P. acaciae* (2019) | 2d | AN13 | KX396306 | KX396270 | KX396341 | ND | KX396329 | KX396282 |
| *P. bisheria* (2008) | 2d | P10117* | MG783381 | MH136851 | MH493908 | EU080745 | MH358959 | GU221940 |
| *P. bisheria* (2008) | 2d | P1620 | ND | ND | EU080613 | EU080616 | EU080614 | JF771251 |
| *P. elongata* (2010) | 2d | P19596* | MG865485 | MH136881 | MH493932 | MK020301 | MH358983 | ND |
| *P. elongata* (2010) | 2d | VHS13483 | KX011267 | MN991992 | MN991986 | MN991996 | ND | ND |
| *P. frigida* (2007) (18) | 2d | P16947* | MG865496 | MH136892 | MH493940 | KX250919 | MH358992 | ND |
| *P. frigida* (2007) (18) | 2d | 47G7 | ND | ND | KX250909 | KX250912 | KX250910 | ND |
| *P. oleae* (2018) | 2d | CBS7669* | KY982930 | MF083569 | ND | ND | ND | ND |
| *P. oleae* (2018) | 2d | CBS7670 | KY982934 | MF083573 | ND | ND | ND | ND |
| *P. multivesiculata* (1998) | 2e | P10410* | MG865544 | MH136937 | EU080066 | EU080069 | EU080067 | ND |
| *P. taxon aquatilis* (2012) | 2e | 38J5* | FJ666126 | ND | KX250930 | KX250933 | KX250931 | ND |
| *P. palmivora* (1910) | 4 | P0633 | MG865559 | MH136949 | MH493992 | MK020363 | MH359047 | JF771543 |

^*^Ex-type isolates or reference specimens; ND – Not determined.
