# Supplementary material for: Phytophthora theobromicola sp. nov.: A New Species Causing Black Pod Disease on Cacao in Brazil
Source: Front Microbiol. 2021 Mar 15;12:537399. doi: 10.3389/fmicb.2021.537399 (PMC8015942; doi:10.3389/fmicb.2021.537399)
Supplement: Supplementary file 2 [file Data_Sheet_1.ZIP › essay-1/essay-1.html]

Essay 1 - Culture media growth fitness


# Essay 1 - Culture media growth fitness

- 1 Exploratory data analysis
  - 1.1 Get raw data
  - 1.2 Plot data profile
  - 1.3 Plot observed data as a scatterplot
- 2 Comprobatory data analysis
  - 2.1 Test if essay have some effect on essay
    - 2.1.1 How much the essay is important to explain about the total variance?
  - 2.2 Test if replicate have some effect on essay
    - 2.2.1 How much the replicate is important to explain about the total variance?
  - 2.3 Generate model with strain as predictor
    - 2.3.1 How much the target effects are important to explain about the total variance?
    - 2.3.2 Plot final model adjust
    - 2.3.3 Test significance of fixed effects
    - 2.3.4 Test significance of random effects
    - 2.3.5 Plot a residual histogram
    - 2.3.6 Get general adjust of final model
    - 2.3.7 Plot a scatter for observed vs predicted values
    - 2.3.8 Generate pairwise comparisons
    - 2.3.9 Include alpha-numeric indicators of comparisons significance
    - 2.3.10 Plot multiplicity comparisons
  - 2.4 Generate model with species as predictor
    - 2.4.1 How much the target effects are important to explain about the total variance?
    - 2.4.2 Plot final model adjust
    - 2.4.3 Test significance of fixed effects
    - 2.4.4 Test significance of random effects
    - 2.4.5 Plot a residual histogram
    - 2.4.6 Get general adjust of final model
    - 2.4.7 Plot a scatter for observed vs predicted values
    - 2.4.8 Generate pairwise comparisons
    - 2.4.9 Include alpha-numeric indicators of comparisons significance
    - 2.4.10 Plot multiplicity comparisons

# 1 Exploratory data analysis

## 1.1 Get raw data

## 1.2 Plot data profile

## 1.3 Plot observed data as a scatterplot

# 2 Comprobatory data analysis

## 2.1 Test if essay have some effect on essay

```
## Linear mixed model fit by REML. t-tests use Satterthwaite's method ['lmerModLmerTest']
## Formula: average ~ day:strain:cultureMedia + (1 | essay)
##    Data: dataset
## 
## REML criterion at convergence: 9743.6
## 
## Scaled residuals: 
##     Min      1Q  Median      3Q     Max 
## -3.9421 -0.6048 -0.0574  0.6008  3.2000 
## 
## Random effects:
##  Groups   Name        Variance Std.Dev.
##  essay    (Intercept)  0.5411  0.7356  
##  Residual             13.0373  3.6107  
## Number of obs: 1792, groups:  essay, 2
## 
## Fixed effects:
##                                      Estimate Std. Error        df t value Pr(>|t|)    
## (Intercept)                            4.5496     0.5541    1.2141    8.21   0.0519 .  
## day:strainCCUB 1091:cultureMediaCA    11.2629     0.1145 1754.0391   98.40   <2e-16 ***
## day:strainCCUB 1102:cultureMediaCA     9.0777     0.1145 1754.0391   79.31   <2e-16 ***
## day:strainCCUB 1151:cultureMediaCA    11.3170     0.1145 1754.0391   98.88   <2e-16 ***
## day:strainCCUB 1158:cultureMediaCA     9.1482     0.1145 1754.0391   79.93   <2e-16 ***
## day:strainCCUB 1205:cultureMediaCA    11.0773     0.1145 1754.0391   96.78   <2e-16 ***
## day:strainCCUB 1285:cultureMediaCA    10.7706     0.1145 1754.0391   94.10   <2e-16 ***
## day:strainCCUB 906:cultureMediaCA      8.4300     0.1145 1754.0391   73.65   <2e-16 ***
## day:strainCCUB 920:cultureMediaCA      8.4833     0.1145 1754.0391   74.12   <2e-16 ***
## day:strainP0479:cultureMediaCA        11.5230     0.1145 1754.0391  100.67   <2e-16 ***
## day:strainCCUB 1091:cultureMediaMEA    9.6029     0.1145 1754.0391   83.90   <2e-16 ***
## day:strainCCUB 1102:cultureMediaMEA    4.5380     0.1145 1754.0391   39.65   <2e-16 ***
## day:strainCCUB 1151:cultureMediaMEA   10.5851     0.1145 1754.0391   92.48   <2e-16 ***
## day:strainCCUB 1158:cultureMediaMEA    4.9851     0.1145 1754.0391   43.55   <2e-16 ***
## day:strainCCUB 1205:cultureMediaMEA   10.9018     0.1145 1754.0391   95.25   <2e-16 ***
## day:strainCCUB 1285:cultureMediaMEA   10.8378     0.1145 1754.0391   94.69   <2e-16 ***
## day:strainCCUB 906:cultureMediaMEA     4.5892     0.1145 1754.0391   40.09   <2e-16 ***
## day:strainCCUB 920:cultureMediaMEA     3.1605     0.1145 1754.0391   27.61   <2e-16 ***
## day:strainP0479:cultureMediaMEA        9.5068     0.1145 1754.0391   83.06   <2e-16 ***
## day:strainCCUB 1091:cultureMediaPDA    8.4836     0.1145 1754.0391   74.12   <2e-16 ***
## day:strainCCUB 1102:cultureMediaPDA    2.4886     0.1145 1754.0391   21.74   <2e-16 ***
## day:strainCCUB 1151:cultureMediaPDA    9.2394     0.1145 1754.0391   80.72   <2e-16 ***
## day:strainCCUB 1158:cultureMediaPDA    2.7799     0.1145 1754.0391   24.29   <2e-16 ***
## day:strainCCUB 1205:cultureMediaPDA    8.5950     0.1145 1754.0391   75.09   <2e-16 ***
## day:strainCCUB 1285:cultureMediaPDA    9.0532     0.1145 1754.0391   79.10   <2e-16 ***
## day:strainCCUB 906:cultureMediaPDA     3.5689     0.1145 1754.0391   31.18   <2e-16 ***
## day:strainCCUB 920:cultureMediaPDA     2.3338     0.1145 1754.0391   20.39   <2e-16 ***
## day:strainP0479:cultureMediaPDA        6.0964     0.1145 1754.0391   53.26   <2e-16 ***
## day:strainCCUB 1091:cultureMediaV8    12.0198     0.1580 1754.7580   76.05   <2e-16 ***
## day:strainCCUB 1102:cultureMediaV8     6.2209     0.1580 1754.7580   39.36   <2e-16 ***
## day:strainCCUB 1151:cultureMediaV8    12.1680     0.1580 1754.7580   76.99   <2e-16 ***
## day:strainCCUB 1158:cultureMediaV8     7.4583     0.1580 1754.7580   47.19   <2e-16 ***
## day:strainCCUB 1205:cultureMediaV8    12.3726     0.1580 1754.7580   78.29   <2e-16 ***
## day:strainCCUB 1285:cultureMediaV8    12.3756     0.1580 1754.7580   78.31   <2e-16 ***
## day:strainCCUB 906:cultureMediaV8      6.1953     0.1580 1754.7580   39.20   <2e-16 ***
## day:strainCCUB 920:cultureMediaV8      5.9546     0.1580 1754.7580   37.68   <2e-16 ***
## day:strainP0479:cultureMediaV8        12.4107     0.1145 1754.0391  108.43   <2e-16 ***
## ---
## Signif. codes:  0 '***' 0.001 '**' 0.01 '*' 0.05 '.' 0.1 ' ' 1
```

### 2.1.1 How much the essay is important to explain about the total variance?

```
## [1] "Variance: 3.9850104069 %"
```

## 2.2 Test if replicate have some effect on essay

```
## Linear mixed model fit by REML. t-tests use Satterthwaite's method ['lmerModLmerTest']
## Formula: average ~ day:strain:cultureMedia + (1 | replicate)
##    Data: dataset
## 
## REML criterion at convergence: 9773.1
## 
## Scaled residuals: 
##     Min      1Q  Median      3Q     Max 
## -4.0458 -0.6285 -0.0677  0.6707  3.1090 
## 
## Random effects:
##  Groups    Name        Variance  Std.Dev. 
##  replicate (Intercept) 6.598e-17 8.123e-09
##  Residual              1.329e+01 3.645e+00
## Number of obs: 1792, groups:  replicate, 4
## 
## Fixed effects:
##                                      Estimate Std. Error        df t value Pr(>|t|)    
## (Intercept)                            4.4855     0.1925 1755.0000   23.30   <2e-16 ***
## day:strainCCUB 1091:cultureMediaCA    11.2757     0.1155 1755.0000   97.61   <2e-16 ***
## day:strainCCUB 1102:cultureMediaCA     9.0906     0.1155 1755.0000   78.69   <2e-16 ***
## day:strainCCUB 1151:cultureMediaCA    11.3298     0.1155 1755.0000   98.08   <2e-16 ***
## day:strainCCUB 1158:cultureMediaCA     9.1610     0.1155 1755.0000   79.30   <2e-16 ***
## day:strainCCUB 1205:cultureMediaCA    11.0902     0.1155 1755.0000   96.00   <2e-16 ***
## day:strainCCUB 1285:cultureMediaCA    10.7834     0.1155 1755.0000   93.35   <2e-16 ***
## day:strainCCUB 906:cultureMediaCA      8.4428     0.1155 1755.0000   73.09   <2e-16 ***
## day:strainCCUB 920:cultureMediaCA      8.4962     0.1155 1755.0000   73.55   <2e-16 ***
## day:strainP0479:cultureMediaCA        11.5358     0.1155 1755.0000   99.86   <2e-16 ***
## day:strainCCUB 1091:cultureMediaMEA    9.6157     0.1155 1755.0000   83.24   <2e-16 ***
## day:strainCCUB 1102:cultureMediaMEA    4.5508     0.1155 1755.0000   39.40   <2e-16 ***
## day:strainCCUB 1151:cultureMediaMEA   10.5979     0.1155 1755.0000   91.74   <2e-16 ***
## day:strainCCUB 1158:cultureMediaMEA    4.9979     0.1155 1755.0000   43.27   <2e-16 ***
## day:strainCCUB 1205:cultureMediaMEA   10.9146     0.1155 1755.0000   94.48   <2e-16 ***
## day:strainCCUB 1285:cultureMediaMEA   10.8506     0.1155 1755.0000   93.93   <2e-16 ***
## day:strainCCUB 906:cultureMediaMEA     4.6020     0.1155 1755.0000   39.84   <2e-16 ***
## day:strainCCUB 920:cultureMediaMEA     3.1733     0.1155 1755.0000   27.47   <2e-16 ***
## day:strainP0479:cultureMediaMEA        9.5196     0.1155 1755.0000   82.41   <2e-16 ***
## day:strainCCUB 1091:cultureMediaPDA    8.4964     0.1155 1755.0000   73.55   <2e-16 ***
## day:strainCCUB 1102:cultureMediaPDA    2.5014     0.1155 1755.0000   21.65   <2e-16 ***
## day:strainCCUB 1151:cultureMediaPDA    9.2523     0.1155 1755.0000   80.09   <2e-16 ***
## day:strainCCUB 1158:cultureMediaPDA    2.7928     0.1155 1755.0000   24.18   <2e-16 ***
## day:strainCCUB 1205:cultureMediaPDA    8.6078     0.1155 1755.0000   74.52   <2e-16 ***
## day:strainCCUB 1285:cultureMediaPDA    9.0660     0.1155 1755.0000   78.48   <2e-16 ***
## day:strainCCUB 906:cultureMediaPDA     3.5817     0.1155 1755.0000   31.00   <2e-16 ***
## day:strainCCUB 920:cultureMediaPDA     2.3466     0.1155 1755.0000   20.31   <2e-16 ***
## day:strainP0479:cultureMediaPDA        6.1092     0.1155 1755.0000   52.89   <2e-16 ***
## day:strainCCUB 1091:cultureMediaV8    11.9301     0.1588 1755.0000   75.14   <2e-16 ***
## day:strainCCUB 1102:cultureMediaV8     6.1312     0.1588 1755.0000   38.62   <2e-16 ***
## day:strainCCUB 1151:cultureMediaV8    12.0783     0.1588 1755.0000   76.08   <2e-16 ***
## day:strainCCUB 1158:cultureMediaV8     7.3686     0.1588 1755.0000   46.41   <2e-16 ***
## day:strainCCUB 1205:cultureMediaV8    12.2829     0.1588 1755.0000   77.37   <2e-16 ***
## day:strainCCUB 1285:cultureMediaV8    12.2859     0.1588 1755.0000   77.39   <2e-16 ***
## day:strainCCUB 906:cultureMediaV8      6.1056     0.1588 1755.0000   38.46   <2e-16 ***
## day:strainCCUB 920:cultureMediaV8      5.8649     0.1588 1755.0000   36.94   <2e-16 ***
## day:strainP0479:cultureMediaV8        12.4235     0.1155 1755.0000  107.55   <2e-16 ***
## ---
## Signif. codes:  0 '***' 0.001 '**' 0.01 '*' 0.05 '.' 0.1 ' ' 1
## convergence code: 0
## boundary (singular) fit: see ?isSingular
```

### 2.2.1 How much the replicate is important to explain about the total variance?

```
## [1] "Variance: 0 %"
```

## 2.3 Generate model with strain as predictor

```
## Linear mixed model fit by maximum likelihood . t-tests use Satterthwaite's method ['lmerModLmerTest']
## Formula: average ~ day:code:cultureMedia + (1 | day/strain/cultureMedia)
##    Data: dataset
## 
##      AIC      BIC   logLik deviance df.resid 
##   9339.4   9564.5  -4628.7   9257.4     1751 
## 
## Scaled residuals: 
##     Min      1Q  Median      3Q     Max 
## -4.9707 -0.6296 -0.0444  0.6666  3.3786 
## 
## Random effects:
##  Groups                    Name        Variance Std.Dev.
##  cultureMedia:(strain:day) (Intercept) 2.4503   1.5653  
##  strain:day                (Intercept) 0.3891   0.6238  
##  day                       (Intercept) 2.1151   1.4543  
##  Residual                              8.5968   2.9320  
## Number of obs: 1792, groups:  cultureMedia:(strain:day), 252; strain:day, 63; day, 7
## 
## Fixed effects:
##                                                               Estimate Std. Error      df t value Pr(>|t|)    
## (Intercept)                                                     4.3192     1.2710  6.9982   3.398   0.0115 *  
## day:codeP. citrophthora | P0479:cultureMediaCA                 11.5690     0.3282 12.3649  35.255 8.78e-14 ***
## day:codeP. palmivora | CCUB 1102:cultureMediaCA                 9.1238     0.3282 12.3649  27.804 1.60e-12 ***
## day:codeP. palmivora | CCUB 1158:cultureMediaCA                 9.1943     0.3282 12.3649  28.019 1.46e-12 ***
## day:codeP. palmivora | CCUB 906:cultureMediaCA                  8.4761     0.3282 12.3649  25.830 3.92e-12 ***
## day:codeP. palmivora | CCUB 920:cultureMediaCA                  8.5294     0.3282 12.3649  25.992 3.63e-12 ***
## day:codeP. theobromicola sp. nov. | CCUB 1091:cultureMediaCA   11.3090     0.3282 12.3649  34.463 1.16e-13 ***
## day:codeP. theobromicola sp. nov. | CCUB 1151:cultureMediaCA   11.3631     0.3282 12.3649  34.628 1.09e-13 ***
## day:codeP. theobromicola sp. nov. | CCUB 1205:cultureMediaCA   11.1234     0.3282 12.3649  33.897 1.42e-13 ***
## day:codeP. theobromicola sp. nov. | CCUB 1285:cultureMediaCA   10.8166     0.3282 12.3649  32.962 2.00e-13 ***
## day:codeP. citrophthora | P0479:cultureMediaMEA                 9.5529     0.3282 12.3649  29.111 9.13e-13 ***
## day:codeP. palmivora | CCUB 1102:cultureMediaMEA                4.5841     0.3282 12.3649  13.969 6.11e-09 ***
## day:codeP. palmivora | CCUB 1158:cultureMediaMEA                5.0312     0.3282 12.3649  15.332 2.05e-09 ***
## day:codeP. palmivora | CCUB 906:cultureMediaMEA                 4.6352     0.3282 12.3649  14.125 5.37e-09 ***
## day:codeP. palmivora | CCUB 920:cultureMediaMEA                 3.2066     0.3282 12.3649   9.772 3.58e-07 ***
## day:codeP. theobromicola sp. nov. | CCUB 1091:cultureMediaMEA   9.6490     0.3282 12.3649  29.404 8.08e-13 ***
## day:codeP. theobromicola sp. nov. | CCUB 1151:cultureMediaMEA  10.6312     0.3282 12.3649  32.397 2.47e-13 ***
## day:codeP. theobromicola sp. nov. | CCUB 1205:cultureMediaMEA  10.9478     0.3282 12.3649  33.362 1.73e-13 ***
## day:codeP. theobromicola sp. nov. | CCUB 1285:cultureMediaMEA  10.8839     0.3282 12.3649  33.167 1.85e-13 ***
## day:codeP. citrophthora | P0479:cultureMediaPDA                 6.1425     0.3282 12.3649  18.719 1.91e-10 ***
## day:codeP. palmivora | CCUB 1102:cultureMediaPDA                2.5347     0.3282 12.3649   7.724 4.47e-06 ***
## day:codeP. palmivora | CCUB 1158:cultureMediaPDA                2.8260     0.3282 12.3649   8.612 1.42e-06 ***
## day:codeP. palmivora | CCUB 906:cultureMediaPDA                 3.6150     0.3282 12.3649  11.016 9.38e-08 ***
## day:codeP. palmivora | CCUB 920:cultureMediaPDA                 2.3799     0.3282 12.3649   7.252 8.55e-06 ***
## day:codeP. theobromicola sp. nov. | CCUB 1091:cultureMediaPDA   8.5297     0.3282 12.3649  25.993 3.63e-12 ***
## day:codeP. theobromicola sp. nov. | CCUB 1151:cultureMediaPDA   9.2855     0.3282 12.3649  28.297 1.29e-12 ***
## day:codeP. theobromicola sp. nov. | CCUB 1205:cultureMediaPDA   8.6411     0.3282 12.3649  26.333 3.10e-12 ***
## day:codeP. theobromicola sp. nov. | CCUB 1285:cultureMediaPDA   9.0992     0.3282 12.3649  27.729 1.65e-12 ***
## day:codeP. citrophthora | P0479:cultureMediaV8                 12.4568     0.3282 12.3649  37.961 3.55e-14 ***
## day:codeP. palmivora | CCUB 1102:cultureMediaV8                 6.1645     0.3396 14.1919  18.150 3.20e-11 ***
## day:codeP. palmivora | CCUB 1158:cultureMediaV8                 7.4019     0.3396 14.1919  21.793 2.60e-12 ***
## day:codeP. palmivora | CCUB 906:cultureMediaV8                  6.1388     0.3396 14.1919  18.074 3.39e-11 ***
## day:codeP. palmivora | CCUB 920:cultureMediaV8                  5.8982     0.3396 14.1919  17.366 5.84e-11 ***
## day:codeP. theobromicola sp. nov. | CCUB 1091:cultureMediaV8   11.9633     0.3396 14.1919  35.223 3.22e-15 ***
## day:codeP. theobromicola sp. nov. | CCUB 1151:cultureMediaV8   12.1116     0.3396 14.1919  35.660 2.71e-15 ***
## day:codeP. theobromicola sp. nov. | CCUB 1205:cultureMediaV8   12.3162     0.3396 14.1919  36.262 2.14e-15 ***
## day:codeP. theobromicola sp. nov. | CCUB 1285:cultureMediaV8   12.3192     0.3396 14.1919  36.271 2.13e-15 ***
## ---
## Signif. codes:  0 '***' 0.001 '**' 0.01 '*' 0.05 '.' 0.1 ' ' 1
```

```
## 
## Correlation matrix not shown by default, as p = 37 > 12.
## Use print(x, correlation=TRUE)  or
##     vcov(x)        if you need it
```

### 2.3.1 How much the target effects are important to explain about the total variance?

```
## [1] "Variance cultureMedia:(strain:day): 22.1802613261 %"
```

```
## [1] "Variance strain:day: 4.3303761993 %"
```

```
## [1] "Variance day: 19.7453283585 %"
```

### 2.3.2 Plot final model adjust

### 2.3.3 Test significance of fixed effects

### 2.3.4 Test significance of random effects

### 2.3.5 Plot a residual histogram

### 2.3.6 Get general adjust of final model

### 2.3.7 Plot a scatter for observed vs predicted values

### 2.3.8 Generate pairwise comparisons

### 2.3.9 Include alpha-numeric indicators of comparisons significance

```
##  code                                  cultureMedia day lsmean    SE   df lower.CL upper.CL .group           
##  P. palmivora | CCUB 920               PDA            4   13.8 0.988 51.6     11.9     15.8  1               
##  P. palmivora | CCUB 1102              PDA            4   14.5 0.988 51.6     12.5     16.4  1               
##  P. palmivora | CCUB 1158              PDA            4   15.6 0.988 51.6     13.6     17.6  12              
##  P. palmivora | CCUB 920               MEA            4   17.1 0.988 51.6     15.2     19.1  12              
##  P. palmivora | CCUB 906               PDA            4   18.8 0.988 51.6     16.8     20.8   23             
##  P. palmivora | CCUB 1102              MEA            4   22.7 0.988 51.6     20.7     24.6    34            
##  P. palmivora | CCUB 906               MEA            4   22.9 0.988 51.6     20.9     24.8     4            
##  P. palmivora | CCUB 1158              MEA            4   24.4 0.988 51.6     22.5     26.4     45           
##  P. palmivora | CCUB 920               V8             4   27.9 1.048 68.7     25.8     30.0      56          
##  P. palmivora | CCUB 906               V8             4   28.9 1.048 68.7     26.8     31.0       6          
##  P. citrophthora | P0479               PDA            4   28.9 0.988 51.6     26.9     30.9       6          
##  P. palmivora | CCUB 1102              V8             4   29.0 1.048 68.7     26.9     31.1       6          
##  P. palmivora | CCUB 1158              V8             4   33.9 1.048 68.7     31.8     36.0        7         
##  P. palmivora | CCUB 906               CA             4   38.2 0.988 51.6     36.2     40.2         8        
##  P. palmivora | CCUB 920               CA             4   38.4 0.988 51.6     36.5     40.4         8        
##  P. theobromicola sp. nov. | CCUB 1091 PDA            4   38.4 0.988 51.6     36.5     40.4         8        
##  P. theobromicola sp. nov. | CCUB 1205 PDA            4   38.9 0.988 51.6     36.9     40.9         89       
##  P. theobromicola sp. nov. | CCUB 1285 PDA            4   40.7 0.988 51.6     38.7     42.7         890      
##  P. palmivora | CCUB 1102              CA             4   40.8 0.988 51.6     38.8     42.8         890      
##  P. palmivora | CCUB 1158              CA             4   41.1 0.988 51.6     39.1     43.1         890      
##  P. theobromicola sp. nov. | CCUB 1151 PDA            4   41.5 0.988 51.6     39.5     43.4         890      
##  P. citrophthora | P0479               MEA            4   42.5 0.988 51.6     40.5     44.5          90      
##  P. theobromicola sp. nov. | CCUB 1091 MEA            4   42.9 0.988 51.6     40.9     44.9           0A     
##  P. theobromicola sp. nov. | CCUB 1151 MEA            4   46.8 0.988 51.6     44.9     48.8            AB    
##  P. theobromicola sp. nov. | CCUB 1285 CA             4   47.6 0.988 51.6     45.6     49.6             B    
##  P. theobromicola sp. nov. | CCUB 1285 MEA            4   47.9 0.988 51.6     45.9     49.8             B    
##  P. theobromicola sp. nov. | CCUB 1205 MEA            4   48.1 0.988 51.6     46.1     50.1             BC   
##  P. theobromicola sp. nov. | CCUB 1205 CA             4   48.8 0.988 51.6     46.8     50.8             BCD  
##  P. theobromicola sp. nov. | CCUB 1091 CA             4   49.6 0.988 51.6     47.6     51.5             BCDE 
##  P. theobromicola sp. nov. | CCUB 1151 CA             4   49.8 0.988 51.6     47.8     51.8             BCDE 
##  P. citrophthora | P0479               CA             4   50.6 0.988 51.6     48.6     52.6             BCDEF
##  P. theobromicola sp. nov. | CCUB 1091 V8             4   52.2 1.048 68.7     50.1     54.3              CDEF
##  P. theobromicola sp. nov. | CCUB 1151 V8             4   52.8 1.048 68.7     50.7     54.9               DEF
##  P. theobromicola sp. nov. | CCUB 1205 V8             4   53.6 1.048 68.7     51.5     55.7                EF
##  P. theobromicola sp. nov. | CCUB 1285 V8             4   53.6 1.048 68.7     51.5     55.7                EF
##  P. citrophthora | P0479               V8             4   54.1 0.988 51.6     52.2     56.1                 F
## 
## Degrees-of-freedom method: kenward-roger 
## Confidence level used: 0.95 
## P value adjustment: tukey method for comparing a family of 36 estimates 
## significance level used: alpha = 0.05
```

### 2.3.10 Plot multiplicity comparisons

## 2.4 Generate model with species as predictor

```
## Linear mixed model fit by maximum likelihood . t-tests use Satterthwaite's method ['lmerModLmerTest']
## Formula: average ~ day:species:cultureMedia + (1 | day/species/cultureMedia)
##    Data: dataset
## 
##      AIC      BIC   logLik deviance df.resid 
##   9711.3   9804.7  -4838.7   9677.3     1775 
## 
## Scaled residuals: 
##     Min      1Q  Median      3Q     Max 
## -4.8488 -0.6327 -0.0676  0.6351  3.6960 
## 
## Random effects:
##  Groups                     Name        Variance Std.Dev.
##  cultureMedia:(species:day) (Intercept)  4.0509  2.0127  
##  species:day                (Intercept)  0.5023  0.7087  
##  day                        (Intercept)  2.6148  1.6170  
##  Residual                               11.6782  3.4173  
## Number of obs: 1792, groups:  cultureMedia:(species:day), 84; species:day, 21; day, 7
## 
## Fixed effects:
##                                                      Estimate Std. Error      df t value Pr(>|t|)    
## (Intercept)                                            4.0703     1.5078  6.9938   2.700   0.0307 *  
## day:speciesP. citrophthora:cultureMediaCA             11.6188     0.3906 12.4364  29.746 6.22e-13 ***
## day:speciesP. palmivora:cultureMediaCA                 8.8807     0.3805 11.1936  23.342 7.56e-11 ***
## day:speciesP. theobromicola sp. nov.:cultureMediaCA   11.2028     0.3805 11.1936  29.446 5.84e-12 ***
## day:speciesP. citrophthora:cultureMediaMEA             9.6027     0.3906 12.4364  24.585 6.41e-12 ***
## day:speciesP. palmivora:cultureMediaMEA                4.4140     0.3805 11.1936  11.602 1.39e-07 ***
## day:speciesP. theobromicola sp. nov.:cultureMediaMEA  10.5777     0.3805 11.1936  27.803 1.10e-11 ***
## day:speciesP. citrophthora:cultureMediaPDA             6.1923     0.3906 12.4364  15.853 1.28e-09 ***
## day:speciesP. palmivora:cultureMediaPDA                2.8887     0.3805 11.1936   7.593 9.66e-06 ***
## day:speciesP. theobromicola sp. nov.:cultureMediaPDA   8.9387     0.3805 11.1936  23.495 7.04e-11 ***
## day:speciesP. citrophthora:cultureMediaV8             12.5066     0.3906 12.4364  32.019 2.52e-13 ***
## day:speciesP. palmivora:cultureMediaV8                 6.4506     0.3839 11.6006  16.804 1.68e-09 ***
## day:speciesP. theobromicola sp. nov.:cultureMediaV8   12.2274     0.3839 11.6006  31.853 1.18e-12 ***
## ---
## Signif. codes:  0 '***' 0.001 '**' 0.01 '*' 0.05 '.' 0.1 ' ' 1
```

```
## 
## Correlation matrix not shown by default, as p = 13 > 12.
## Use print(x, correlation=TRUE)  or
##     vcov(x)        if you need it
```

### 2.4.1 How much the target effects are important to explain about the total variance?

```
## [1] "Variance cultureMedia:(species:day): 25.7539401291 %"
```

```
## [1] "Variance species:day: 4.1235528555 %"
```

```
## [1] "Variance day: 18.2944916605 %"
```

### 2.4.2 Plot final model adjust

### 2.4.3 Test significance of fixed effects

### 2.4.4 Test significance of random effects

### 2.4.5 Plot a residual histogram

### 2.4.6 Get general adjust of final model

### 2.4.7 Plot a scatter for observed vs predicted values

### 2.4.8 Generate pairwise comparisons

### 2.4.9 Include alpha-numeric indicators of comparisons significance

```
##  species                   cultureMedia day lsmean   SE   df lower.CL upper.CL .group   
##  P. palmivora              PDA            4   15.6 1.11 38.3     13.4     17.9  1       
##  P. palmivora              MEA            4   21.7 1.11 38.3     19.5     24.0   2      
##  P. citrophthora           PDA            4   28.8 1.17 47.6     26.5     31.2    3     
##  P. palmivora              V8             4   29.9 1.13 41.3     27.6     32.2    3     
##  P. palmivora              CA             4   39.6 1.11 38.3     37.3     41.8     4    
##  P. theobromicola sp. nov. PDA            4   39.8 1.11 38.3     37.6     42.1     4    
##  P. citrophthora           MEA            4   42.5 1.17 47.6     40.1     44.8     45   
##  P. theobromicola sp. nov. MEA            4   46.4 1.11 38.3     44.1     48.6      56  
##  P. theobromicola sp. nov. CA             4   48.9 1.11 38.3     46.6     51.1       67 
##  P. citrophthora           CA             4   50.5 1.17 47.6     48.2     52.9        78
##  P. theobromicola sp. nov. V8             4   53.0 1.13 41.3     50.7     55.3         8
##  P. citrophthora           V8             4   54.1 1.17 47.6     51.7     56.4         8
## 
## Degrees-of-freedom method: kenward-roger 
## Confidence level used: 0.95 
## P value adjustment: tukey method for comparing a family of 12 estimates 
## significance level used: alpha = 0.05
```

### 2.4.10 Plot multiplicity comparisons
