# Supplementary material for: Phytophthora theobromicola sp. nov.: A New Species Causing Black Pod Disease on Cacao in Brazil
Source: Front Microbiol. 2021 Mar 15;12:537399. doi: 10.3389/fmicb.2021.537399 (PMC8015942; doi:10.3389/fmicb.2021.537399)
Supplement: Supplementary file 2 [file Data_Sheet_1.ZIP › essay-2/essay-2.html]

Essay 2 - Temperature growth fitness


# Essay 2 - Temperature growth fitness

- 1 Exploratory data analysis
  - 1.1 Get raw data
  - 1.2 Plot data profile
  - 1.3 Plot observed data as a boxplot with reduced groups iteraction
  - 1.4 Plot observed data as a boxplot with full groups iteraction
  - 1.5 Clear dataset
  - 1.6 Plot observed data as a scatterplot
- 2 Comprobatory data analysis
  - 2.1 Test if essay have some effect on essay
    - 2.1.1 How much the essay is important to explain about the total variance?
  - 2.2 Test if replicate have some effect on essay
    - 2.2.1 How much the replicate is important to explain about the total variance?
  - 2.3 Generate full model
    - 2.3.1 How much the target effects are important to explain about the total variance?
    - 2.3.2 Plot final model adjust
    - 2.3.3 Test significance of fixed effects
    - 2.3.4 Test significance of random effects
    - 2.3.5 Plot a residual histogram
    - 2.3.6 Get general adjust of full model
    - 2.3.7 Plot a scatter for observed vs predicted values
    - 2.3.8 Generate pairwise comparisons
    - 2.3.9 Include alpha-numeric indicators of comparisons significance
    - 2.3.10 Plot multiplicity comparisons
  - 2.4 Generate reduced model
    - 2.4.1 How much the target effects are important to explain about the total variance?
    - 2.4.2 Plot final model adjust
    - 2.4.3 Test significance of fixed effects
    - 2.4.4 Test significance of random effects
    - 2.4.5 Plot a residual histogram
    - 2.4.6 Get general adjust of reduced model
    - 2.4.7 Generate pairwise comparisons
    - 2.4.8 Include alpha-numeric indicators of comparisons significance
    - 2.4.9 Plot multiplicity comparisons
    - 2.4.10 Calculate and plot the mycelial growth speed index (MGSI)
    - 2.4.11 Plot a scatter for observed vs predicted values of reduced model

# 1 Exploratory data analysis

## 1.1 Get raw data

## 1.2 Plot data profile

## 1.3 Plot observed data as a boxplot with reduced groups iteraction

## 1.4 Plot observed data as a boxplot with full groups iteraction

## 1.5 Clear dataset

Remove temperatures 5, 35 and 40.

## 1.6 Plot observed data as a scatterplot

# 2 Comprobatory data analysis

## 2.1 Test if essay have some effect on essay

```
## Linear mixed model fit by REML. t-tests use Satterthwaite's method ['lmerModLmerTest']
## Formula: average ~ day:code:temperatureCategorical + (1 | essay)
##    Data: dataset_clear
## 
## REML criterion at convergence: 14603.3
## 
## Scaled residuals: 
##     Min      1Q  Median      3Q     Max 
## -3.9975 -0.5686 -0.0776  0.5147  4.0486 
## 
## Random effects:
##  Groups   Name        Variance Std.Dev.
##  essay    (Intercept)  0.5009  0.7078  
##  Residual             37.0134  6.0839  
## Number of obs: 2261, groups:  essay, 2
## 
## Fixed effects:
##                                                                          Estimate Std. Error         df t value Pr(>|t|)    
## (Intercept)                                                               8.65905    0.57664    1.53358  15.016   0.0120 *  
## day:codeP. citrophthora | P0479:temperatureCategoricalT2                 -0.36472    0.26439 2214.95177  -1.379   0.1679    
## day:codeP. palmivora | CCUB 1102:temperatureCategoricalT2                 0.21253    0.26439 2214.95177   0.804   0.4216    
## day:codeP. palmivora | CCUB 1158:temperatureCategoricalT2                 0.67323    0.26439 2214.95177   2.546   0.0110 *  
## day:codeP. palmivora | CCUB 906:temperatureCategoricalT2                  0.66985    0.26439 2214.95177   2.534   0.0114 *  
## day:codeP. palmivora | CCUB 920:temperatureCategoricalT2                 -0.04939    0.26439 2214.95177  -0.187   0.8518    
## day:codeP. theobromicola sp. nov. | CCUB 1091:temperatureCategoricalT2    3.36300    0.26439 2214.95177  12.720   <2e-16 ***
## day:codeP. theobromicola sp. nov. | CCUB 1151:temperatureCategoricalT2    3.30983    0.26439 2214.95177  12.519   <2e-16 ***
## day:codeP. theobromicola sp. nov. | CCUB 1205:temperatureCategoricalT2    3.75399    0.26439 2214.95177  14.198   <2e-16 ***
## day:codeP. theobromicola sp. nov. | CCUB 1285:temperatureCategoricalT2    3.44952    0.26439 2214.95177  13.047   <2e-16 ***
## day:codeP. citrophthora | P0479:temperatureCategoricalT3                  7.31091    0.19060 2214.06809  38.357   <2e-16 ***
## day:codeP. palmivora | CCUB 1102:temperatureCategoricalT3                 3.09341    0.19060 2214.06809  16.230   <2e-16 ***
## day:codeP. palmivora | CCUB 1158:temperatureCategoricalT3                 3.79815    0.20270 2214.30221  18.738   <2e-16 ***
## day:codeP. palmivora | CCUB 906:temperatureCategoricalT3                  1.97457    0.19060 2214.06809  10.360   <2e-16 ***
## day:codeP. palmivora | CCUB 920:temperatureCategoricalT3                  2.98261    0.19060 2214.06809  15.648   <2e-16 ***
## day:codeP. theobromicola sp. nov. | CCUB 1091:temperatureCategoricalT3    7.07079    0.19060 2214.06809  37.097   <2e-16 ***
## day:codeP. theobromicola sp. nov. | CCUB 1151:temperatureCategoricalT3    7.35461    0.19060 2214.06808  38.586   <2e-16 ***
## day:codeP. theobromicola sp. nov. | CCUB 1205:temperatureCategoricalT3    8.53267    0.19060 2214.06809  44.767   <2e-16 ***
## day:codeP. theobromicola sp. nov. | CCUB 1285:temperatureCategoricalT3    7.50621    0.19060 2214.06809  39.381   <2e-16 ***
## day:codeP. citrophthora | P0479:temperatureCategoricalT4                 10.76178    0.19060 2214.06808  56.462   <2e-16 ***
## day:codeP. palmivora | CCUB 1102:temperatureCategoricalT4                 6.97996    0.19060 2214.06808  36.620   <2e-16 ***
## day:codeP. palmivora | CCUB 1158:temperatureCategoricalT4                 7.25845    0.19060 2214.06808  38.081   <2e-16 ***
## day:codeP. palmivora | CCUB 906:temperatureCategoricalT4                  6.29788    0.19060 2214.06809  33.042   <2e-16 ***
## day:codeP. palmivora | CCUB 920:temperatureCategoricalT4                  6.39072    0.19060 2214.06809  33.529   <2e-16 ***
## day:codeP. theobromicola sp. nov. | CCUB 1091:temperatureCategoricalT4   10.56745    0.19060 2214.06809  55.442   <2e-16 ***
## day:codeP. theobromicola sp. nov. | CCUB 1151:temperatureCategoricalT4   10.76498    0.19060 2214.06809  56.478   <2e-16 ***
## day:codeP. theobromicola sp. nov. | CCUB 1205:temperatureCategoricalT4   10.99003    0.19060 2214.06809  57.659   <2e-16 ***
## day:codeP. theobromicola sp. nov. | CCUB 1285:temperatureCategoricalT4   10.88153    0.19060 2214.06809  57.090   <2e-16 ***
## day:codeP. citrophthora | P0479:temperatureCategoricalT5                 12.09975    0.19060 2214.06809  63.481   <2e-16 ***
## day:codeP. palmivora | CCUB 1102:temperatureCategoricalT5                11.35802    0.19060 2214.06809  59.590   <2e-16 ***
## day:codeP. palmivora | CCUB 1158:temperatureCategoricalT5                11.91447    0.19060 2214.06809  62.509   <2e-16 ***
## day:codeP. palmivora | CCUB 906:temperatureCategoricalT5                  9.89972    0.19060 2214.06809  51.939   <2e-16 ***
## day:codeP. palmivora | CCUB 920:temperatureCategoricalT5                 11.10866    0.19060 2214.06809  58.282   <2e-16 ***
## day:codeP. theobromicola sp. nov. | CCUB 1091:temperatureCategoricalT5   12.01295    0.19060 2214.06809  63.026   <2e-16 ***
## day:codeP. theobromicola sp. nov. | CCUB 1151:temperatureCategoricalT5   12.06514    0.19060 2214.06809  63.300   <2e-16 ***
## day:codeP. theobromicola sp. nov. | CCUB 1205:temperatureCategoricalT5   12.43774    0.19060 2214.06809  65.255   <2e-16 ***
## day:codeP. theobromicola sp. nov. | CCUB 1285:temperatureCategoricalT5   11.11292    0.19060 2214.06809  58.304   <2e-16 ***
## day:codeP. citrophthora | P0479:temperatureCategoricalT6                 10.75479    0.19060 2214.06808  56.425   <2e-16 ***
## day:codeP. palmivora | CCUB 1102:temperatureCategoricalT6                12.50772    0.19060 2214.06809  65.622   <2e-16 ***
## day:codeP. palmivora | CCUB 1158:temperatureCategoricalT6                12.91566    0.19060 2214.06808  67.762   <2e-16 ***
## day:codeP. palmivora | CCUB 906:temperatureCategoricalT6                 11.58599    0.19060 2214.06809  60.786   <2e-16 ***
## day:codeP. palmivora | CCUB 920:temperatureCategoricalT6                 12.17456    0.19060 2214.06809  63.874   <2e-16 ***
## day:codeP. theobromicola sp. nov. | CCUB 1091:temperatureCategoricalT6   11.95286    0.19060 2214.06809  62.711   <2e-16 ***
## day:codeP. theobromicola sp. nov. | CCUB 1151:temperatureCategoricalT6   12.22160    0.19060 2214.06809  64.121   <2e-16 ***
## day:codeP. theobromicola sp. nov. | CCUB 1205:temperatureCategoricalT6   11.17704    0.19060 2214.06809  58.640   <2e-16 ***
## day:codeP. theobromicola sp. nov. | CCUB 1285:temperatureCategoricalT6   10.33820    0.19060 2214.06809  54.239   <2e-16 ***
## ---
## Signif. codes:  0 '***' 0.001 '**' 0.01 '*' 0.05 '.' 0.1 ' ' 1
```

```
## 
## Correlation matrix not shown by default, as p = 46 > 12.
## Use print(x, correlation=TRUE)  or
##     vcov(x)        if you need it
```

### 2.1.1 How much the essay is important to explain about the total variance?

```
## [1] "Variance: 1.3352825796 %"
```

## 2.2 Test if replicate have some effect on essay

```
## boundary (singular) fit: see ?isSingular
```

```
## Linear mixed model fit by REML. t-tests use Satterthwaite's method ['lmerModLmerTest']
## Formula: average ~ day:strain:temperatureCategorical + (1 | replicate)
##    Data: dataset_clear
## 
## REML criterion at convergence: 14614.4
## 
## Scaled residuals: 
##     Min      1Q  Median      3Q     Max 
## -4.0607 -0.5546 -0.0599  0.5085  3.9533 
## 
## Random effects:
##  Groups    Name        Variance Std.Dev.
##  replicate (Intercept)  0.00    0.000   
##  Residual              37.25    6.103   
## Number of obs: 2261, groups:  replicate, 4
## 
## Fixed effects:
##                                               Estimate Std. Error        df t value Pr(>|t|)    
## (Intercept)                                     8.7114     0.2870 2215.0000  30.354  < 2e-16 ***
## day:strainCCUB 1091:temperatureCategoricalT2    3.4492     0.2642 2215.0000  13.055  < 2e-16 ***
## day:strainCCUB 1102:temperatureCategoricalT2    0.2987     0.2642 2215.0000   1.131  0.25831    
## day:strainCCUB 1151:temperatureCategoricalT2    3.3960     0.2642 2215.0000  12.854  < 2e-16 ***
## day:strainCCUB 1158:temperatureCategoricalT2    0.7594     0.2642 2215.0000   2.874  0.00409 ** 
## day:strainCCUB 1205:temperatureCategoricalT2    3.8402     0.2642 2215.0000  14.535  < 2e-16 ***
## day:strainCCUB 1285:temperatureCategoricalT2    3.5357     0.2642 2215.0000  13.382  < 2e-16 ***
## day:strainCCUB 906:temperatureCategoricalT2     0.7560     0.2642 2215.0000   2.862  0.00425 ** 
## day:strainCCUB 920:temperatureCategoricalT2     0.0368     0.2642 2215.0000   0.139  0.88922    
## day:strainP0479:temperatureCategoricalT2       -0.2785     0.2642 2215.0000  -1.054  0.29192    
## day:strainCCUB 1091:temperatureCategoricalT3    7.0603     0.1912 2215.0000  36.930  < 2e-16 ***
## day:strainCCUB 1102:temperatureCategoricalT3    3.0829     0.1912 2215.0000  16.126  < 2e-16 ***
## day:strainCCUB 1151:temperatureCategoricalT3    7.3441     0.1912 2215.0000  38.415  < 2e-16 ***
## day:strainCCUB 1158:temperatureCategoricalT3    3.7739     0.2032 2215.0000  18.570  < 2e-16 ***
## day:strainCCUB 1205:temperatureCategoricalT3    8.5222     0.1912 2215.0000  44.577  < 2e-16 ***
## day:strainCCUB 1285:temperatureCategoricalT3    7.4957     0.1912 2215.0000  39.208  < 2e-16 ***
## day:strainCCUB 906:temperatureCategoricalT3     1.9641     0.1912 2215.0000  10.274  < 2e-16 ***
## day:strainCCUB 920:temperatureCategoricalT3     2.9721     0.1912 2215.0000  15.546  < 2e-16 ***
## day:strainP0479:temperatureCategoricalT3        7.3004     0.1912 2215.0000  38.186  < 2e-16 ***
## day:strainCCUB 1091:temperatureCategoricalT4   10.5570     0.1912 2215.0000  55.220  < 2e-16 ***
## day:strainCCUB 1102:temperatureCategoricalT4    6.9695     0.1912 2215.0000  36.455  < 2e-16 ***
## day:strainCCUB 1151:temperatureCategoricalT4   10.7545     0.1912 2215.0000  56.253  < 2e-16 ***
## day:strainCCUB 1158:temperatureCategoricalT4    7.2480     0.1912 2215.0000  37.912  < 2e-16 ***
## day:strainCCUB 1205:temperatureCategoricalT4   10.9795     0.1912 2215.0000  57.431  < 2e-16 ***
## day:strainCCUB 1285:temperatureCategoricalT4   10.8711     0.1912 2215.0000  56.863  < 2e-16 ***
## day:strainCCUB 906:temperatureCategoricalT4     6.2874     0.1912 2215.0000  32.887  < 2e-16 ***
## day:strainCCUB 920:temperatureCategoricalT4     6.3802     0.1912 2215.0000  33.373  < 2e-16 ***
## day:strainP0479:temperatureCategoricalT4       10.7513     0.1912 2215.0000  56.237  < 2e-16 ***
## day:strainCCUB 1091:temperatureCategoricalT5   12.0025     0.1912 2215.0000  62.781  < 2e-16 ***
## day:strainCCUB 1102:temperatureCategoricalT5   11.3475     0.1912 2215.0000  59.355  < 2e-16 ***
## day:strainCCUB 1151:temperatureCategoricalT5   12.0547     0.1912 2215.0000  63.054  < 2e-16 ***
## day:strainCCUB 1158:temperatureCategoricalT5   11.9040     0.1912 2215.0000  62.266  < 2e-16 ***
## day:strainCCUB 1205:temperatureCategoricalT5   12.4273     0.1912 2215.0000  65.003  < 2e-16 ***
## day:strainCCUB 1285:temperatureCategoricalT5   11.1024     0.1912 2215.0000  58.073  < 2e-16 ***
## day:strainCCUB 906:temperatureCategoricalT5     9.8893     0.1912 2215.0000  51.728  < 2e-16 ***
## day:strainCCUB 920:temperatureCategoricalT5    11.0982     0.1912 2215.0000  58.051  < 2e-16 ***
## day:strainP0479:temperatureCategoricalT5       12.0893     0.1912 2215.0000  63.235  < 2e-16 ***
## day:strainCCUB 1091:temperatureCategoricalT6   11.9424     0.1912 2215.0000  62.467  < 2e-16 ***
## day:strainCCUB 1102:temperatureCategoricalT6   12.4972     0.1912 2215.0000  65.369  < 2e-16 ***
## day:strainCCUB 1151:temperatureCategoricalT6   12.2111     0.1912 2215.0000  63.873  < 2e-16 ***
## day:strainCCUB 1158:temperatureCategoricalT6   12.9052     0.1912 2215.0000  67.503  < 2e-16 ***
## day:strainCCUB 1205:temperatureCategoricalT6   11.1666     0.1912 2215.0000  58.409  < 2e-16 ***
## day:strainCCUB 1285:temperatureCategoricalT6   10.3277     0.1912 2215.0000  54.021  < 2e-16 ***
## day:strainCCUB 906:temperatureCategoricalT6    11.5755     0.1912 2215.0000  60.548  < 2e-16 ***
## day:strainCCUB 920:temperatureCategoricalT6    12.1641     0.1912 2215.0000  63.627  < 2e-16 ***
## day:strainP0479:temperatureCategoricalT6       10.7443     0.1912 2215.0000  56.200  < 2e-16 ***
## ---
## Signif. codes:  0 '***' 0.001 '**' 0.01 '*' 0.05 '.' 0.1 ' ' 1
```

```
## 
## Correlation matrix not shown by default, as p = 46 > 12.
## Use print(x, correlation=TRUE)  or
##     vcov(x)        if you need it
```

```
## optimizer (nloptwrap) convergence code: 0 (OK)
## boundary (singular) fit: see ?isSingular
```

### 2.2.1 How much the replicate is important to explain about the total variance?

```
## [1] "Variance: 0 %"
```

## 2.3 Generate full model

```
## boundary (singular) fit: see ?isSingular
```

```
## Linear mixed model fit by maximum likelihood . t-tests use Satterthwaite's method ['lmerModLmerTest']
## Formula: average ~ day:code:temperatureCategorical + (1 | day/strain/temperatureCategorical)
##    Data: dataset_clear
## 
##      AIC      BIC   logLik deviance df.resid 
##  13439.7  13725.9  -6669.9  13339.7     2211 
## 
## Scaled residuals: 
##     Min      1Q  Median      3Q     Max 
## -4.7226 -0.4956 -0.0460  0.4779  4.4602 
## 
## Random effects:
##  Groups                              Name        Variance Std.Dev.
##  temperatureCategorical:(strain:day) (Intercept) 12.536   3.541   
##  strain:day                          (Intercept)  0.000   0.000   
##  day                                 (Intercept)  6.042   2.458   
##  Residual                                        16.368   4.046   
## Number of obs: 2261, groups:  temperatureCategorical:(strain:day), 315; strain:day, 63; day, 7
## 
## Fixed effects:
##                                                                        Estimate Std. Error       df t value Pr(>|t|)    
## (Intercept)                                                             8.44567    2.13375  7.00001   3.958 0.005475 ** 
## day:codeP. citrophthora | P0479:temperatureCategoricalT2               -0.22537    0.58655 15.92837  -0.384 0.705895    
## day:codeP. palmivora | CCUB 1102:temperatureCategoricalT2               0.35188    0.58655 15.92837   0.600 0.556999    
## day:codeP. palmivora | CCUB 1158:temperatureCategoricalT2               0.81258    0.58655 15.92837   1.385 0.185040    
## day:codeP. palmivora | CCUB 906:temperatureCategoricalT2                0.80920    0.58655 15.92837   1.380 0.186777    
## day:codeP. palmivora | CCUB 920:temperatureCategoricalT2                0.08996    0.58655 15.92837   0.153 0.880039    
## day:codeP. theobromicola sp. nov. | CCUB 1091:temperatureCategoricalT2  3.50235    0.58655 15.92837   5.971 1.99e-05 ***
## day:codeP. theobromicola sp. nov. | CCUB 1151:temperatureCategoricalT2  3.44918    0.58655 15.92837   5.880 2.37e-05 ***
## day:codeP. theobromicola sp. nov. | CCUB 1205:temperatureCategoricalT2  3.89334    0.58655 15.92837   6.638 5.84e-06 ***
## day:codeP. theobromicola sp. nov. | CCUB 1285:temperatureCategoricalT2  3.58887    0.58655 15.92837   6.119 1.51e-05 ***
## day:codeP. citrophthora | P0479:temperatureCategoricalT3                7.35359    0.57396 14.60444  12.812 2.43e-09 ***
## day:codeP. palmivora | CCUB 1102:temperatureCategoricalT3               3.13608    0.57396 14.60444   5.464 7.18e-05 ***
## day:codeP. palmivora | CCUB 1158:temperatureCategoricalT3               3.82702    0.57578 14.79008   6.647 8.36e-06 ***
## day:codeP. palmivora | CCUB 906:temperatureCategoricalT3                2.01724    0.57396 14.60444   3.515 0.003242 ** 
## day:codeP. palmivora | CCUB 920:temperatureCategoricalT3                3.02529    0.57396 14.60444   5.271 0.000103 ***
## day:codeP. theobromicola sp. nov. | CCUB 1091:temperatureCategoricalT3  7.11347    0.57396 14.60444  12.394 3.80e-09 ***
## day:codeP. theobromicola sp. nov. | CCUB 1151:temperatureCategoricalT3  7.39728    0.57396 14.60444  12.888 2.24e-09 ***
## day:codeP. theobromicola sp. nov. | CCUB 1205:temperatureCategoricalT3  8.57535    0.57396 14.60444  14.941 2.99e-10 ***
## day:codeP. theobromicola sp. nov. | CCUB 1285:temperatureCategoricalT3  7.54889    0.57396 14.60444  13.152 1.70e-09 ***
## day:codeP. citrophthora | P0479:temperatureCategoricalT4               10.80446    0.57396 14.60444  18.824 1.20e-11 ***
## day:codeP. palmivora | CCUB 1102:temperatureCategoricalT4               7.02264    0.57396 14.60444  12.235 4.51e-09 ***
## day:codeP. palmivora | CCUB 1158:temperatureCategoricalT4               7.30112    0.57396 14.60444  12.721 2.67e-09 ***
## day:codeP. palmivora | CCUB 906:temperatureCategoricalT4                6.34055    0.57396 14.60444  11.047 1.75e-08 ***
## day:codeP. palmivora | CCUB 920:temperatureCategoricalT4                6.43339    0.57396 14.60444  11.209 1.44e-08 ***
## day:codeP. theobromicola sp. nov. | CCUB 1091:temperatureCategoricalT4 10.61012    0.57396 14.60444  18.486 1.55e-11 ***
## day:codeP. theobromicola sp. nov. | CCUB 1151:temperatureCategoricalT4 10.80765    0.57396 14.60444  18.830 1.19e-11 ***
## day:codeP. theobromicola sp. nov. | CCUB 1205:temperatureCategoricalT4 11.03270    0.57396 14.60444  19.222 8.93e-12 ***
## day:codeP. theobromicola sp. nov. | CCUB 1285:temperatureCategoricalT4 10.92421    0.57396 14.60444  19.033 1.03e-11 ***
## day:codeP. citrophthora | P0479:temperatureCategoricalT5               12.14242    0.57396 14.60444  21.156 2.31e-12 ***
## day:codeP. palmivora | CCUB 1102:temperatureCategoricalT5              11.40069    0.57396 14.60444  19.863 5.62e-12 ***
## day:codeP. palmivora | CCUB 1158:temperatureCategoricalT5              11.95714    0.57396 14.60444  20.833 2.87e-12 ***
## day:codeP. palmivora | CCUB 906:temperatureCategoricalT5                9.94240    0.57396 14.60444  17.322 3.84e-11 ***
## day:codeP. palmivora | CCUB 920:temperatureCategoricalT5               11.15134    0.57396 14.60444  19.429 7.68e-12 ***
## day:codeP. theobromicola sp. nov. | CCUB 1091:temperatureCategoricalT5 12.05562    0.57396 14.60444  21.004 2.55e-12 ***
## day:codeP. theobromicola sp. nov. | CCUB 1151:temperatureCategoricalT5 12.10781    0.57396 14.60444  21.095 2.40e-12 ***
## day:codeP. theobromicola sp. nov. | CCUB 1205:temperatureCategoricalT5 12.48041    0.57396 14.60444  21.744 1.56e-12 ***
## day:codeP. theobromicola sp. nov. | CCUB 1285:temperatureCategoricalT5 11.15559    0.57396 14.60444  19.436 7.64e-12 ***
## day:codeP. citrophthora | P0479:temperatureCategoricalT6               10.79746    0.57396 14.60444  18.812 1.21e-11 ***
## day:codeP. palmivora | CCUB 1102:temperatureCategoricalT6              12.55039    0.57396 14.60444  21.866 1.44e-12 ***
## day:codeP. palmivora | CCUB 1158:temperatureCategoricalT6              12.95833    0.57396 14.60444  22.577 9.16e-13 ***
## day:codeP. palmivora | CCUB 906:temperatureCategoricalT6               11.62866    0.57396 14.60444  20.260 4.25e-12 ***
## day:codeP. palmivora | CCUB 920:temperatureCategoricalT6               12.21724    0.57396 14.60444  21.286 2.11e-12 ***
## day:codeP. theobromicola sp. nov. | CCUB 1091:temperatureCategoricalT6 11.99554    0.57396 14.60444  20.900 2.74e-12 ***
## day:codeP. theobromicola sp. nov. | CCUB 1151:temperatureCategoricalT6 12.26427    0.57396 14.60444  21.368 2.00e-12 ***
## day:codeP. theobromicola sp. nov. | CCUB 1205:temperatureCategoricalT6 11.21972    0.57396 14.60444  19.548 7.05e-12 ***
## day:codeP. theobromicola sp. nov. | CCUB 1285:temperatureCategoricalT6 10.38087    0.57396 14.60444  18.086 2.10e-11 ***
## ---
## Signif. codes:  0 '***' 0.001 '**' 0.01 '*' 0.05 '.' 0.1 ' ' 1
```

```
## 
## Correlation matrix not shown by default, as p = 46 > 12.
## Use print(x, correlation=TRUE)  or
##     vcov(x)        if you need it
```

```
## optimizer (nloptwrap) convergence code: 0 (OK)
## boundary (singular) fit: see ?isSingular
```

### 2.3.1 How much the target effects are important to explain about the total variance?

```
estimates3 <- as.data.frame(VarCorr(lmer_h1))

cultureMedia_strain_day_variance = estimates3[1, "vcov"]
strain_day_variance = estimates3[2, "vcov"]
day_variance = estimates3[3, "vcov"]
residual_variance3 = estimates3[4, "vcov"]


# cultureMedia_strain_day_variance
paste0(
  "Variance temperatureCategorical:(strain:day): ", 
  round(cultureMedia_strain_day_variance / (cultureMedia_strain_day_variance + residual_variance3) * 100, 10), " %"
)
```

```
## [1] "Variance temperatureCategorical:(strain:day): 43.3723026936 %"
```

```
# strain_day_variance
paste0(
  "Variance strain:day: ", 
  round(strain_day_variance / (strain_day_variance + residual_variance3) * 100, 10), " %"
)
```

```
## [1] "Variance strain:day: 0 %"
```

```
# day_variance
paste0(
  "Variance day: ", 
  round(day_variance / (day_variance + residual_variance3) * 100, 10), " %"
)
```

```
## [1] "Variance day: 26.9605748662 %"
```

### 2.3.2 Plot final model adjust

### 2.3.3 Test significance of fixed effects

### 2.3.4 Test significance of random effects

### 2.3.5 Plot a residual histogram

### 2.3.6 Get general adjust of full model

### 2.3.7 Plot a scatter for observed vs predicted values

### 2.3.8 Generate pairwise comparisons

### 2.3.9 Include alpha-numeric indicators of comparisons significance

```
##  code                                  temperatureCategorical day lsmean   SE   df lower.CL upper.CL .group     
##  P. citrophthora | P0479               T2                       4   7.54 1.84 81.6     3.88     11.2  1         
##  P. palmivora | CCUB 920               T2                       4   8.81 1.84 81.6     5.14     12.5  12        
##  P. palmivora | CCUB 1102              T2                       4   9.85 1.84 81.6     6.18     13.5  12        
##  P. palmivora | CCUB 906               T2                       4  11.68 1.84 81.6     8.01     15.4  12        
##  P. palmivora | CCUB 1158              T2                       4  11.70 1.84 81.6     8.03     15.4  12        
##  P. palmivora | CCUB 906               T3                       4  16.51 1.78 69.1    12.96     20.1   23       
##  P. palmivora | CCUB 920               T3                       4  20.55 1.78 69.1    17.00     24.1    3       
##  P. palmivora | CCUB 1102              T3                       4  20.99 1.78 69.1    17.44     24.5    3       
##  P. theobromicola sp. nov. | CCUB 1151 T2                       4  22.24 1.84 81.6    18.57     25.9    3       
##  P. theobromicola sp. nov. | CCUB 1091 T2                       4  22.46 1.84 81.6    18.79     26.1    3       
##  P. theobromicola sp. nov. | CCUB 1285 T2                       4  22.80 1.84 81.6    19.13     26.5    3       
##  P. palmivora | CCUB 1158              T3                       4  23.75 1.79 70.8    20.19     27.3    3       
##  P. theobromicola sp. nov. | CCUB 1205 T2                       4  24.02 1.84 81.6    20.35     27.7    3       
##  P. palmivora | CCUB 906               T4                       4  33.81 1.78 69.1    30.26     37.4     4      
##  P. palmivora | CCUB 920               T4                       4  34.18 1.78 69.1    30.63     37.7     4      
##  P. palmivora | CCUB 1102              T4                       4  36.54 1.78 69.1    32.99     40.1     45     
##  P. theobromicola sp. nov. | CCUB 1091 T3                       4  36.90 1.78 69.1    33.35     40.4     45     
##  P. palmivora | CCUB 1158              T4                       4  37.65 1.78 69.1    34.10     41.2     45     
##  P. citrophthora | P0479               T3                       4  37.86 1.78 69.1    34.31     41.4     45     
##  P. theobromicola sp. nov. | CCUB 1151 T3                       4  38.03 1.78 69.1    34.48     41.6     45     
##  P. theobromicola sp. nov. | CCUB 1285 T3                       4  38.64 1.78 69.1    35.09     42.2     45     
##  P. theobromicola sp. nov. | CCUB 1205 T3                       4  42.75 1.78 69.1    39.20     46.3      56    
##  P. palmivora | CCUB 906               T5                       4  48.22 1.78 69.1    44.67     51.8       67   
##  P. theobromicola sp. nov. | CCUB 1285 T6                       4  49.97 1.78 69.1    46.42     53.5       678  
##  P. theobromicola sp. nov. | CCUB 1091 T4                       4  50.89 1.78 69.1    47.34     54.4        789 
##  P. citrophthora | P0479               T6                       4  51.64 1.78 69.1    48.09     55.2        789 
##  P. citrophthora | P0479               T4                       4  51.66 1.78 69.1    48.11     55.2        789 
##  P. theobromicola sp. nov. | CCUB 1151 T4                       4  51.68 1.78 69.1    48.13     55.2        789 
##  P. theobromicola sp. nov. | CCUB 1285 T4                       4  52.14 1.78 69.1    48.59     55.7        789 
##  P. theobromicola sp. nov. | CCUB 1205 T4                       4  52.58 1.78 69.1    49.03     56.1        7890
##  P. palmivora | CCUB 920               T5                       4  53.05 1.78 69.1    49.50     56.6        7890
##  P. theobromicola sp. nov. | CCUB 1285 T5                       4  53.07 1.78 69.1    49.52     56.6        7890
##  P. theobromicola sp. nov. | CCUB 1205 T6                       4  53.32 1.78 69.1    49.77     56.9        7890
##  P. palmivora | CCUB 1102              T5                       4  54.05 1.78 69.1    50.50     57.6        7890
##  P. palmivora | CCUB 906               T6                       4  54.96 1.78 69.1    51.41     58.5        7890
##  P. palmivora | CCUB 1158              T5                       4  56.27 1.78 69.1    52.72     59.8         890
##  P. theobromicola sp. nov. | CCUB 1091 T6                       4  56.43 1.78 69.1    52.88     60.0         890
##  P. theobromicola sp. nov. | CCUB 1091 T5                       4  56.67 1.78 69.1    53.12     60.2         890
##  P. theobromicola sp. nov. | CCUB 1151 T5                       4  56.88 1.78 69.1    53.33     60.4         890
##  P. citrophthora | P0479               T5                       4  57.02 1.78 69.1    53.47     60.6         890
##  P. palmivora | CCUB 920               T6                       4  57.31 1.78 69.1    53.76     60.9         890
##  P. theobromicola sp. nov. | CCUB 1151 T6                       4  57.50 1.78 69.1    53.95     61.1         890
##  P. theobromicola sp. nov. | CCUB 1205 T5                       4  58.37 1.78 69.1    54.82     61.9          90
##  P. palmivora | CCUB 1102              T6                       4  58.65 1.78 69.1    55.10     62.2          90
##  P. palmivora | CCUB 1158              T6                       4  60.28 1.78 69.1    56.73     63.8           0
## 
## Degrees-of-freedom method: kenward-roger 
## Confidence level used: 0.95 
## P value adjustment: tukey method for comparing a family of 45 estimates 
## significance level used: alpha = 0.05
```

### 2.3.10 Plot multiplicity comparisons

## 2.4 Generate reduced model

```
## boundary (singular) fit: see ?isSingular
```

```
## Linear mixed model fit by maximum likelihood . t-tests use Satterthwaite's method ['lmerModLmerTest']
## Formula: average ~ day:species:temperatureCategorical + (1 | day/species/temperatureCategorical)
##    Data: dataset_clear
## 
##      AIC      BIC   logLik deviance df.resid 
##  13787.7  13902.2  -6873.9  13747.7     2241 
## 
## Scaled residuals: 
##     Min      1Q  Median      3Q     Max 
## -5.5214 -0.4312  0.0137  0.4880  4.2413 
## 
## Random effects:
##  Groups                               Name        Variance Std.Dev.
##  temperatureCategorical:(species:day) (Intercept) 11.436   3.382   
##  species:day                          (Intercept)  0.000   0.000   
##  day                                  (Intercept)  5.345   2.312   
##  Residual                                         22.859   4.781   
## Number of obs: 2261, groups:  temperatureCategorical:(species:day), 105; species:day, 21; day, 7
## 
## Fixed effects:
##                                                               Estimate Std. Error       df t value Pr(>|t|)    
## (Intercept)                                                    7.72771    2.10660  6.99956   3.668  0.00798 ** 
## day:speciesP. citrophthora:temperatureCategoricalT2           -0.08177    0.58154 16.11629  -0.141  0.88992    
## day:speciesP. palmivora:temperatureCategoricalT2               0.65950    0.55460 13.33177   1.189  0.25513    
## day:speciesP. theobromicola sp. nov.:temperatureCategoricalT2  3.75203    0.55460 13.33177   6.765 1.17e-05 ***
## day:speciesP. citrophthora:temperatureCategoricalT3            7.49718    0.56372 14.23085  13.299 2.02e-09 ***
## day:speciesP. palmivora:temperatureCategoricalT3               3.11837    0.55013 12.90720   5.668 7.90e-05 ***
## day:speciesP. theobromicola sp. nov.:temperatureCategoricalT3  7.80234    0.54998 12.89316  14.187 3.04e-09 ***
## day:speciesP. citrophthora:temperatureCategoricalT4           10.94805    0.56372 14.23085  19.421 1.21e-11 ***
## day:speciesP. palmivora:temperatureCategoricalT4               6.91802    0.54998 12.89316  12.579 1.30e-08 ***
## day:speciesP. theobromicola sp. nov.:temperatureCategoricalT4 10.98726    0.54998 12.89316  19.978 4.42e-11 ***
## day:speciesP. citrophthora:temperatureCategoricalT5           12.28602    0.56372 14.23085  21.794 2.47e-12 ***
## day:speciesP. palmivora:temperatureCategoricalT5              11.25649    0.54998 12.89316  20.467 3.26e-11 ***
## day:speciesP. theobromicola sp. nov.:temperatureCategoricalT5 12.09345    0.54998 12.89316  21.989 1.33e-11 ***
## day:speciesP. citrophthora:temperatureCategoricalT6           10.94106    0.56372 14.23085  19.409 1.22e-11 ***
## day:speciesP. palmivora:temperatureCategoricalT6              12.48225    0.54998 12.89316  22.696 8.90e-12 ***
## day:speciesP. theobromicola sp. nov.:temperatureCategoricalT6 11.60869    0.54998 12.89316  21.108 2.22e-11 ***
## ---
## Signif. codes:  0 '***' 0.001 '**' 0.01 '*' 0.05 '.' 0.1 ' ' 1
```

```
## 
## Correlation matrix not shown by default, as p = 16 > 12.
## Use print(x, correlation=TRUE)  or
##     vcov(x)        if you need it
```

```
## optimizer (nloptwrap) convergence code: 0 (OK)
## boundary (singular) fit: see ?isSingular
```

### 2.4.1 How much the target effects are important to explain about the total variance?

```
## [1] "Variance temperatureCategorical:(strain:day): 33.345464333 %"
```

```
## [1] "Variance strain:day: 0 %"
```

```
## [1] "Variance day: 18.9502856625 %"
```

### 2.4.2 Plot final model adjust

### 2.4.3 Test significance of fixed effects

### 2.4.4 Test significance of random effects

### 2.4.5 Plot a residual histogram

### 2.4.6 Get general adjust of reduced model

### 2.4.7 Generate pairwise comparisons

### 2.4.8 Include alpha-numeric indicators of comparisons significance

```
##  species                   temperatureCategorical day lsmean   SE   df lower.CL upper.CL .group
##  P. citrophthora           T2                       4    7.4 1.82 74.0     3.77     11.0  1    
##  P. palmivora              T2                       4   10.4 1.68 52.1     6.99     13.7  1    
##  P. palmivora              T3                       4   20.2 1.66 48.9    16.87     23.5   2   
##  P. theobromicola sp. nov. T2                       4   22.7 1.68 52.1    19.36     26.1   2   
##  P. palmivora              T4                       4   35.4 1.66 48.8    32.07     38.7    3  
##  P. citrophthora           T3                       4   37.7 1.73 58.9    34.25     41.2    3  
##  P. theobromicola sp. nov. T3                       4   38.9 1.66 48.8    35.60     42.3    3  
##  P. citrophthora           T6                       4   51.5 1.73 58.9    48.03     55.0     4 
##  P. citrophthora           T4                       4   51.5 1.73 58.9    48.06     55.0     4 
##  P. theobromicola sp. nov. T4                       4   51.7 1.66 48.8    48.34     55.0     4 
##  P. palmivora              T5                       4   52.8 1.66 48.8    49.42     56.1     4 
##  P. theobromicola sp. nov. T6                       4   54.2 1.66 48.8    50.83     57.5     4 
##  P. theobromicola sp. nov. T5                       4   56.1 1.66 48.8    52.77     59.4     4 
##  P. citrophthora           T5                       4   56.9 1.73 58.9    53.41     60.3     4 
##  P. palmivora              T6                       4   57.7 1.66 48.8    54.32     61.0     4 
## 
## Degrees-of-freedom method: kenward-roger 
## Confidence level used: 0.95 
## P value adjustment: tukey method for comparing a family of 15 estimates 
## significance level used: alpha = 0.05
```

### 2.4.9 Plot multiplicity comparisons

### 2.4.10 Calculate and plot the mycelial growth speed index (MGSI)

### 2.4.11 Plot a scatter for observed vs predicted values of reduced model
