# Supplementary material for: Phytophthora theobromicola sp. nov.: A New Species Causing Black Pod Disease on Cacao in Brazil
Source: Front Microbiol. 2021 Mar 15;12:537399. doi: 10.3389/fmicb.2021.537399 (PMC8015942; doi:10.3389/fmicb.2021.537399)
Supplement: Supplementary file 2 [file Data_Sheet_1.ZIP › essay-3/essay-3.nb.html]

R Notebook


Code 

- Show All Code
- Hide All Code
- Download Rmd

# R Notebook

This is an R Markdown Notebook. When you execute code within the notebook, the results appear beneath the code.

Try executing this chunk by clicking the *Run* button within the chunk or by placing your cursor inside it and pressing *Ctrl+Shift+Enter*.


```
plot(cars)
```


Add a new chunk by clicking the *Insert Chunk* button on the toolbar or by pressing *Ctrl+Alt+I*.

When you save the notebook, an HTML file containing the code and output will be saved alongside it (click the *Preview* button or press *Ctrl+Shift+K* to preview the HTML file).

The preview shows you a rendered HTML copy of the contents of the editor. Consequently, unlike *Knit*, *Preview* does not run any R code chunks. Instead, the output of the chunk when it was last run in the editor is displayed.

LS0tCnRpdGxlOiAiUiBOb3RlYm9vayIKb3V0cHV0OiBodG1sX25vdGVib29rCi0tLQoKVGhpcyBpcyBhbiBbUiBNYXJrZG93bl0oaHR0cDovL3JtYXJrZG93bi5yc3R1ZGlvLmNvbSkgTm90ZWJvb2suIFdoZW4geW91IGV4ZWN1dGUgY29kZSB3aXRoaW4gdGhlIG5vdGVib29rLCB0aGUgcmVzdWx0cyBhcHBlYXIgYmVuZWF0aCB0aGUgY29kZS4gCgpUcnkgZXhlY3V0aW5nIHRoaXMgY2h1bmsgYnkgY2xpY2tpbmcgdGhlICpSdW4qIGJ1dHRvbiB3aXRoaW4gdGhlIGNodW5rIG9yIGJ5IHBsYWNpbmcgeW91ciBjdXJzb3IgaW5zaWRlIGl0IGFuZCBwcmVzc2luZyAqQ3RybCtTaGlmdCtFbnRlciouIAoKYGBge3J9CnBsb3QoY2FycykKYGBgCgpBZGQgYSBuZXcgY2h1bmsgYnkgY2xpY2tpbmcgdGhlICpJbnNlcnQgQ2h1bmsqIGJ1dHRvbiBvbiB0aGUgdG9vbGJhciBvciBieSBwcmVzc2luZyAqQ3RybCtBbHQrSSouCgpXaGVuIHlvdSBzYXZlIHRoZSBub3RlYm9vaywgYW4gSFRNTCBmaWxlIGNvbnRhaW5pbmcgdGhlIGNvZGUgYW5kIG91dHB1dCB3aWxsIGJlIHNhdmVkIGFsb25nc2lkZSBpdCAoY2xpY2sgdGhlICpQcmV2aWV3KiBidXR0b24gb3IgcHJlc3MgKkN0cmwrU2hpZnQrSyogdG8gcHJldmlldyB0aGUgSFRNTCBmaWxlKS4KClRoZSBwcmV2aWV3IHNob3dzIHlvdSBhIHJlbmRlcmVkIEhUTUwgY29weSBvZiB0aGUgY29udGVudHMgb2YgdGhlIGVkaXRvci4gQ29uc2VxdWVudGx5LCB1bmxpa2UgKktuaXQqLCAqUHJldmlldyogZG9lcyBub3QgcnVuIGFueSBSIGNvZGUgY2h1bmtzLiBJbnN0ZWFkLCB0aGUgb3V0cHV0IG9mIHRoZSBjaHVuayB3aGVuIGl0IHdhcyBsYXN0IHJ1biBpbiB0aGUgZWRpdG9yIGlzIGRpc3BsYXllZC4K
