# Supplementary material for: Phytophthora theobromicola sp. nov.: A New Species Causing Black Pod Disease on Cacao in Brazil
Source: Front Microbiol. 2021 Mar 15;12:537399. doi: 10.3389/fmicb.2021.537399 (PMC8015942; doi:10.3389/fmicb.2021.537399)
Supplement: Supplementary file 2 [file Data_Sheet_1.ZIP › essay-3/essay-3.html]

Essay 1 - Culture media growth fitness


# Essay 1 - Culture media growth fitness

- 1 Exploratory data analysis
  - 1.1 Get raw data
  - 1.2 Plot data profile
  - 1.3 Remove Control from raw dataset
  - 1.4 Plot observed data as a scatterplot
  - 1.5 Clear dataset
  - 1.6 Plot observed data as a scatterplot
- 2 Comprobatory data analysis
  - 2.1 Test if essay have some effect on essay
    - 2.1.1 How much the essay is important to explain about the total variance?
  - 2.2 Test if replicate have some effect on essay
    - 2.2.1 How much the replicate is important to explain about the total variance?
  - 2.3 Generate full model
    - 2.3.1 Plot final model adjust
    - 2.3.2 Test significance of fixed effects
    - 2.3.3 Test significance of random effects
    - 2.3.4 Plot a residual histogram
    - 2.3.5 Plot observed vs predicted data as a scatterplot
  - 2.4 Perform a second clean to dataset
    - 2.4.1 Plot observed reduced data as a scatterplot
  - 2.5 Generate reduced model
    - 2.5.1 Plot final model adjust
    - 2.5.2 Test significance of fixed effects
    - 2.5.3 Test significance of random effects
    - 2.5.4 Plot a residual histogram
  - 2.6 Plot observed vs predicted with reduced data as a scatterplot
    - 2.6.1 Generate pairwise comparisons
    - 2.6.2 Include alpha-numeric indicators of comparisons significance
    - 2.6.3 Plot multiplicity comparisons
    - 2.6.4 Plot estimates

# 1 Exploratory data analysis

## 1.1 Get raw data

## 1.2 Plot data profile

## 1.3 Remove Control from raw dataset

## 1.4 Plot observed data as a scatterplot

## 1.5 Clear dataset

Remove values with sd close to zero.

## 1.6 Plot observed data as a scatterplot

# 2 Comprobatory data analysis

## 2.1 Test if essay have some effect on essay

```
## Linear mixed model fit by REML. t-tests use Satterthwaite's method ['lmerModLmerTest']
## Formula: average ~ day:code:inocPosByClone + (1 | essay)
##    Data: dataset_clean
## 
## REML criterion at convergence: 14728.9
## 
## Scaled residuals: 
##     Min      1Q  Median      3Q     Max 
## -4.5418 -0.4878  0.0130  0.4983  5.8274 
## 
## Random effects:
##  Groups   Name        Variance Std.Dev.
##  essay    (Intercept) 223.7    14.96   
##  Residual             181.6    13.48   
## Number of obs: 1850, groups:  essay, 2
## 
## Fixed effects:
##                                                                        Estimate Std. Error        df t value Pr(>|t|)    
## (Intercept)                                                             -1.1659    10.6022    1.0078  -0.110 0.930165    
## day:codeP. palmivora | 1102:inocPosByCloneCCN51 | A                     13.5564     0.7686 1784.0004  17.639  < 2e-16 ***
## day:codeP. palmivora | 1158:inocPosByCloneCCN51 | A                     14.2398     0.8377 1784.0053  16.999  < 2e-16 ***
## day:codeP. palmivora | 906:inocPosByCloneCCN51 | A                      10.8872     1.0741 1784.0385  10.136  < 2e-16 ***
## day:codeP. palmivora | 920:inocPosByCloneCCN51 | A                       9.5624     0.8377 1784.0053  11.415  < 2e-16 ***
## day:codeP. theobromicola sp. nov. | 1091:inocPosByCloneCCN51 | A        20.1468     0.7156 1784.0006  28.154  < 2e-16 ***
## day:codeP. theobromicola sp. nov. | 1151:inocPosByCloneCCN51 | A        17.8950     0.7156 1784.0006  25.007  < 2e-16 ***
## day:codeP. theobromicola sp. nov. | 1205:inocPosByCloneCCN51 | A        15.0331     0.7686 1784.0004  19.560  < 2e-16 ***
## day:codeP. theobromicola sp. nov. | 1285:inocPosByCloneCCN51 | A        16.1768     0.7686 1784.0004  21.048  < 2e-16 ***
## day:codeP. palmivora | 1102:inocPosByCloneCCN51 | B                     15.1860     0.8377 1784.0053  18.128  < 2e-16 ***
## day:codeP. palmivora | 1158:inocPosByCloneCCN51 | B                     14.0754     0.7156 1784.0006  19.670  < 2e-16 ***
## day:codeP. palmivora | 906:inocPosByCloneCCN51 | B                       3.8328     1.0682 1784.0002   3.588 0.000342 ***
## day:codeP. palmivora | 920:inocPosByCloneCCN51 | B                       9.0415     0.8371 1784.0001  10.801  < 2e-16 ***
## day:codeP. theobromicola sp. nov. | 1091:inocPosByCloneCCN51 | B        19.6417     0.7686 1784.0004  25.557  < 2e-16 ***
## day:codeP. theobromicola sp. nov. | 1151:inocPosByCloneCCN51 | B        18.6281     0.7686 1784.0004  24.238  < 2e-16 ***
## day:codeP. theobromicola sp. nov. | 1205:inocPosByCloneCCN51 | B        15.8744     0.7156 1784.0006  22.184  < 2e-16 ***
## day:codeP. theobromicola sp. nov. | 1285:inocPosByCloneCCN51 | B        18.8428     0.7156 1784.0006  26.332  < 2e-16 ***
## day:codeP. palmivora | 1102:inocPosByCloneCepec_2004 | A                12.2822     0.8377 1784.0053  14.662  < 2e-16 ***
## day:codeP. palmivora | 1158:inocPosByCloneCepec_2004 | A                13.2649     0.9330 1784.0190  14.217  < 2e-16 ***
## day:codeP. palmivora | 906:inocPosByCloneCepec_2004 | A                  4.9820     1.8318 1784.0133   2.720 0.006598 ** 
## day:codeP. palmivora | 920:inocPosByCloneCepec_2004 | A                  4.4176     0.7686 1784.0004   5.748 1.06e-08 ***
## day:codeP. theobromicola sp. nov. | 1091:inocPosByCloneCepec_2004 | A   15.7829     0.8377 1784.0053  18.841  < 2e-16 ***
## day:codeP. theobromicola sp. nov. | 1151:inocPosByCloneCepec_2004 | A   19.4419     0.7156 1784.0006  27.169  < 2e-16 ***
## day:codeP. theobromicola sp. nov. | 1205:inocPosByCloneCepec_2004 | A   19.4731     0.7156 1784.0006  27.213  < 2e-16 ***
## day:codeP. theobromicola sp. nov. | 1285:inocPosByCloneCepec_2004 | A   18.3339     0.7156 1784.0006  25.621  < 2e-16 ***
## day:codeP. palmivora | 1102:inocPosByCloneCepec_2004 | B                12.3770     0.7686 1784.0004  16.104  < 2e-16 ***
## day:codeP. palmivora | 1158:inocPosByCloneCepec_2004 | B                12.9607     0.7686 1784.0004  16.864  < 2e-16 ***
## day:codeP. palmivora | 906:inocPosByCloneCepec_2004 | B                 13.5790     1.8318 1784.0133   7.413 1.90e-13 ***
## day:codeP. palmivora | 920:inocPosByCloneCepec_2004 | B                  8.8235     0.8377 1784.0053  10.533  < 2e-16 ***
## day:codeP. theobromicola sp. nov. | 1091:inocPosByCloneCepec_2004 | B   16.8578     0.7686 1784.0004  21.934  < 2e-16 ***
## day:codeP. theobromicola sp. nov. | 1151:inocPosByCloneCepec_2004 | B   18.5066     0.7156 1784.0006  25.862  < 2e-16 ***
## day:codeP. theobromicola sp. nov. | 1205:inocPosByCloneCepec_2004 | B   15.4703     0.7156 1784.0006  21.619  < 2e-16 ***
## day:codeP. theobromicola sp. nov. | 1285:inocPosByCloneCepec_2004 | B   17.9525     0.7686 1784.0004  23.359  < 2e-16 ***
## day:codeP. palmivora | 1102:inocPosByCloneCP49 | A                      13.9143     0.7686 1784.0004  18.104  < 2e-16 ***
## day:codeP. palmivora | 1158:inocPosByCloneCP49 | A                      13.9541     0.7156 1784.0006  19.500  < 2e-16 ***
## day:codeP. palmivora | 906:inocPosByCloneCP49 | A                        4.2489     0.8380 1784.0081   5.070 4.39e-07 ***
## day:codeP. palmivora | 920:inocPosByCloneCP49 | A                        6.6549     0.9308 1784.0022   7.150 1.26e-12 ***
## day:codeP. theobromicola sp. nov. | 1091:inocPosByCloneCP49 | A         19.8004     0.7156 1784.0006  27.670  < 2e-16 ***
## day:codeP. theobromicola sp. nov. | 1151:inocPosByCloneCP49 | A         18.0389     0.7156 1784.0006  25.209  < 2e-16 ***
## day:codeP. theobromicola sp. nov. | 1205:inocPosByCloneCP49 | A         17.1462     0.7156 1784.0006  23.961  < 2e-16 ***
## day:codeP. theobromicola sp. nov. | 1285:inocPosByCloneCP49 | A         18.7911     0.7156 1784.0006  26.260  < 2e-16 ***
## day:codeP. palmivora | 1102:inocPosByCloneCP49 | B                      13.5646     0.7156 1784.0006  18.956  < 2e-16 ***
## day:codeP. palmivora | 1158:inocPosByCloneCP49 | B                      11.4188     0.7156 1784.0006  15.957  < 2e-16 ***
## day:codeP. palmivora | 906:inocPosByCloneCP49 | B                        2.9362     1.0682 1784.0002   2.749 0.006045 ** 
## day:codeP. palmivora | 920:inocPosByCloneCP49 | B                        5.8241     0.9330 1784.0190   6.242 5.38e-10 ***
## day:codeP. theobromicola sp. nov. | 1091:inocPosByCloneCP49 | B         22.3973     0.7156 1784.0006  31.299  < 2e-16 ***
## day:codeP. theobromicola sp. nov. | 1151:inocPosByCloneCP49 | B         17.7299     0.7156 1784.0006  24.777  < 2e-16 ***
## day:codeP. theobromicola sp. nov. | 1205:inocPosByCloneCP49 | B         20.5629     0.7686 1784.0004  26.755  < 2e-16 ***
## day:codeP. theobromicola sp. nov. | 1285:inocPosByCloneCP49 | B         17.0820     0.7156 1784.0006  23.871  < 2e-16 ***
## day:codeP. palmivora | 1102:inocPosByClonePS1319 | A                    12.2811     0.7686 1784.0004  15.979  < 2e-16 ***
## day:codeP. palmivora | 1158:inocPosByClonePS1319 | A                    13.2676     0.7156 1784.0006  18.541  < 2e-16 ***
## day:codeP. palmivora | 906:inocPosByClonePS1319 | A                      7.8100     1.0695 1784.0084   7.303 4.23e-13 ***
## day:codeP. palmivora | 920:inocPosByClonePS1319 | A                      8.6606     0.9330 1784.0190   9.282  < 2e-16 ***
## day:codeP. theobromicola sp. nov. | 1091:inocPosByClonePS1319 | A       18.0995     0.7156 1784.0006  25.293  < 2e-16 ***
## day:codeP. theobromicola sp. nov. | 1151:inocPosByClonePS1319 | A       17.6890     0.7156 1784.0006  24.720  < 2e-16 ***
## day:codeP. theobromicola sp. nov. | 1205:inocPosByClonePS1319 | A       19.6421     0.7156 1784.0006  27.449  < 2e-16 ***
## day:codeP. theobromicola sp. nov. | 1285:inocPosByClonePS1319 | A       15.3929     0.7686 1784.0004  20.028  < 2e-16 ***
## day:codeP. palmivora | 1102:inocPosByClonePS1319 | B                    13.9583     0.8377 1784.0053  16.663  < 2e-16 ***
## day:codeP. palmivora | 1158:inocPosByClonePS1319 | B                    11.9219     0.7156 1784.0006  16.660  < 2e-16 ***
## day:codeP. palmivora | 906:inocPosByClonePS1319 | B                      2.7071     1.3008 1784.0010   2.081 0.037561 *  
## day:codeP. palmivora | 920:inocPosByClonePS1319 | B                      5.8007     0.7686 1784.0004   7.548 7.03e-14 ***
## day:codeP. theobromicola sp. nov. | 1091:inocPosByClonePS1319 | B       20.5787     0.7156 1784.0006  28.758  < 2e-16 ***
## day:codeP. theobromicola sp. nov. | 1151:inocPosByClonePS1319 | B       16.5238     0.7156 1784.0006  23.091  < 2e-16 ***
## day:codeP. theobromicola sp. nov. | 1205:inocPosByClonePS1319 | B       16.2955     0.7156 1784.0006  22.772  < 2e-16 ***
## day:codeP. theobromicola sp. nov. | 1285:inocPosByClonePS1319 | B       17.9557     0.7156 1784.0006  25.092  < 2e-16 ***
## ---
## Signif. codes:  0 '***' 0.001 '**' 0.01 '*' 0.05 '.' 0.1 ' ' 1
```

```
## 
## Correlation matrix not shown by default, as p = 65 > 12.
## Use print(x, correlation=TRUE)  or
##     vcov(x)        if you need it
```

### 2.1.1 How much the essay is important to explain about the total variance?

```
## [1] "Variance: 55.1895378682 %"
```

## 2.2 Test if replicate have some effect on essay

```
## Linear mixed model fit by REML. t-tests use Satterthwaite's method ['lmerModLmerTest']
## Formula: average ~ day:code:inocPosByClone + (1 | replicate)
##    Data: dataset_clean
## 
## REML criterion at convergence: 15469.4
## 
## Scaled residuals: 
##     Min      1Q  Median      3Q     Max 
## -4.3076 -0.5044  0.1467  0.6544  3.9949 
## 
## Random effects:
##  Groups    Name        Variance Std.Dev.
##  replicate (Intercept)  18.93    4.351  
##  Residual              274.51   16.568  
## Number of obs: 1850, groups:  replicate, 4
## 
## Fixed effects:
##                                                                        Estimate Std. Error        df t value Pr(>|t|)    
## (Intercept)                                                              2.2749     2.3580    3.7770   0.965 0.392288    
## day:codeP. palmivora | 1102:inocPosByCloneCCN51 | A                     13.7565     0.9452 1782.0232  14.554  < 2e-16 ***
## day:codeP. palmivora | 1158:inocPosByCloneCCN51 | A                     15.1689     1.0296 1782.0591  14.732  < 2e-16 ***
## day:codeP. palmivora | 906:inocPosByCloneCCN51 | A                       7.6447     1.3139 1782.1089   5.818 7.03e-09 ***
## day:codeP. palmivora | 920:inocPosByCloneCCN51 | A                      10.4881     1.0296 1782.0529  10.186  < 2e-16 ***
## day:codeP. theobromicola sp. nov. | 1091:inocPosByCloneCCN51 | A        19.8684     0.8796 1781.9284  22.587  < 2e-16 ***
## day:codeP. theobromicola sp. nov. | 1151:inocPosByCloneCCN51 | A        17.6165     0.8796 1781.9284  20.027  < 2e-16 ***
## day:codeP. theobromicola sp. nov. | 1205:inocPosByCloneCCN51 | A        15.2332     0.9452 1782.0232  16.117  < 2e-16 ***
## day:codeP. theobromicola sp. nov. | 1285:inocPosByCloneCCN51 | A        16.3741     0.9452 1782.0190  17.324  < 2e-16 ***
## day:codeP. palmivora | 1102:inocPosByCloneCCN51 | B                     16.1151     1.0296 1782.0591  15.651  < 2e-16 ***
## day:codeP. palmivora | 1158:inocPosByCloneCCN51 | B                     13.7970     0.8796 1781.9284  15.685  < 2e-16 ***
## day:codeP. palmivora | 906:inocPosByCloneCCN51 | B                       4.4343     1.3139 1782.1089   3.375 0.000754 ***
## day:codeP. palmivora | 920:inocPosByCloneCCN51 | B                       9.2367     1.0305 1782.3210   8.963  < 2e-16 ***
## day:codeP. theobromicola sp. nov. | 1091:inocPosByCloneCCN51 | B        19.8910     0.9451 1782.0058  21.046  < 2e-16 ***
## day:codeP. theobromicola sp. nov. | 1151:inocPosByCloneCCN51 | B        18.8254     0.9452 1782.0190  19.917  < 2e-16 ***
## day:codeP. theobromicola sp. nov. | 1205:inocPosByCloneCCN51 | B        15.5959     0.8796 1781.9284  17.730  < 2e-16 ***
## day:codeP. theobromicola sp. nov. | 1285:inocPosByCloneCCN51 | B        18.5644     0.8796 1781.9284  21.105  < 2e-16 ***
## day:codeP. palmivora | 1102:inocPosByCloneCepec_2004 | A                13.2113     1.0296 1782.0591  12.831  < 2e-16 ***
## day:codeP. palmivora | 1158:inocPosByCloneCepec_2004 | A                15.2096     1.1443 1782.0394  13.292  < 2e-16 ***
## day:codeP. palmivora | 906:inocPosByCloneCepec_2004 | A                  1.8319     2.2537 1782.4790   0.813 0.416414    
## day:codeP. palmivora | 920:inocPosByCloneCepec_2004 | A                  4.6668     0.9451 1782.0058   4.938 8.64e-07 ***
## day:codeP. theobromicola sp. nov. | 1091:inocPosByCloneCepec_2004 | A   16.6497     1.0297 1782.0784  16.169  < 2e-16 ***
## day:codeP. theobromicola sp. nov. | 1151:inocPosByCloneCepec_2004 | A   19.1635     0.8796 1781.9284  21.786  < 2e-16 ***
## day:codeP. theobromicola sp. nov. | 1205:inocPosByCloneCepec_2004 | A   19.1947     0.8796 1781.9284  21.822  < 2e-16 ***
## day:codeP. theobromicola sp. nov. | 1285:inocPosByCloneCepec_2004 | A   18.0555     0.8796 1781.9284  20.526  < 2e-16 ***
## day:codeP. palmivora | 1102:inocPosByCloneCepec_2004 | B                12.6262     0.9451 1782.0058  13.360  < 2e-16 ***
## day:codeP. palmivora | 1158:inocPosByCloneCepec_2004 | B                13.1581     0.9452 1782.0190  13.921  < 2e-16 ***
## day:codeP. palmivora | 906:inocPosByCloneCepec_2004 | B                 10.4289     2.2537 1782.4790   4.628 3.97e-06 ***
## day:codeP. palmivora | 920:inocPosByCloneCepec_2004 | B                  9.7526     1.0296 1782.0591   9.472  < 2e-16 ***
## day:codeP. theobromicola sp. nov. | 1091:inocPosByCloneCepec_2004 | B   17.1070     0.9451 1782.0058  18.101  < 2e-16 ***
## day:codeP. theobromicola sp. nov. | 1151:inocPosByCloneCepec_2004 | B   18.2282     0.8796 1781.9284  20.723  < 2e-16 ***
## day:codeP. theobromicola sp. nov. | 1205:inocPosByCloneCepec_2004 | B   15.1919     0.8796 1781.9284  17.271  < 2e-16 ***
## day:codeP. theobromicola sp. nov. | 1285:inocPosByCloneCepec_2004 | B   18.1527     0.9452 1782.0232  19.205  < 2e-16 ***
## day:codeP. palmivora | 1102:inocPosByCloneCP49 | A                      14.1116     0.9452 1782.0190  14.930  < 2e-16 ***
## day:codeP. palmivora | 1158:inocPosByCloneCP49 | A                      13.6757     0.8796 1781.9284  15.547  < 2e-16 ***
## day:codeP. palmivora | 906:inocPosByCloneCP49 | A                        3.3532     1.0304 1782.3060   3.254 0.001158 ** 
## day:codeP. palmivora | 920:inocPosByCloneCP49 | A                        7.1622     1.1464 1782.4021   6.248 5.20e-10 ***
## day:codeP. theobromicola sp. nov. | 1091:inocPosByCloneCP49 | A         19.5219     0.8796 1781.9284  22.194  < 2e-16 ***
## day:codeP. theobromicola sp. nov. | 1151:inocPosByCloneCP49 | A         17.7605     0.8796 1781.9284  20.191  < 2e-16 ***
## day:codeP. theobromicola sp. nov. | 1205:inocPosByCloneCP49 | A         16.8678     0.8796 1781.9284  19.176  < 2e-16 ***
## day:codeP. theobromicola sp. nov. | 1285:inocPosByCloneCP49 | A         18.5127     0.8796 1781.9284  21.046  < 2e-16 ***
## day:codeP. palmivora | 1102:inocPosByCloneCP49 | B                      13.2861     0.8796 1781.9284  15.104  < 2e-16 ***
## day:codeP. palmivora | 1158:inocPosByCloneCP49 | B                      11.1404     0.8796 1781.9284  12.665  < 2e-16 ***
## day:codeP. palmivora | 906:inocPosByCloneCP49 | B                        3.6358     1.3171 1782.5767   2.761 0.005829 ** 
## day:codeP. palmivora | 920:inocPosByCloneCP49 | B                        7.7687     1.1443 1782.0394   6.789 1.53e-11 ***
## day:codeP. theobromicola sp. nov. | 1091:inocPosByCloneCP49 | B         22.1189     0.8796 1781.9284  25.146  < 2e-16 ***
## day:codeP. theobromicola sp. nov. | 1151:inocPosByCloneCP49 | B         17.4514     0.8796 1781.9284  19.840  < 2e-16 ***
## day:codeP. theobromicola sp. nov. | 1205:inocPosByCloneCP49 | B         20.7602     0.9452 1782.0190  21.965  < 2e-16 ***
## day:codeP. theobromicola sp. nov. | 1285:inocPosByCloneCP49 | B         16.8036     0.8796 1781.9284  19.103  < 2e-16 ***
## day:codeP. palmivora | 1102:inocPosByClonePS1319 | A                    12.4784     0.9452 1782.0190  13.202  < 2e-16 ***
## day:codeP. palmivora | 1158:inocPosByClonePS1319 | A                    12.9891     0.8796 1781.9284  14.767  < 2e-16 ***
## day:codeP. palmivora | 906:inocPosByClonePS1319 | A                      6.5933     1.3171 1782.5771   5.006 6.11e-07 ***
## day:codeP. palmivora | 920:inocPosByClonePS1319 | A                     10.6053     1.1443 1782.0394   9.268  < 2e-16 ***
## day:codeP. theobromicola sp. nov. | 1091:inocPosByClonePS1319 | A       17.8211     0.8796 1781.9284  20.260  < 2e-16 ***
## day:codeP. theobromicola sp. nov. | 1151:inocPosByClonePS1319 | A       17.4106     0.8796 1781.9284  19.793  < 2e-16 ***
## day:codeP. theobromicola sp. nov. | 1205:inocPosByClonePS1319 | A       19.3636     0.8796 1781.9284  22.014  < 2e-16 ***
## day:codeP. theobromicola sp. nov. | 1285:inocPosByClonePS1319 | A       15.5903     0.9452 1782.0190  16.495  < 2e-16 ***
## day:codeP. palmivora | 1102:inocPosByClonePS1319 | B                    14.8251     1.0297 1782.0784  14.397  < 2e-16 ***
## day:codeP. palmivora | 1158:inocPosByClonePS1319 | B                    11.6434     0.8796 1781.9284  13.237  < 2e-16 ***
## day:codeP. palmivora | 906:inocPosByClonePS1319 | B                      2.4486     1.6014 1782.2957   1.529 0.126426    
## day:codeP. palmivora | 920:inocPosByClonePS1319 | B                      6.0009     0.9452 1782.0232   6.349 2.74e-10 ***
## day:codeP. theobromicola sp. nov. | 1091:inocPosByClonePS1319 | B       20.3003     0.8796 1781.9284  23.078  < 2e-16 ***
## day:codeP. theobromicola sp. nov. | 1151:inocPosByClonePS1319 | B       16.2454     0.8796 1781.9284  18.469  < 2e-16 ***
## day:codeP. theobromicola sp. nov. | 1205:inocPosByClonePS1319 | B       16.0171     0.8796 1781.9284  18.209  < 2e-16 ***
## day:codeP. theobromicola sp. nov. | 1285:inocPosByClonePS1319 | B       17.6773     0.8796 1781.9284  20.096  < 2e-16 ***
## ---
## Signif. codes:  0 '***' 0.001 '**' 0.01 '*' 0.05 '.' 0.1 ' ' 1
```

```
## 
## Correlation matrix not shown by default, as p = 65 > 12.
## Use print(x, correlation=TRUE)  or
##     vcov(x)        if you need it
```

### 2.2.1 How much the replicate is important to explain about the total variance?

```
## [1] "Variance: 6.4506951189 %"
```

## 2.3 Generate full model

```
## boundary (singular) fit: see ?isSingular
```

```
## Linear mixed model fit by maximum likelihood . t-tests use Satterthwaite's method ['lmerModLmerTest']
## Formula: average ~ day:code:inocPosByClone + (1 | day:code:inocPosByClone)
##    Data: dataset_clean
## 
##      AIC      BIC   logLik deviance df.resid 
##  15771.8  16141.8  -7818.9  15637.8     1783 
## 
## Scaled residuals: 
##     Min      1Q  Median      3Q     Max 
## -4.4004 -0.5033  0.1981  0.6475  3.9646 
## 
## Random effects:
##  Groups                  Name        Variance Std.Dev.
##  day:code:inocPosByClone (Intercept)   0.0     0.00   
##  Residual                            274.5    16.57   
## Number of obs: 1850, groups:  day:code:inocPosByClone, 320
## 
## Fixed effects:
##                                                                        Estimate Std. Error        df t value Pr(>|t|)    
## (Intercept)                                                              1.4054     0.9034 1850.0000   1.556  0.11994    
## day:codeP. palmivora | 1102:inocPosByCloneCCN51 | A                     13.8161     0.9447 1850.0000  14.624  < 2e-16 ***
## day:codeP. palmivora | 1158:inocPosByCloneCCN51 | A                     15.2684     1.0290 1850.0000  14.838  < 2e-16 ***
## day:codeP. palmivora | 906:inocPosByCloneCCN51 | A                       7.3029     1.3132 1850.0000   5.561 3.07e-08 ***
## day:codeP. palmivora | 920:inocPosByCloneCCN51 | A                      10.5910     1.0290 1850.0000  10.292  < 2e-16 ***
## day:codeP. theobromicola sp. nov. | 1091:inocPosByCloneCCN51 | A        19.8574     0.8796 1850.0000  22.575  < 2e-16 ***
## day:codeP. theobromicola sp. nov. | 1151:inocPosByCloneCCN51 | A        17.6055     0.8796 1850.0000  20.015  < 2e-16 ***
## day:codeP. theobromicola sp. nov. | 1205:inocPosByCloneCCN51 | A        15.2928     0.9447 1850.0000  16.187  < 2e-16 ***
## day:codeP. theobromicola sp. nov. | 1285:inocPosByCloneCCN51 | A        16.4365     0.9447 1850.0000  17.398  < 2e-16 ***
## day:codeP. palmivora | 1102:inocPosByCloneCCN51 | B                     16.2146     1.0290 1850.0000  15.757  < 2e-16 ***
## day:codeP. palmivora | 1158:inocPosByCloneCCN51 | B                     13.7860     0.8796 1850.0000  15.673  < 2e-16 ***
## day:codeP. palmivora | 906:inocPosByCloneCCN51 | B                       4.0925     1.3132 1850.0000   3.117  0.00186 ** 
## day:codeP. palmivora | 920:inocPosByCloneCCN51 | B                       8.9168     1.0290 1850.0000   8.665  < 2e-16 ***
## day:codeP. theobromicola sp. nov. | 1091:inocPosByCloneCCN51 | B        19.9015     0.9447 1850.0000  21.065  < 2e-16 ***
## day:codeP. theobromicola sp. nov. | 1151:inocPosByCloneCCN51 | B        18.8878     0.9447 1850.0000  19.992  < 2e-16 ***
## day:codeP. theobromicola sp. nov. | 1205:inocPosByCloneCCN51 | B        15.5849     0.8796 1850.0000  17.718  < 2e-16 ***
## day:codeP. theobromicola sp. nov. | 1285:inocPosByCloneCCN51 | B        18.5534     0.8796 1850.0000  21.093  < 2e-16 ***
## day:codeP. palmivora | 1102:inocPosByCloneCepec_2004 | A                13.3107     1.0290 1850.0000  12.935  < 2e-16 ***
## day:codeP. palmivora | 1158:inocPosByCloneCepec_2004 | A                15.4467     1.1439 1850.0000  13.504  < 2e-16 ***
## day:codeP. palmivora | 906:inocPosByCloneCepec_2004 | A                  1.3976     2.2476 1850.0000   0.622  0.53414    
## day:codeP. palmivora | 920:inocPosByCloneCepec_2004 | A                  4.6773     0.9447 1850.0000   4.951 8.06e-07 ***
## day:codeP. theobromicola sp. nov. | 1091:inocPosByCloneCepec_2004 | A   16.8115     1.0290 1850.0000  16.337  < 2e-16 ***
## day:codeP. theobromicola sp. nov. | 1151:inocPosByCloneCepec_2004 | A   19.1525     0.8796 1850.0000  21.774  < 2e-16 ***
## day:codeP. theobromicola sp. nov. | 1205:inocPosByCloneCepec_2004 | A   19.1837     0.8796 1850.0000  21.809  < 2e-16 ***
## day:codeP. theobromicola sp. nov. | 1285:inocPosByCloneCepec_2004 | A   18.0445     0.8796 1850.0000  20.514  < 2e-16 ***
## day:codeP. palmivora | 1102:inocPosByCloneCepec_2004 | B                12.6367     0.9447 1850.0000  13.376  < 2e-16 ***
## day:codeP. palmivora | 1158:inocPosByCloneCepec_2004 | B                13.2205     0.9447 1850.0000  13.994  < 2e-16 ***
## day:codeP. palmivora | 906:inocPosByCloneCepec_2004 | B                  9.9946     2.2476 1850.0000   4.447 9.24e-06 ***
## day:codeP. palmivora | 920:inocPosByCloneCepec_2004 | B                  9.8521     1.0290 1850.0000   9.574  < 2e-16 ***
## day:codeP. theobromicola sp. nov. | 1091:inocPosByCloneCepec_2004 | B   17.1175     0.9447 1850.0000  18.119  < 2e-16 ***
## day:codeP. theobromicola sp. nov. | 1151:inocPosByCloneCepec_2004 | B   18.2172     0.8796 1850.0000  20.711  < 2e-16 ***
## day:codeP. theobromicola sp. nov. | 1205:inocPosByCloneCepec_2004 | B   15.1809     0.8796 1850.0000  17.259  < 2e-16 ***
## day:codeP. theobromicola sp. nov. | 1285:inocPosByCloneCepec_2004 | B   18.2122     0.9447 1850.0000  19.277  < 2e-16 ***
## day:codeP. palmivora | 1102:inocPosByCloneCP49 | A                      14.1740     0.9447 1850.0000  15.003  < 2e-16 ***
## day:codeP. palmivora | 1158:inocPosByCloneCP49 | A                      13.6647     0.8796 1850.0000  15.535  < 2e-16 ***
## day:codeP. palmivora | 906:inocPosByCloneCP49 | A                        2.9710     1.0290 1850.0000   2.887  0.00393 ** 
## day:codeP. palmivora | 920:inocPosByCloneCP49 | A                        7.3951     1.1439 1850.0000   6.465 1.29e-10 ***
## day:codeP. theobromicola sp. nov. | 1091:inocPosByCloneCP49 | A         19.5109     0.8796 1850.0000  22.181  < 2e-16 ***
## day:codeP. theobromicola sp. nov. | 1151:inocPosByCloneCP49 | A         17.7495     0.8796 1850.0000  20.179  < 2e-16 ***
## day:codeP. theobromicola sp. nov. | 1205:inocPosByCloneCP49 | A         16.8568     0.8796 1850.0000  19.164  < 2e-16 ***
## day:codeP. theobromicola sp. nov. | 1285:inocPosByCloneCP49 | A         18.5017     0.8796 1850.0000  21.034  < 2e-16 ***
## day:codeP. palmivora | 1102:inocPosByCloneCP49 | B                      13.2752     0.8796 1850.0000  15.092  < 2e-16 ***
## day:codeP. palmivora | 1158:inocPosByCloneCP49 | B                      11.1294     0.8796 1850.0000  12.653  < 2e-16 ***
## day:codeP. palmivora | 906:inocPosByCloneCP49 | B                        3.1959     1.3132 1850.0000   2.434  0.01504 *  
## day:codeP. palmivora | 920:inocPosByCloneCP49 | B                        8.0059     1.1439 1850.0000   6.999 3.60e-12 ***
## day:codeP. theobromicola sp. nov. | 1091:inocPosByCloneCP49 | B         22.1079     0.8796 1850.0000  25.134  < 2e-16 ***
## day:codeP. theobromicola sp. nov. | 1151:inocPosByCloneCP49 | B         17.4405     0.8796 1850.0000  19.827  < 2e-16 ***
## day:codeP. theobromicola sp. nov. | 1205:inocPosByCloneCP49 | B         20.8226     0.9447 1850.0000  22.040  < 2e-16 ***
## day:codeP. theobromicola sp. nov. | 1285:inocPosByCloneCP49 | B         16.7926     0.8796 1850.0000  19.091  < 2e-16 ***
## day:codeP. palmivora | 1102:inocPosByClonePS1319 | A                    12.5408     0.9447 1850.0000  13.274  < 2e-16 ***
## day:codeP. palmivora | 1158:inocPosByClonePS1319 | A                    12.9782     0.8796 1850.0000  14.754  < 2e-16 ***
## day:codeP. palmivora | 906:inocPosByClonePS1319 | A                      6.1477     1.3132 1850.0000   4.682 3.05e-06 ***
## day:codeP. palmivora | 920:inocPosByClonePS1319 | A                     10.8424     1.1439 1850.0000   9.479  < 2e-16 ***
## day:codeP. theobromicola sp. nov. | 1091:inocPosByClonePS1319 | A       17.8101     0.8796 1850.0000  20.248  < 2e-16 ***
## day:codeP. theobromicola sp. nov. | 1151:inocPosByClonePS1319 | A       17.3996     0.8796 1850.0000  19.781  < 2e-16 ***
## day:codeP. theobromicola sp. nov. | 1205:inocPosByClonePS1319 | A       19.3526     0.8796 1850.0000  22.001  < 2e-16 ***
## day:codeP. theobromicola sp. nov. | 1285:inocPosByClonePS1319 | A       15.6527     0.9447 1850.0000  16.568  < 2e-16 ***
## day:codeP. palmivora | 1102:inocPosByClonePS1319 | B                    14.9869     1.0290 1850.0000  14.564  < 2e-16 ***
## day:codeP. palmivora | 1158:inocPosByClonePS1319 | B                    11.6325     0.8796 1850.0000  13.225  < 2e-16 ***
## day:codeP. palmivora | 906:inocPosByClonePS1319 | B                      2.0058     1.5988 1850.0000   1.255  0.20979    
## day:codeP. palmivora | 920:inocPosByClonePS1319 | B                      6.0605     0.9447 1850.0000   6.415 1.78e-10 ***
## day:codeP. theobromicola sp. nov. | 1091:inocPosByClonePS1319 | B       20.2893     0.8796 1850.0000  23.066  < 2e-16 ***
## day:codeP. theobromicola sp. nov. | 1151:inocPosByClonePS1319 | B       16.2344     0.8796 1850.0000  18.456  < 2e-16 ***
## day:codeP. theobromicola sp. nov. | 1205:inocPosByClonePS1319 | B       16.0061     0.8796 1850.0000  18.197  < 2e-16 ***
## day:codeP. theobromicola sp. nov. | 1285:inocPosByClonePS1319 | B       17.6663     0.8796 1850.0000  20.084  < 2e-16 ***
## ---
## Signif. codes:  0 '***' 0.001 '**' 0.01 '*' 0.05 '.' 0.1 ' ' 1
```

```
## 
## Correlation matrix not shown by default, as p = 65 > 12.
## Use print(x, correlation=TRUE)  or
##     vcov(x)        if you need it
```

```
## convergence code: 0
## boundary (singular) fit: see ?isSingular
```

### 2.3.1 Plot final model adjust

### 2.3.2 Test significance of fixed effects

### 2.3.3 Test significance of random effects

### 2.3.4 Plot a residual histogram

### 2.3.5 Plot observed vs predicted data as a scatterplot

## 2.4 Perform a second clean to dataset

Remove values from the first essay.

### 2.4.1 Plot observed reduced data as a scatterplot

## 2.5 Generate reduced model

```
## fixed-effect model matrix is rank deficient so dropping 1 column / coefficient
```

```
## Linear mixed model fit by maximum likelihood . t-tests use Satterthwaite's method ['lmerModLmerTest']
## Formula: average ~ day:code:clone + (1 | day:code:clone)
##    Data: dataset_clean2
## 
##      AIC      BIC   logLik deviance df.resid 
##   7362.9   7534.5  -3647.5   7294.9     1116 
## 
## Scaled residuals: 
##     Min      1Q  Median      3Q     Max 
## -7.6634 -0.4505  0.0445  0.4939  4.3671 
## 
## Random effects:
##  Groups         Name        Variance Std.Dev.
##  day:code:clone (Intercept)  3.196   1.788   
##  Residual                   30.859   5.555   
## Number of obs: 1150, groups:  day:code:clone, 155
## 
## Fixed effects:
##                                                          Estimate Std. Error       df t value Pr(>|t|)    
## (Intercept)                                               10.8521     0.5149 149.1406  21.074  < 2e-16 ***
## day:codeP. palmivora | 1102:cloneCCN51                    14.8449     0.3847 141.8160  38.592  < 2e-16 ***
## day:codeP. palmivora | 1158:cloneCCN51                    14.7646     0.3847 141.8160  38.384  < 2e-16 ***
## day:codeP. palmivora | 906:cloneCCN51                     -0.4640     0.5986 653.4711  -0.775 0.438588    
## day:codeP. palmivora | 920:cloneCCN51                      7.5864     0.3975 161.6772  19.087  < 2e-16 ***
## day:codeP. theobromicola sp. nov. | 1091:cloneCCN51       17.3753     0.3847 141.8160  45.171  < 2e-16 ***
## day:codeP. theobromicola sp. nov. | 1151:cloneCCN51       16.2173     0.3847 141.8160  42.160  < 2e-16 ***
## day:codeP. theobromicola sp. nov. | 1205:cloneCCN51       16.0670     0.3847 141.8160  41.770  < 2e-16 ***
## day:codeP. theobromicola sp. nov. | 1285:cloneCCN51       16.1097     0.3847 141.8160  41.881  < 2e-16 ***
## day:codeP. palmivora | 1102:cloneCepec_2004               13.6108     0.3847 141.8160  35.384  < 2e-16 ***
## day:codeP. palmivora | 1158:cloneCepec_2004               12.7353     0.3847 141.8160  33.108  < 2e-16 ***
## day:codeP. palmivora | 920:cloneCepec_2004                 4.8855     0.3847 141.8160  12.701  < 2e-16 ***
## day:codeP. theobromicola sp. nov. | 1091:cloneCepec_2004  16.2081     0.3847 141.8160  42.136  < 2e-16 ***
## day:codeP. theobromicola sp. nov. | 1151:cloneCepec_2004  16.2956     0.3847 141.8160  42.364  < 2e-16 ***
## day:codeP. theobromicola sp. nov. | 1205:cloneCepec_2004  16.6957     0.3847 141.8160  43.404  < 2e-16 ***
## day:codeP. theobromicola sp. nov. | 1285:cloneCepec_2004  16.1155     0.3847 141.8160  41.896  < 2e-16 ***
## day:codeP. palmivora | 1102:cloneCP49                     15.2151     0.3847 141.8160  39.555  < 2e-16 ***
## day:codeP. palmivora | 1158:cloneCP49                     15.8389     0.3847 141.8160  41.177  < 2e-16 ***
## day:codeP. palmivora | 906:cloneCP49                      -0.6561     0.4670 299.9738  -1.405 0.161077    
## day:codeP. palmivora | 920:cloneCP49                       5.7741     0.3975 161.6772  14.527  < 2e-16 ***
## day:codeP. theobromicola sp. nov. | 1091:cloneCP49        18.0267     0.3847 141.8160  46.864  < 2e-16 ***
## day:codeP. theobromicola sp. nov. | 1151:cloneCP49        17.5476     0.3847 141.8160  45.619  < 2e-16 ***
## day:codeP. theobromicola sp. nov. | 1205:cloneCP49        18.3890     0.3847 141.8160  47.806  < 2e-16 ***
## day:codeP. theobromicola sp. nov. | 1285:cloneCP49        18.4667     0.3847 141.8160  48.008  < 2e-16 ***
## day:codeP. palmivora | 1102:clonePS1319                   15.8531     0.3847 141.8160  41.214  < 2e-16 ***
## day:codeP. palmivora | 1158:clonePS1319                   15.4167     0.3847 141.8160  40.079  < 2e-16 ***
## day:codeP. palmivora | 906:clonePS1319                     2.3426     0.5986 653.4711   3.913 0.000101 ***
## day:codeP. palmivora | 920:clonePS1319                     7.0675     0.3847 141.8160  18.374  < 2e-16 ***
## day:codeP. theobromicola sp. nov. | 1091:clonePS1319      16.7054     0.3847 141.8160  43.429  < 2e-16 ***
## day:codeP. theobromicola sp. nov. | 1151:clonePS1319      16.2934     0.3847 141.8160  42.358  < 2e-16 ***
## day:codeP. theobromicola sp. nov. | 1205:clonePS1319      17.3136     0.3847 141.8160  45.010  < 2e-16 ***
## day:codeP. theobromicola sp. nov. | 1285:clonePS1319      16.5566     0.3847 141.8160  43.043  < 2e-16 ***
## ---
## Signif. codes:  0 '***' 0.001 '**' 0.01 '*' 0.05 '.' 0.1 ' ' 1
```

```
## 
## Correlation matrix not shown by default, as p = 32 > 12.
## Use print(x, correlation=TRUE)  or
##     vcov(x)        if you need it
```

```
## fit warnings:
## fixed-effect model matrix is rank deficient so dropping 1 column / coefficient
```

### 2.5.1 Plot final model adjust

### 2.5.2 Test significance of fixed effects

### 2.5.3 Test significance of random effects

### 2.5.4 Plot a residual histogram

## 2.6 Plot observed vs predicted with reduced data as a scatterplot

### 2.6.1 Generate pairwise comparisons

### 2.6.2 Include alpha-numeric indicators of comparisons significance

```
##  code                             clone      day lsmean   SE  df lower.CL upper.CL .group     
##  P. palmivora | 906               CP49         3   8.88 1.45 315     6.03     11.7  A         
##  P. palmivora | 906               CCN51        3   9.46 1.84 612     5.85     13.1  A         
##  P. palmivora | 906               PS1319       3  17.88 1.84 612    14.27     21.5  AB        
##  P. palmivora | 920               Cepec_2004   3  25.51 1.22 184    23.11     27.9   BC       
##  P. palmivora | 920               CP49         3  28.17 1.25 201    25.70     30.6    CD      
##  P. palmivora | 920               PS1319       3  32.05 1.22 184    29.65     34.5    CD      
##  P. palmivora | 920               CCN51        3  33.61 1.25 201    31.14     36.1     D      
##  P. palmivora | 1158              Cepec_2004   3  49.06 1.22 184    46.66     51.5      E     
##  P. palmivora | 1102              Cepec_2004   3  51.68 1.22 184    49.28     54.1      EF    
##  P. palmivora | 1158              CCN51        3  55.15 1.22 184    52.75     57.5      EFG   
##  P. palmivora | 1102              CCN51        3  55.39 1.22 184    52.99     57.8      EFG   
##  P. palmivora | 1102              CP49         3  56.50 1.22 184    54.10     58.9       FGH  
##  P. palmivora | 1158              PS1319       3  57.10 1.22 184    54.70     59.5       FGH  
##  P. palmivora | 1158              CP49         3  58.37 1.22 184    55.97     60.8       FGHI 
##  P. palmivora | 1102              PS1319       3  58.41 1.22 184    56.01     60.8       FGHI 
##  P. theobromicola sp. nov. | 1205 CCN51        3  59.05 1.22 184    56.65     61.5        GHIJ
##  P. theobromicola sp. nov. | 1285 CCN51        3  59.18 1.22 184    56.78     61.6        GHIJ
##  P. theobromicola sp. nov. | 1285 Cepec_2004   3  59.20 1.22 184    56.80     61.6        GHIJ
##  P. theobromicola sp. nov. | 1091 Cepec_2004   3  59.48 1.22 184    57.08     61.9        GHIJ
##  P. theobromicola sp. nov. | 1151 CCN51        3  59.50 1.22 184    57.10     61.9        GHIJ
##  P. theobromicola sp. nov. | 1151 PS1319       3  59.73 1.22 184    57.33     62.1        GHIJ
##  P. theobromicola sp. nov. | 1151 Cepec_2004   3  59.74 1.22 184    57.34     62.1        GHIJ
##  P. theobromicola sp. nov. | 1285 PS1319       3  60.52 1.22 184    58.12     62.9        GHIJ
##  P. theobromicola sp. nov. | 1205 Cepec_2004   3  60.94 1.22 184    58.54     63.3        GHIJ
##  P. theobromicola sp. nov. | 1091 PS1319       3  60.97 1.22 184    58.57     63.4        GHIJ
##  P. theobromicola sp. nov. | 1205 PS1319       3  62.79 1.22 184    60.39     65.2         HIJ
##  P. theobromicola sp. nov. | 1091 CCN51        3  62.98 1.22 184    60.58     65.4         HIJ
##  P. theobromicola sp. nov. | 1151 CP49         3  63.49 1.22 184    61.10     65.9         HIJ
##  P. theobromicola sp. nov. | 1091 CP49         3  64.93 1.22 184    62.53     67.3          IJ
##  P. theobromicola sp. nov. | 1205 CP49         3  66.02 1.22 184    63.62     68.4           J
##  P. theobromicola sp. nov. | 1285 CP49         3  66.25 1.22 184    63.85     68.7           J
## 
## Degrees-of-freedom method: kenward-roger 
## Confidence level used: 0.95 
## P value adjustment: tukey method for comparing a family of 31 estimates 
## significance level used: alpha = 0.01
```

### 2.6.3 Plot multiplicity comparisons

### 2.6.4 Plot estimates
