# Supplementary figures and images for: Phytophthora theobromicola sp. nov.: A New Species Causing Black Pod Disease on Cacao in Brazil
Source: Front Microbiol. 2021 Mar 15;12:537399. doi: 10.3389/fmicb.2021.537399 (PMC8015942; doi:10.3389/fmicb.2021.537399)

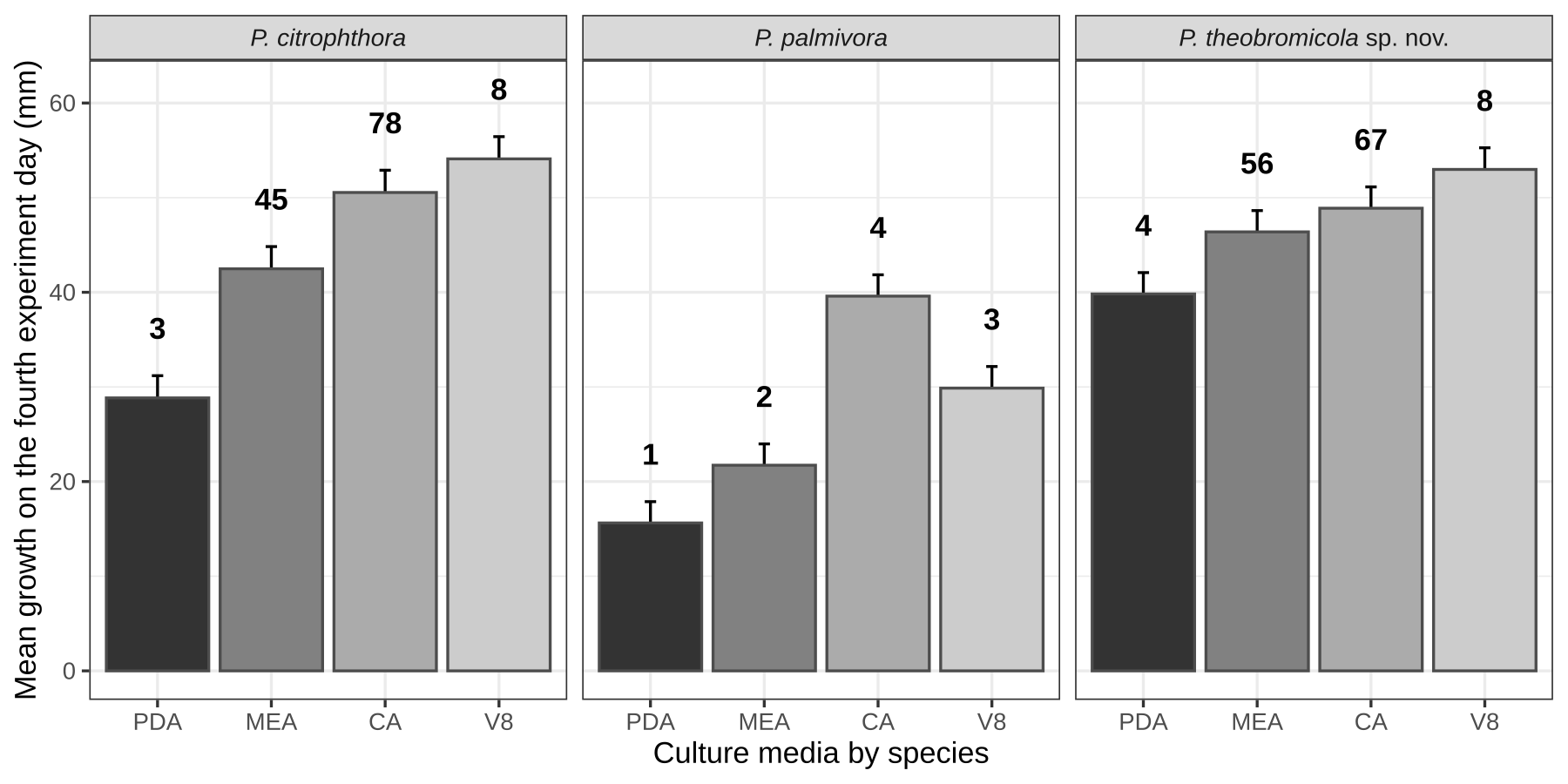

Supplement: Supplementary file 2 [file Data_Sheet_1.ZIP › essay-1/editable-figures/Barplot_observed_and_predicted_reduced_model.png]

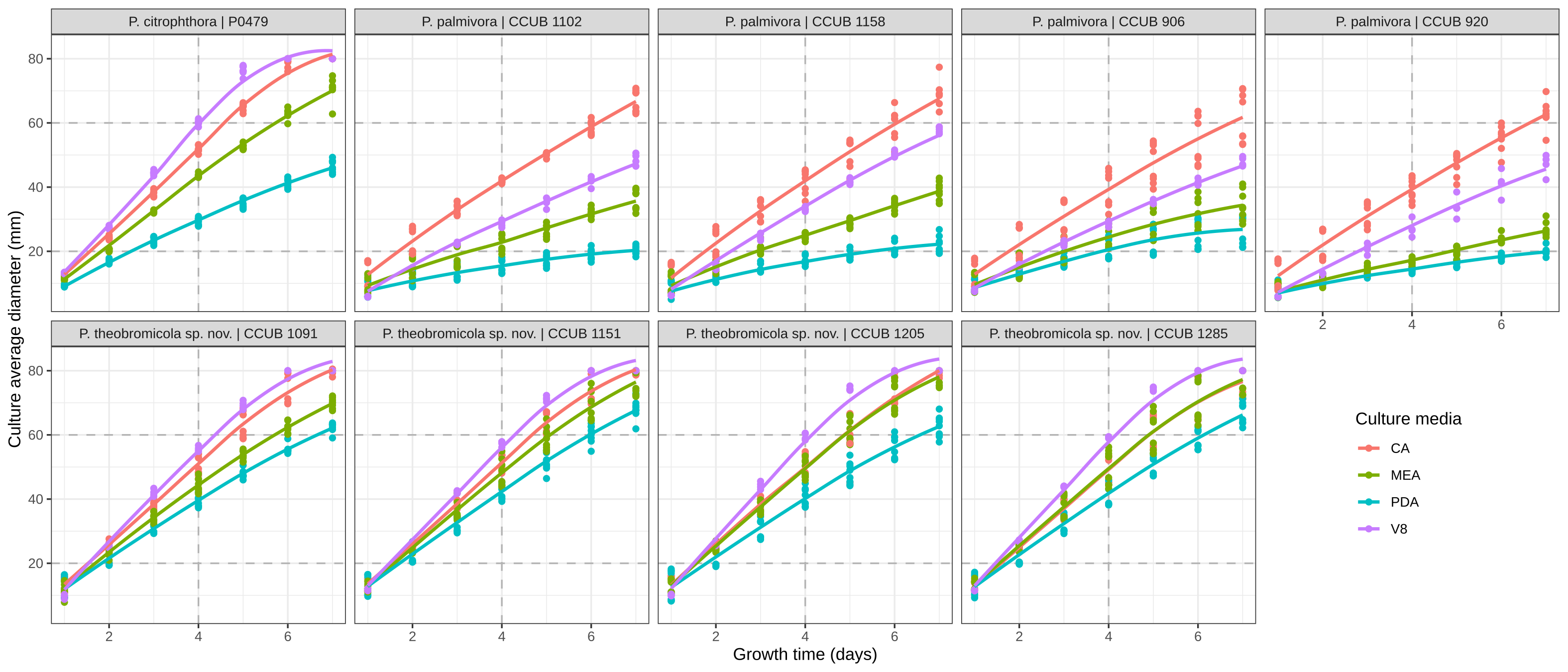

Supplement: Supplementary file 2 [file Data_Sheet_1.ZIP › essay-1/editable-figures/Scatter_observed_and_predicted.png]

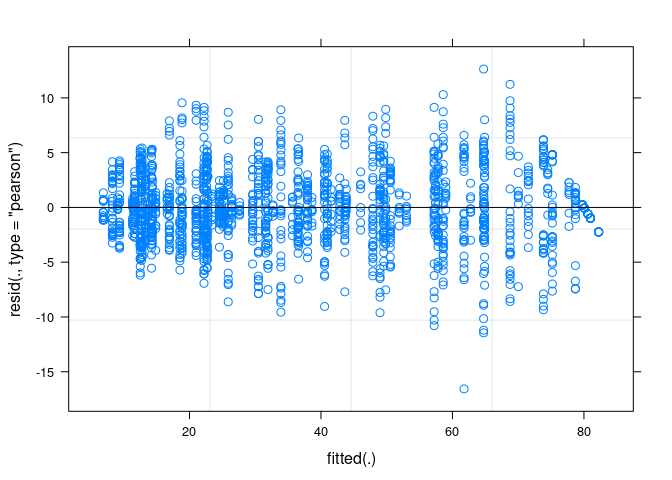

Supplement: Supplementary file 2 [file Data_Sheet_1.ZIP › essay-1/essay-1_files/figure-html/unnamed-chunk-22-1.png]

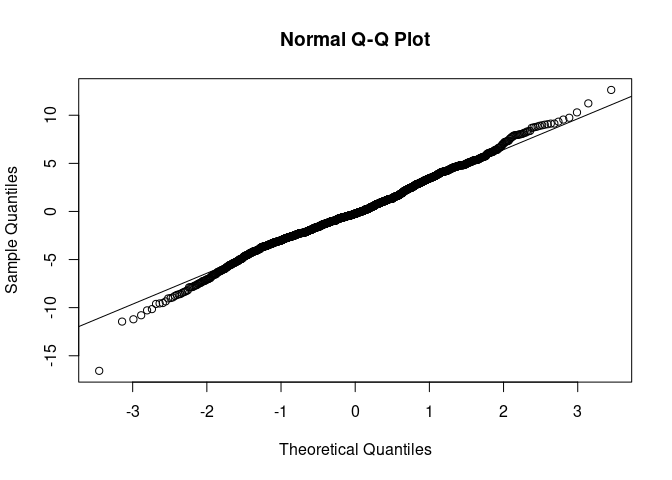

Supplement: Supplementary file 2 [file Data_Sheet_1.ZIP › essay-1/essay-1_files/figure-html/unnamed-chunk-22-2.png]

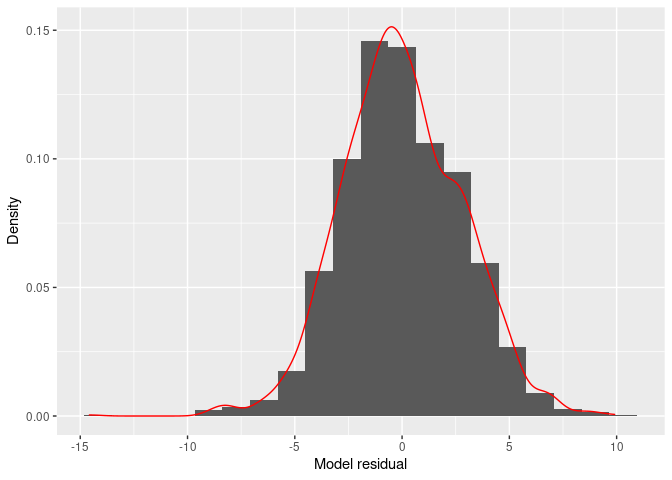

Supplement: Supplementary file 2 [file Data_Sheet_1.ZIP › essay-1/essay-1_files/figure-html/unnamed-chunk-25-1.png]

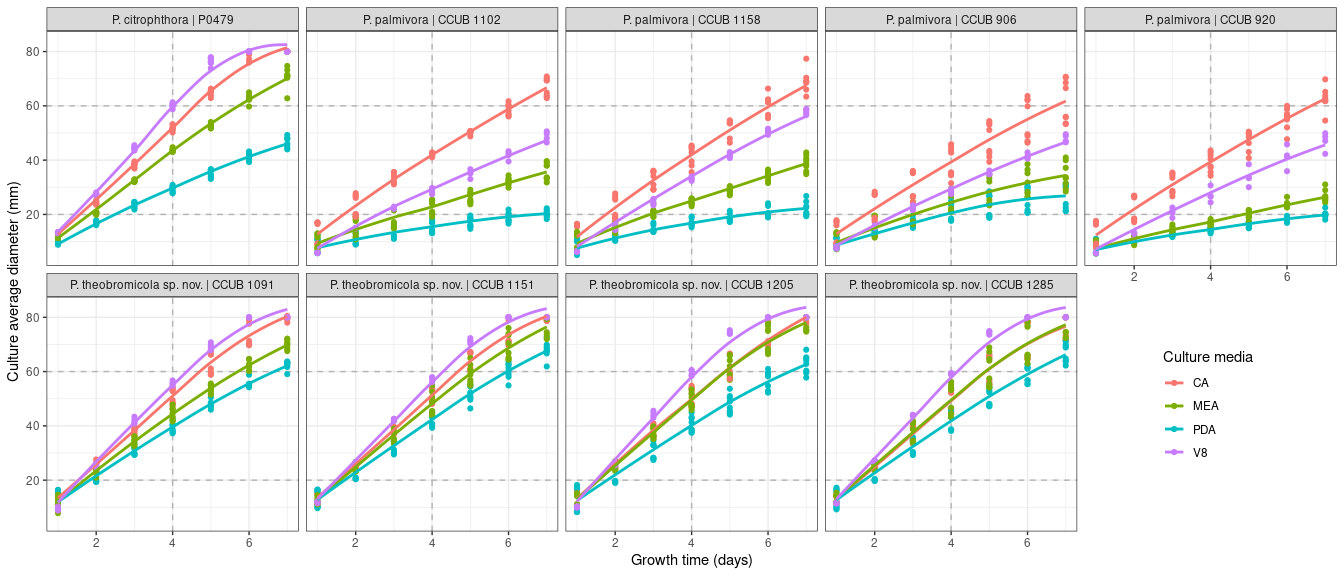

Supplement: Supplementary file 2 [file Data_Sheet_1.ZIP › essay-1/essay-1_files/figure-html/unnamed-chunk-16-1.png]

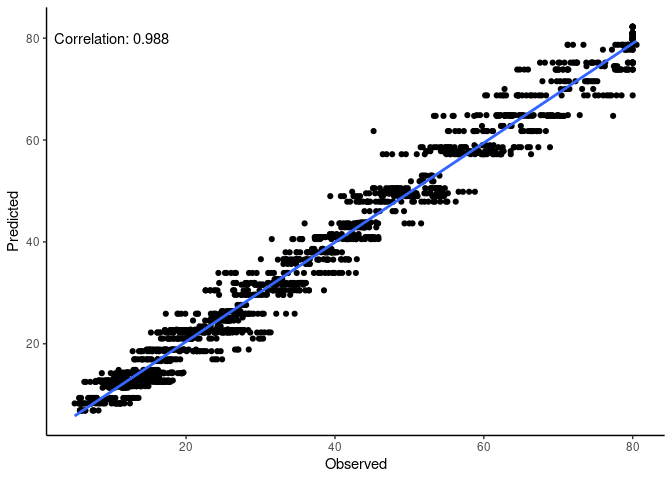

Supplement: Supplementary file 2 [file Data_Sheet_1.ZIP › essay-1/essay-1_files/figure-html/unnamed-chunk-26-1.png]

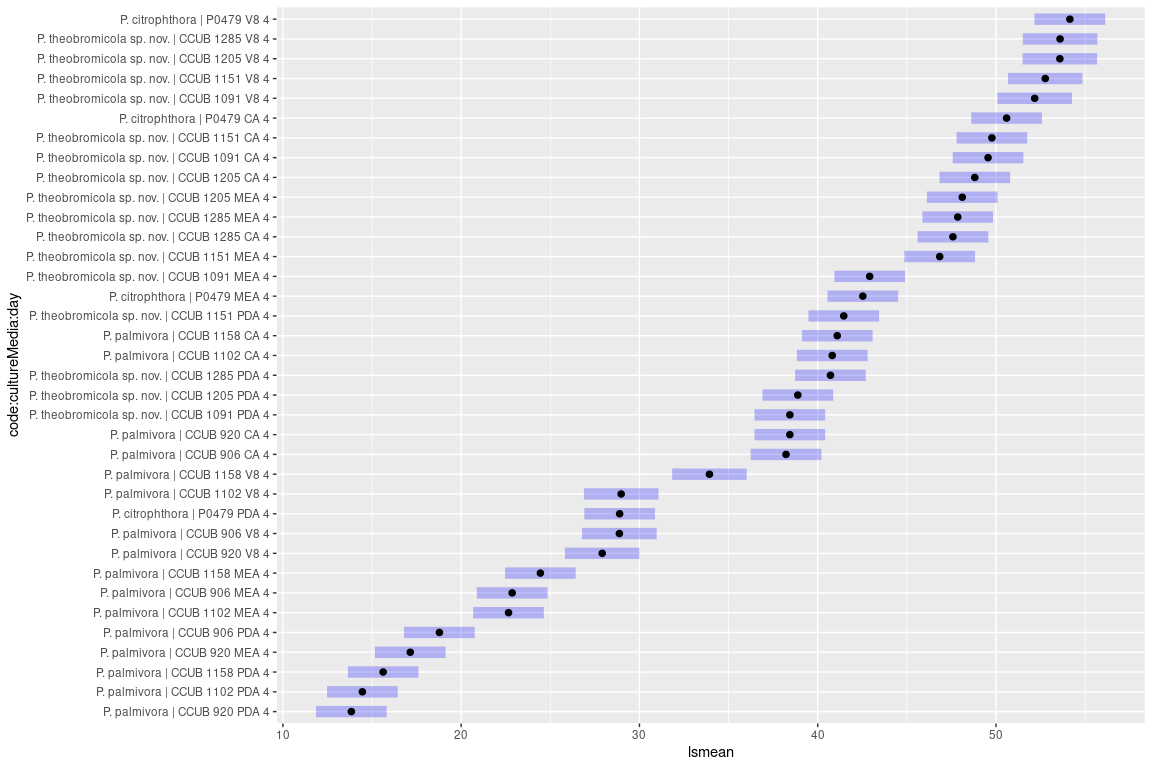

Supplement: Supplementary file 2 [file Data_Sheet_1.ZIP › essay-1/essay-1_files/figure-html/unnamed-chunk-19-1.png]

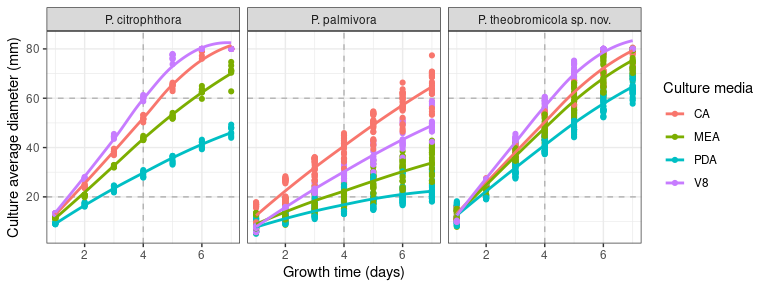

Supplement: Supplementary file 2 [file Data_Sheet_1.ZIP › essay-1/essay-1_files/figure-html/unnamed-chunk-27-1.png]

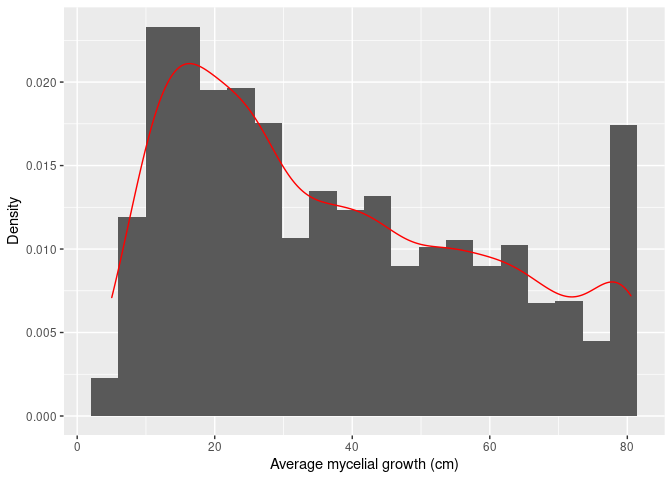

Supplement: Supplementary file 2 [file Data_Sheet_1.ZIP › essay-1/essay-1_files/figure-html/unnamed-chunk-3-1.png]

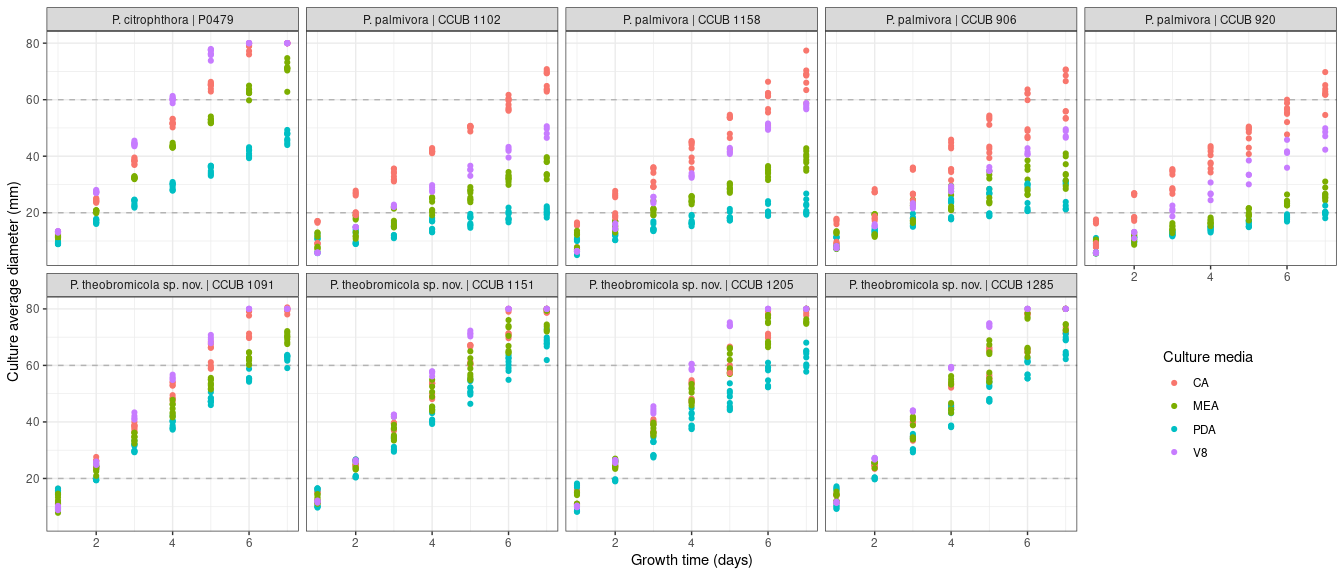

Supplement: Supplementary file 2 [file Data_Sheet_1.ZIP › essay-1/essay-1_files/figure-html/unnamed-chunk-4-1.png]

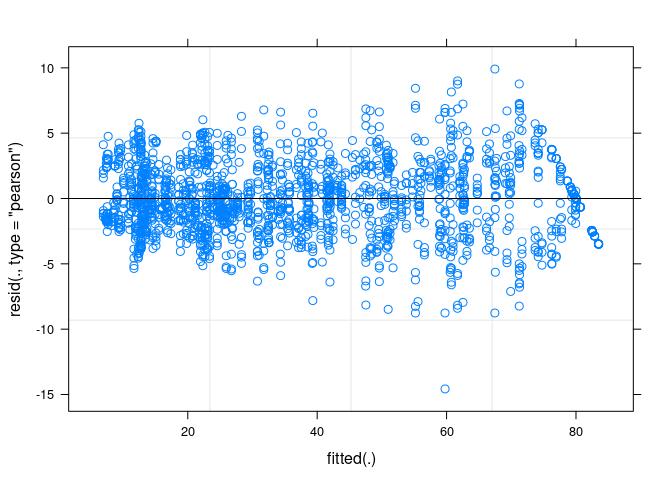

Supplement: Supplementary file 2 [file Data_Sheet_1.ZIP › essay-1/essay-1_files/figure-html/unnamed-chunk-11-1.png]

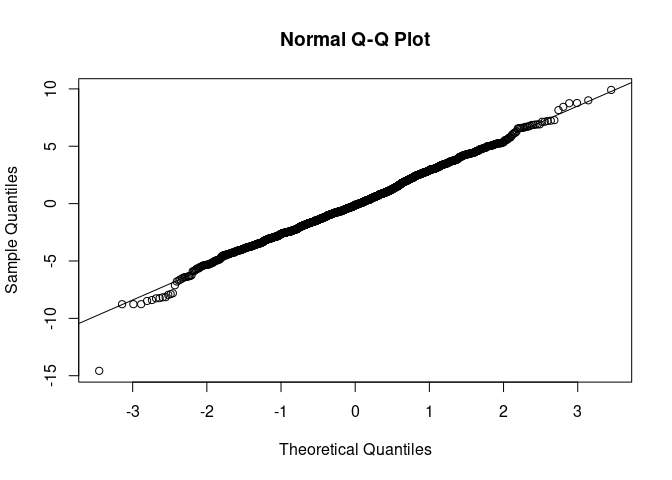

Supplement: Supplementary file 2 [file Data_Sheet_1.ZIP › essay-1/essay-1_files/figure-html/unnamed-chunk-11-2.png]

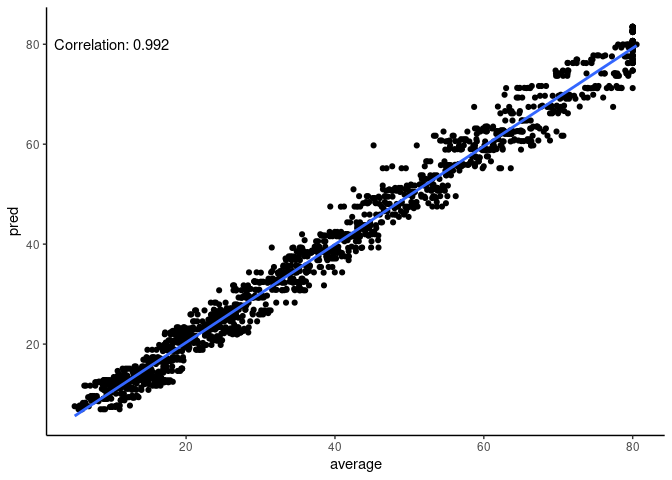

Supplement: Supplementary file 2 [file Data_Sheet_1.ZIP › essay-1/essay-1_files/figure-html/unnamed-chunk-15-1.png]

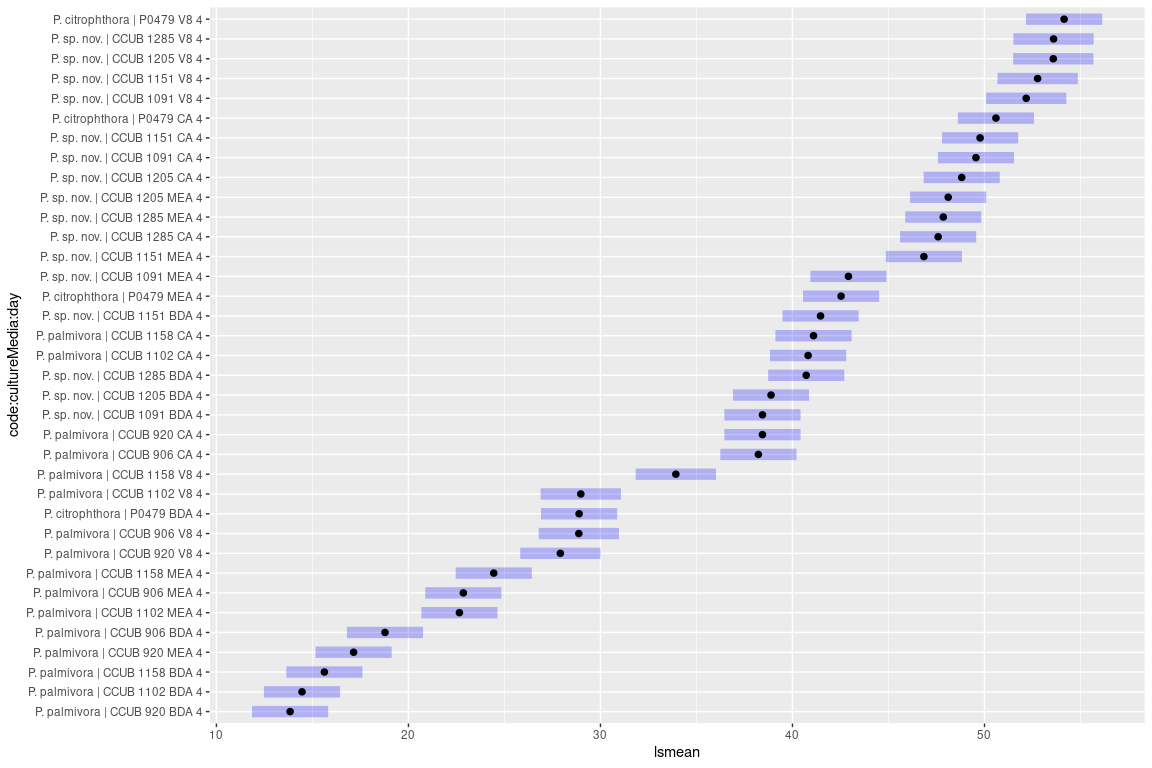

Supplement: Supplementary file 2 [file Data_Sheet_1.ZIP › essay-1/essay-1_files/figure-html/unnamed-chunk-18-1.png]

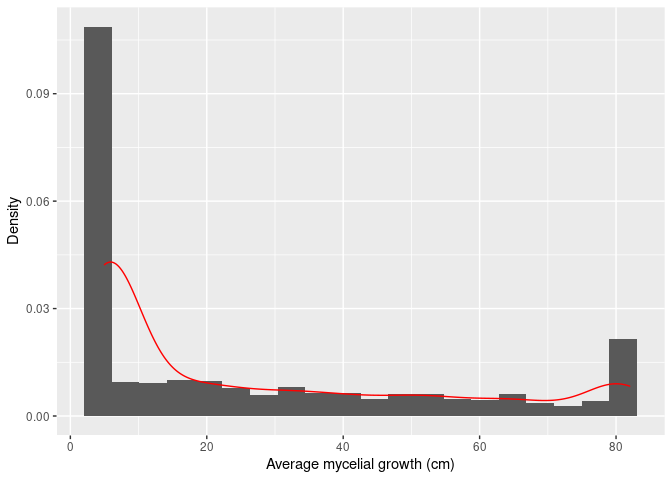

Supplement: Supplementary file 2 [file Data_Sheet_1.ZIP › essay-1/essay-1_files/figure-html/unnamed-chunk-20-1.png]

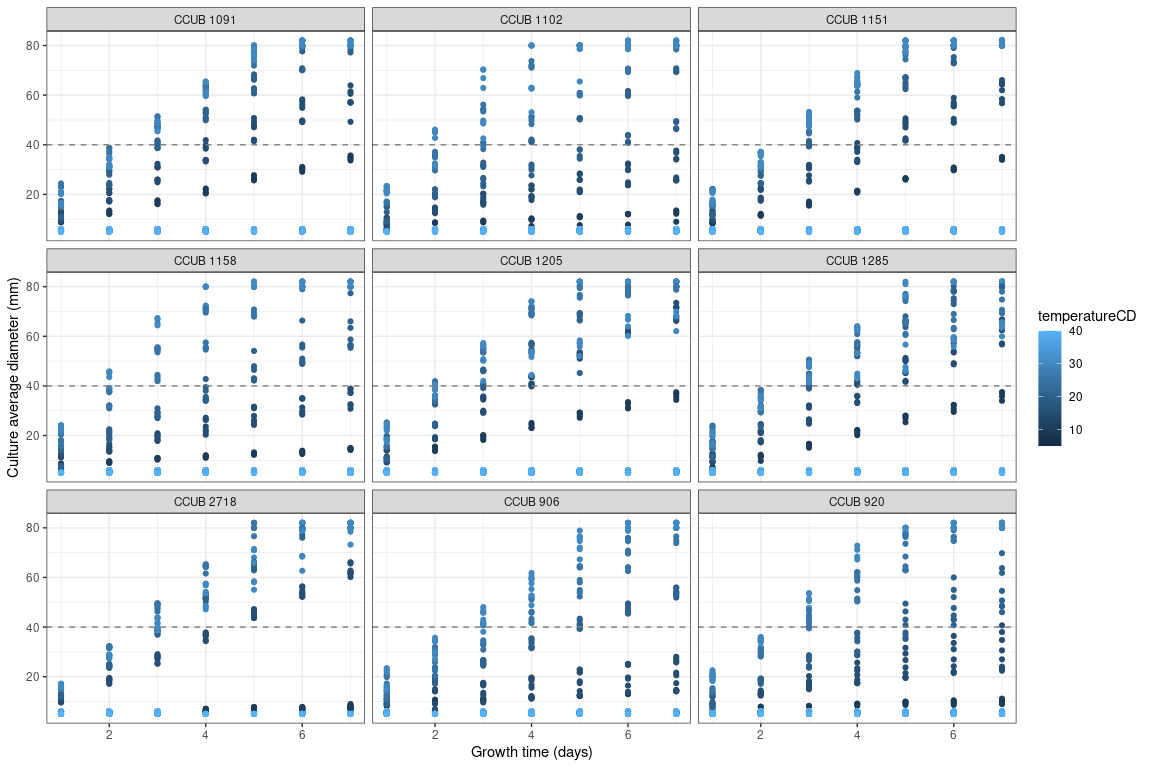

Supplement: Supplementary file 2 [file Data_Sheet_1.ZIP › essay-1/essay-1_files/figure-html/unnamed-chunk-21-1.png]

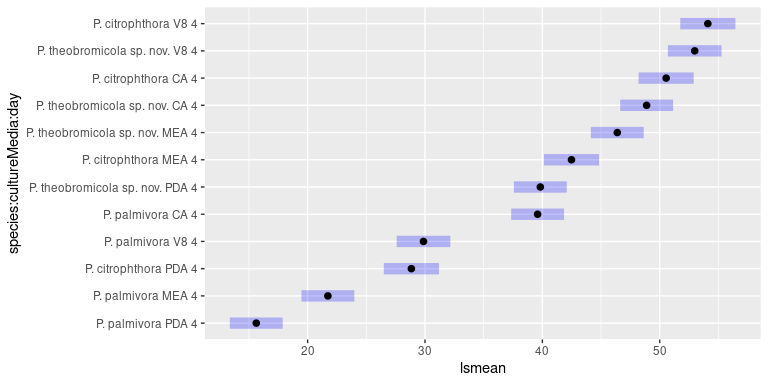

Supplement: Supplementary file 2 [file Data_Sheet_1.ZIP › essay-1/essay-1_files/figure-html/unnamed-chunk-30-1.png]

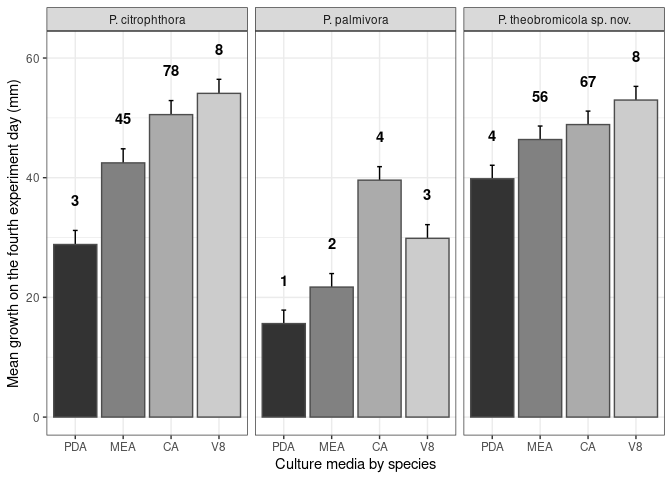

Supplement: Supplementary file 2 [file Data_Sheet_1.ZIP › essay-1/essay-1_files/figure-html/unnamed-chunk-31-1.png]

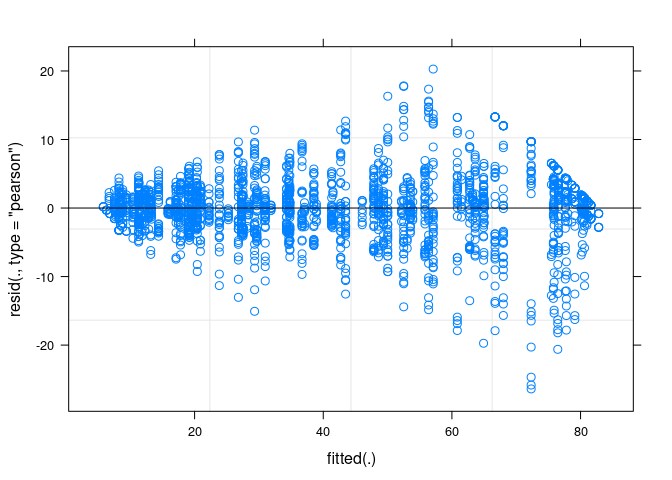

Supplement: Supplementary file 2 [file Data_Sheet_1.ZIP › essay-2/essay-2_files/figure-html/unnamed-chunk-24-1.png]

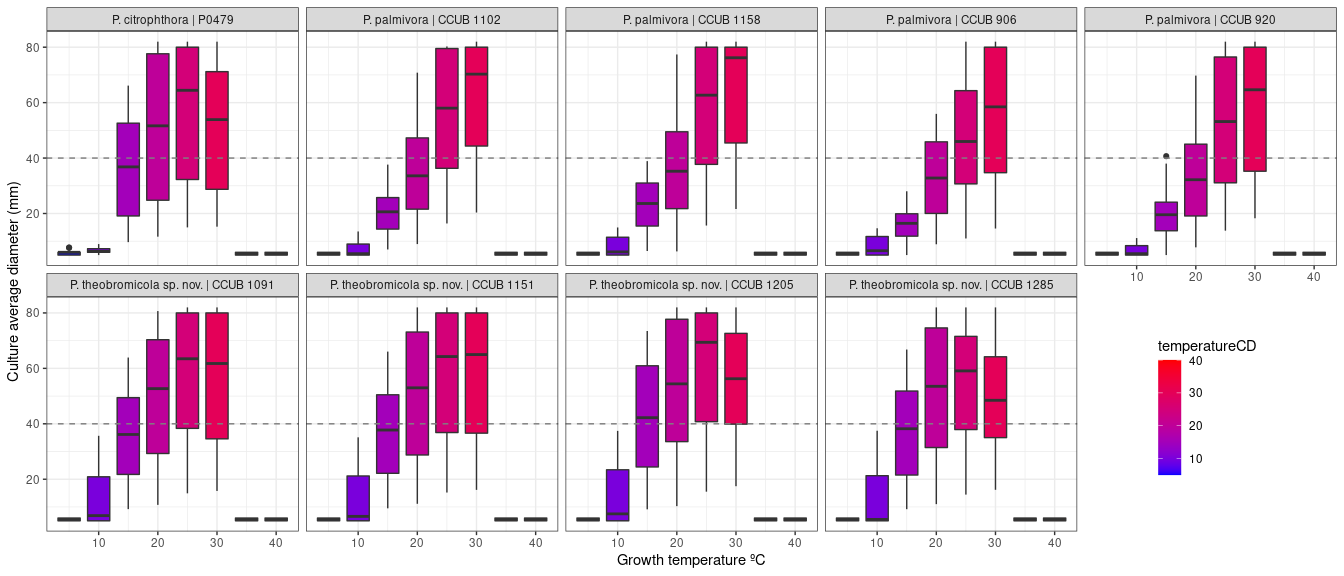

Supplement: Supplementary file 2 [file Data_Sheet_1.ZIP › essay-2/essay-2_files/figure-html/unnamed-chunk-4-1.png]

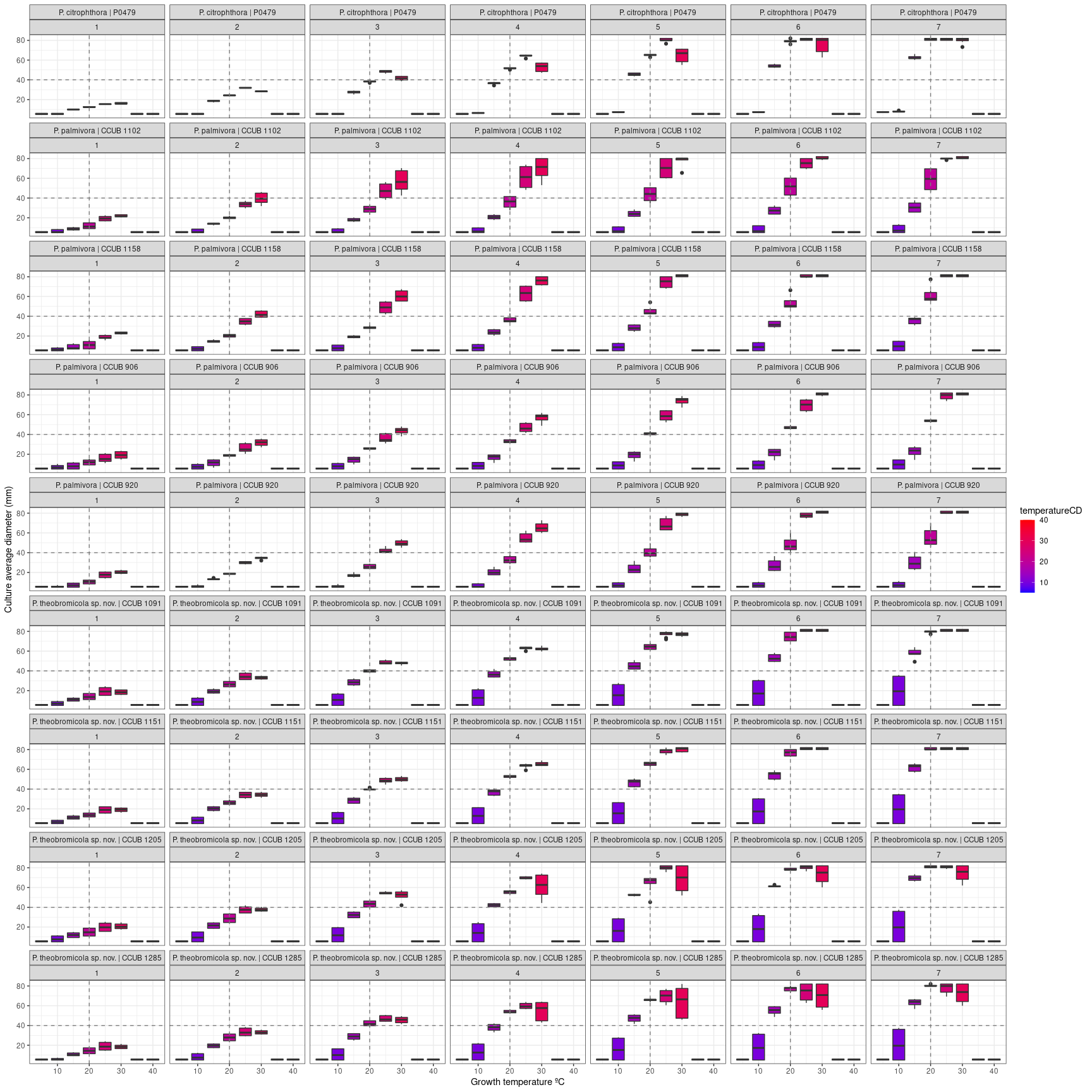

Supplement: Supplementary file 2 [file Data_Sheet_1.ZIP › essay-2/essay-2_files/figure-html/unnamed-chunk-5-1.png]

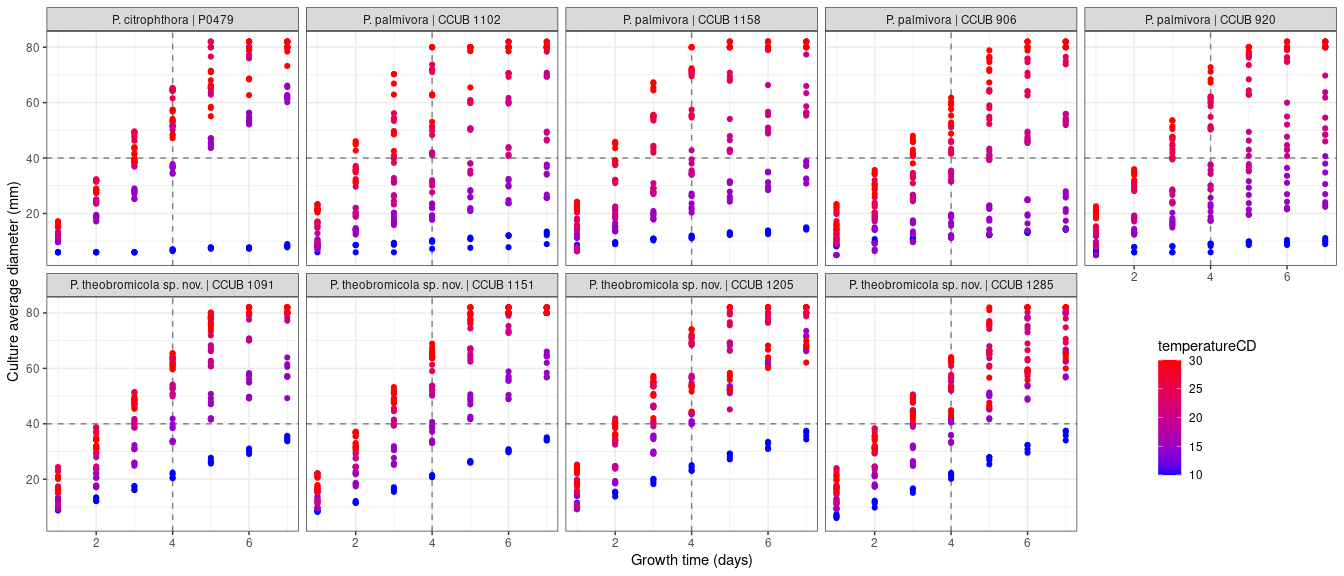

Supplement: Supplementary file 2 [file Data_Sheet_1.ZIP › essay-2/essay-2_files/figure-html/unnamed-chunk-7-1.png]

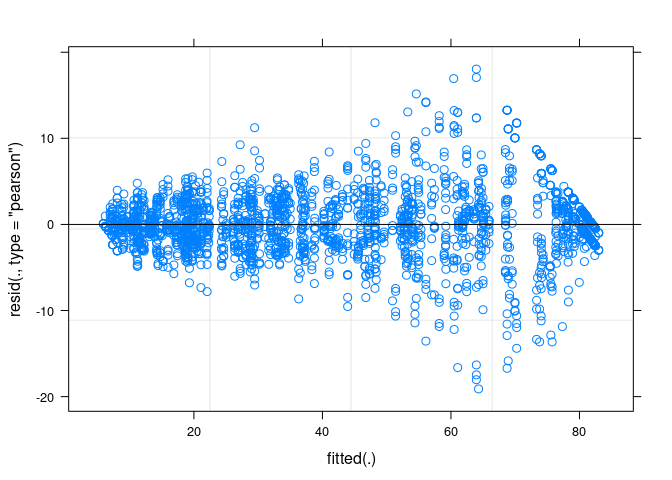

Supplement: Supplementary file 2 [file Data_Sheet_1.ZIP › essay-2/essay-2_files/figure-html/unnamed-chunk-14-1.png]

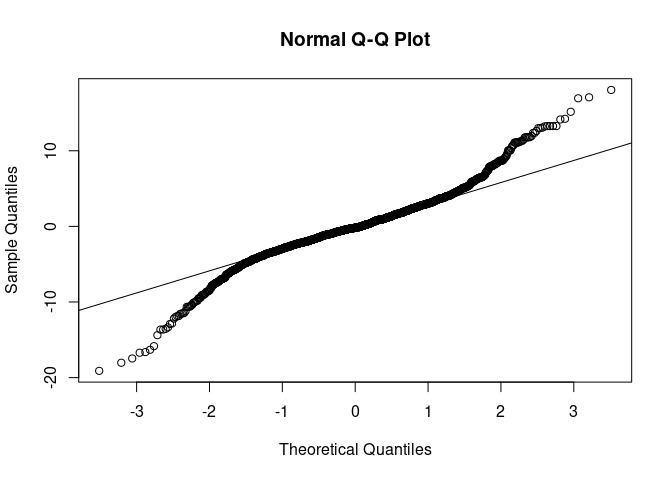

Supplement: Supplementary file 2 [file Data_Sheet_1.ZIP › essay-2/essay-2_files/figure-html/unnamed-chunk-14-2.png]

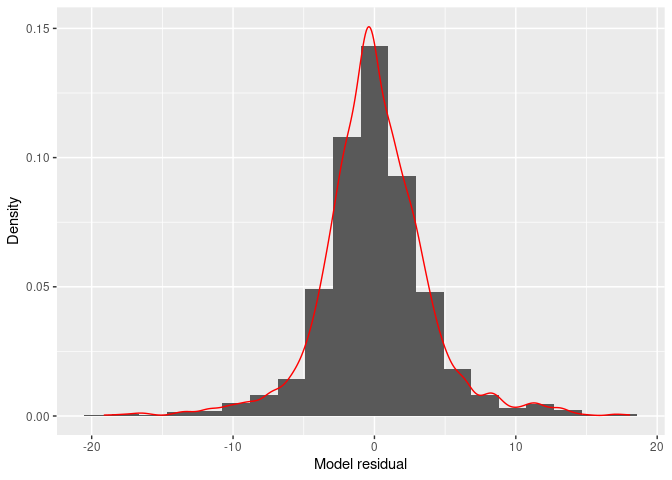

Supplement: Supplementary file 2 [file Data_Sheet_1.ZIP › essay-2/essay-2_files/figure-html/unnamed-chunk-17-1.png]

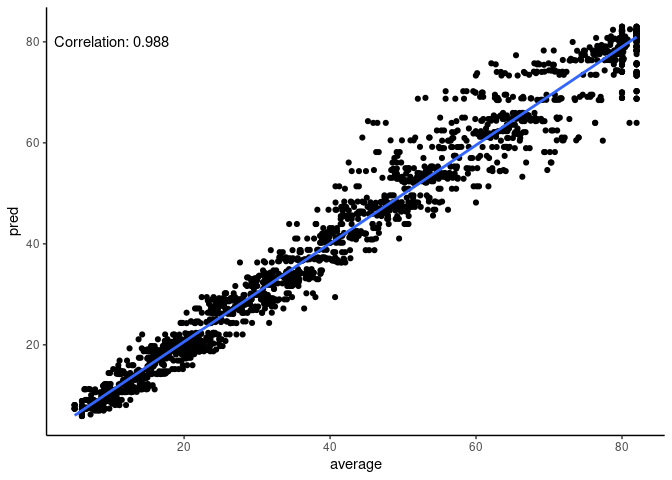

Supplement: Supplementary file 2 [file Data_Sheet_1.ZIP › essay-2/essay-2_files/figure-html/unnamed-chunk-18-1.png]

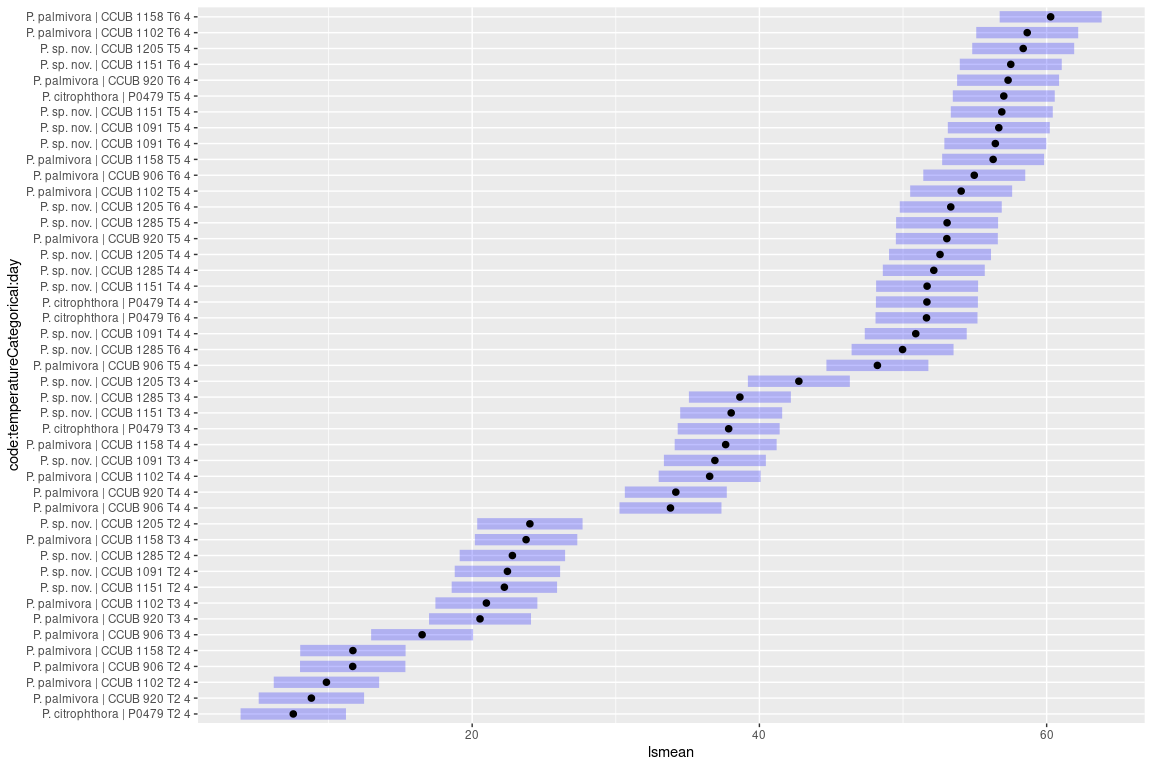

Supplement: Supplementary file 2 [file Data_Sheet_1.ZIP › essay-2/essay-2_files/figure-html/unnamed-chunk-21-1.png]

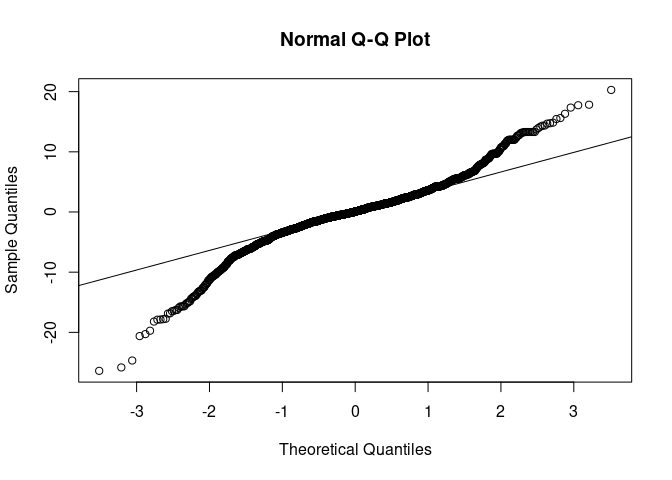

Supplement: Supplementary file 2 [file Data_Sheet_1.ZIP › essay-2/essay-2_files/figure-html/unnamed-chunk-24-2.png]

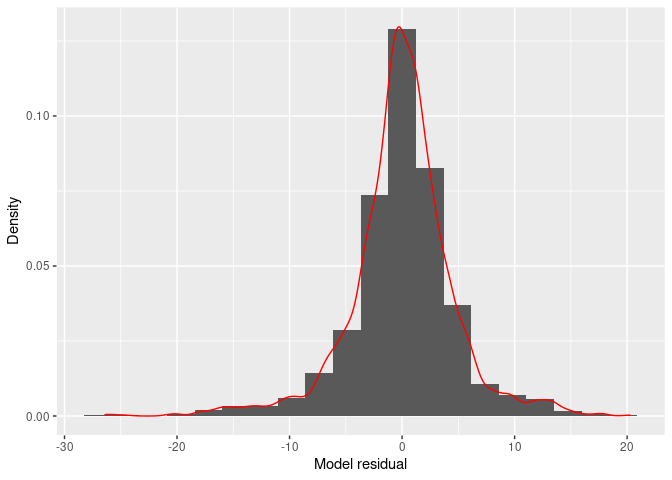

Supplement: Supplementary file 2 [file Data_Sheet_1.ZIP › essay-2/essay-2_files/figure-html/unnamed-chunk-27-1.png]

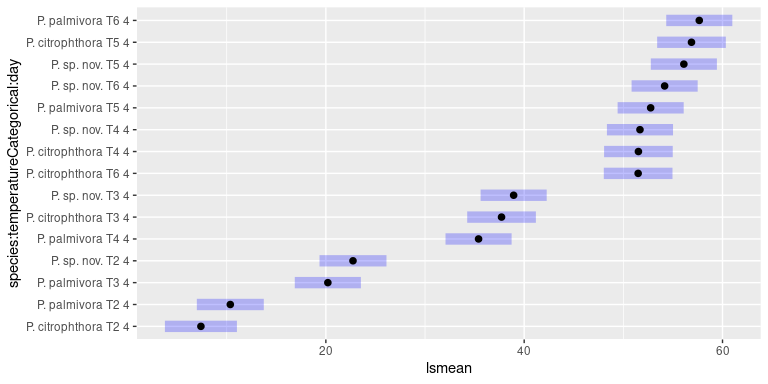

Supplement: Supplementary file 2 [file Data_Sheet_1.ZIP › essay-2/essay-2_files/figure-html/unnamed-chunk-30-1.png]

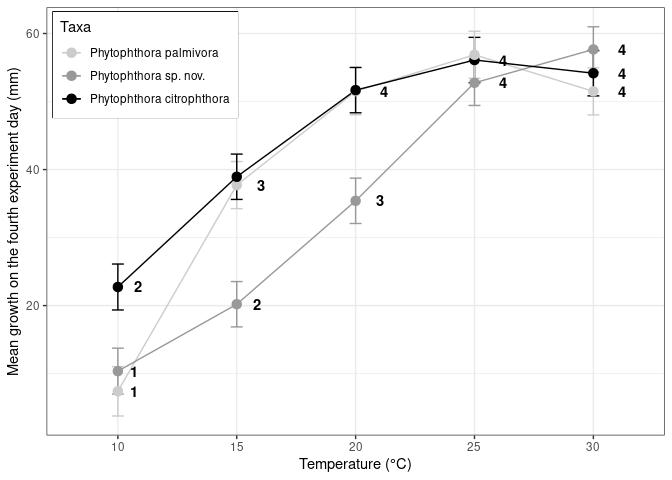

Supplement: Supplementary file 2 [file Data_Sheet_1.ZIP › essay-2/essay-2_files/figure-html/unnamed-chunk-31-1.png]

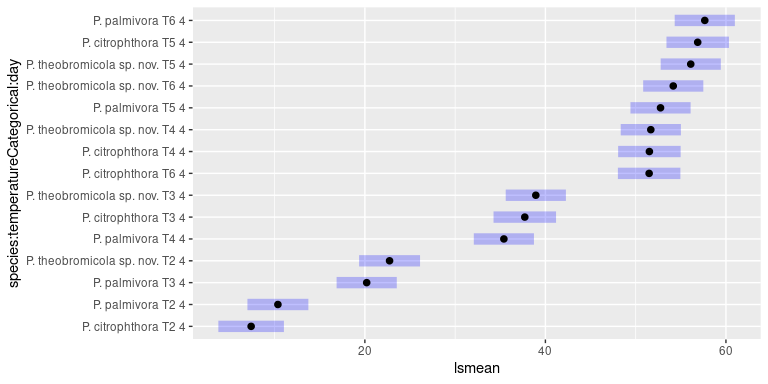

Supplement: Supplementary file 2 [file Data_Sheet_1.ZIP › essay-2/essay-2_files/figure-html/unnamed-chunk-32-1.png]

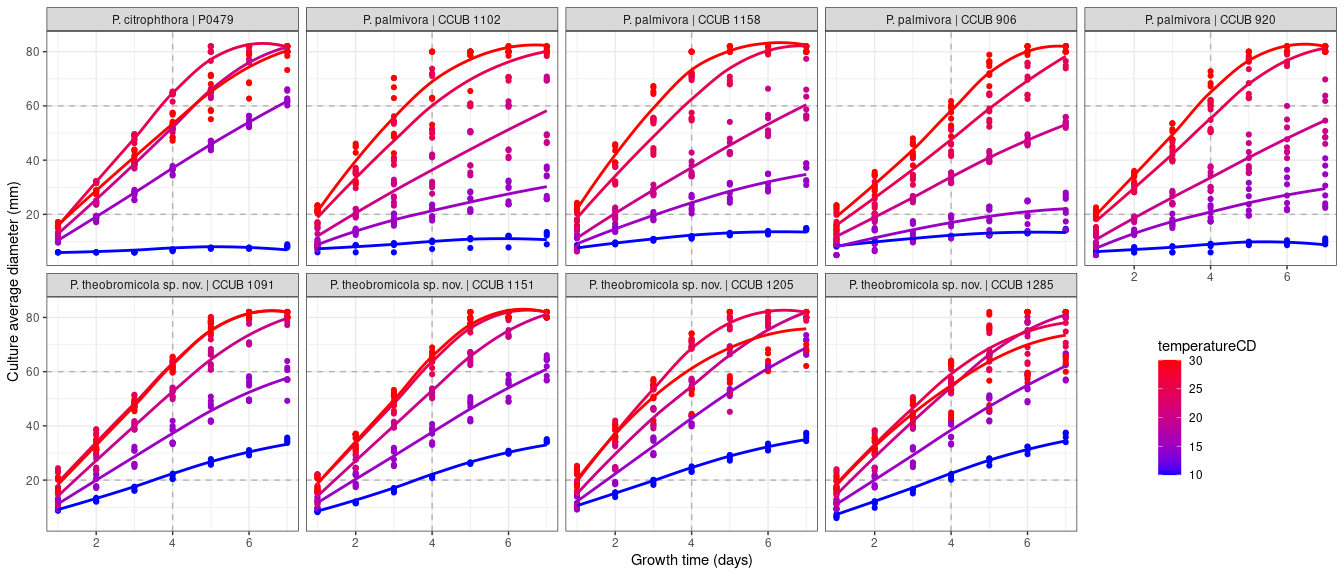

Supplement: Supplementary file 2 [file Data_Sheet_1.ZIP › essay-2/essay-2_files/figure-html/unnamed-chunk-19-1.png]

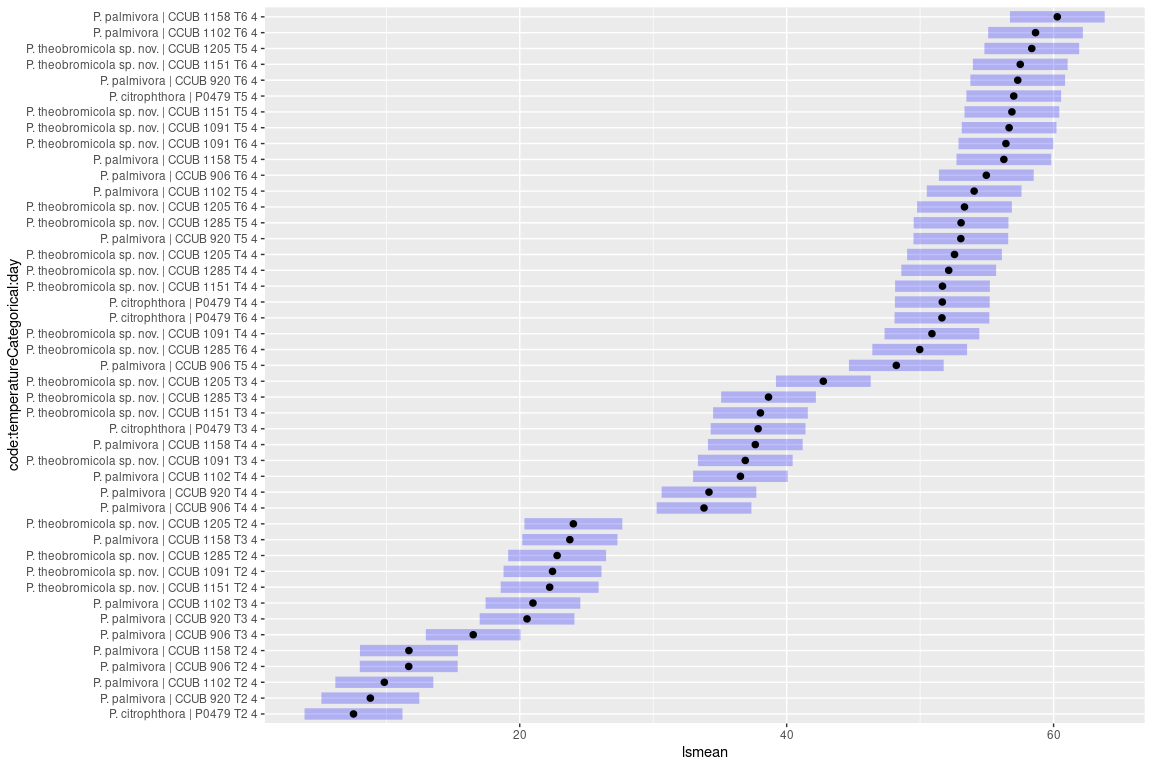

Supplement: Supplementary file 2 [file Data_Sheet_1.ZIP › essay-2/essay-2_files/figure-html/unnamed-chunk-22-1.png]

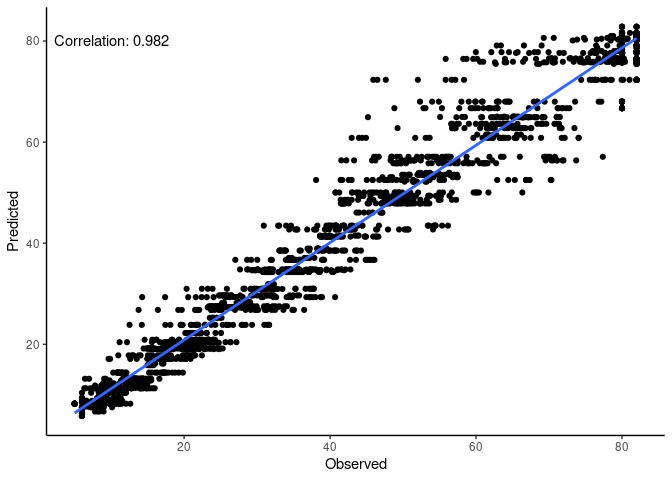

Supplement: Supplementary file 2 [file Data_Sheet_1.ZIP › essay-2/essay-2_files/figure-html/unnamed-chunk-29-1.png]

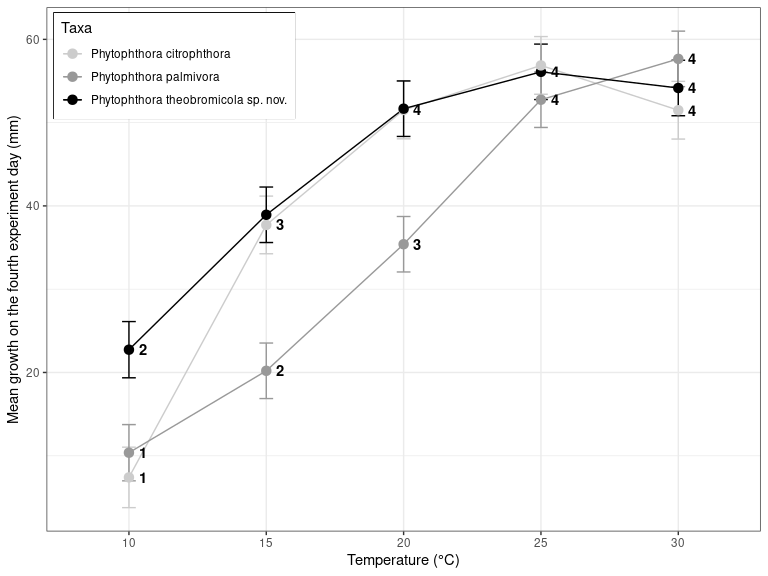

Supplement: Supplementary file 2 [file Data_Sheet_1.ZIP › essay-2/essay-2_files/figure-html/unnamed-chunk-33-1.png]

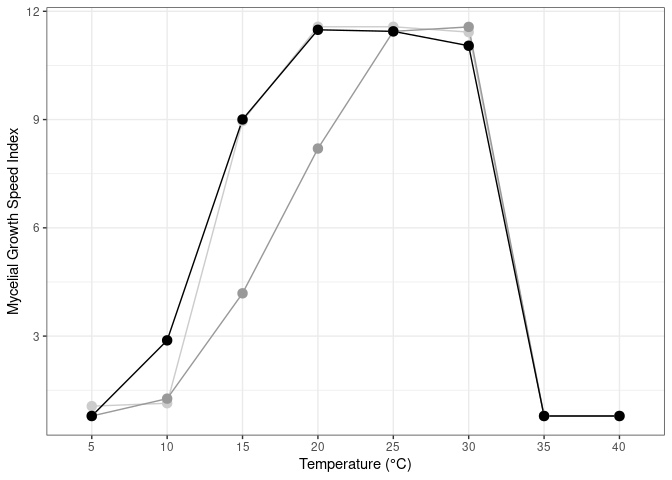

Supplement: Supplementary file 2 [file Data_Sheet_1.ZIP › essay-2/essay-2_files/figure-html/unnamed-chunk-35-1.png]

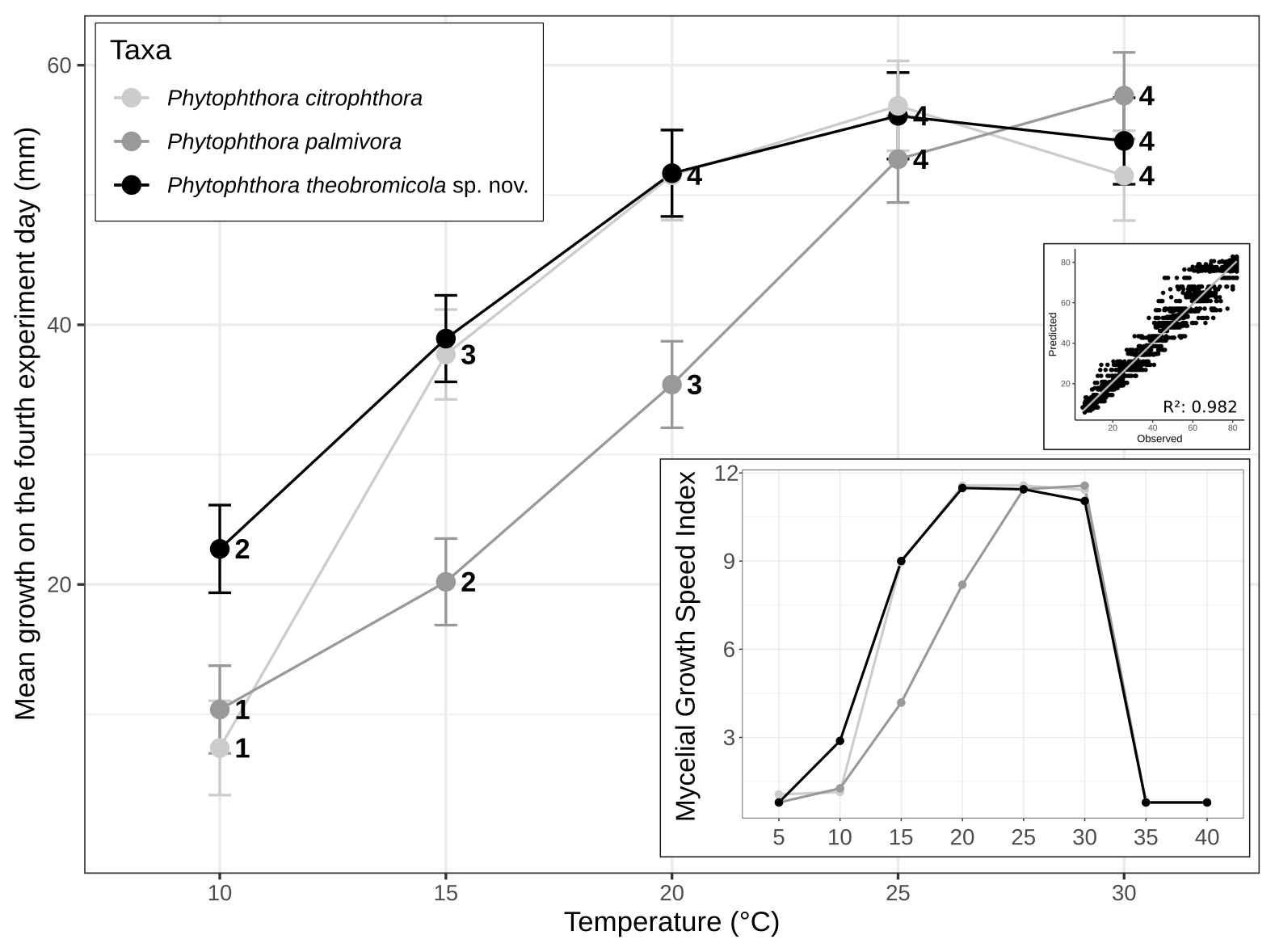

Supplement: Supplementary file 2 [file Data_Sheet_1.ZIP › essay-2/editable-figures/Scatter_predicted_reduced_model-mod.png]

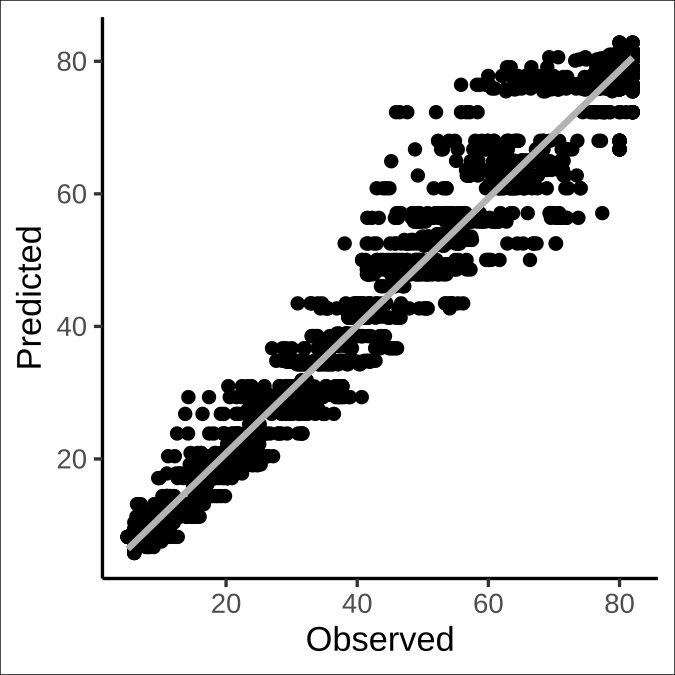

Supplement: Supplementary file 2 [file Data_Sheet_1.ZIP › essay-2/editable-figures/Adjust_residuals_reduced_model.png]

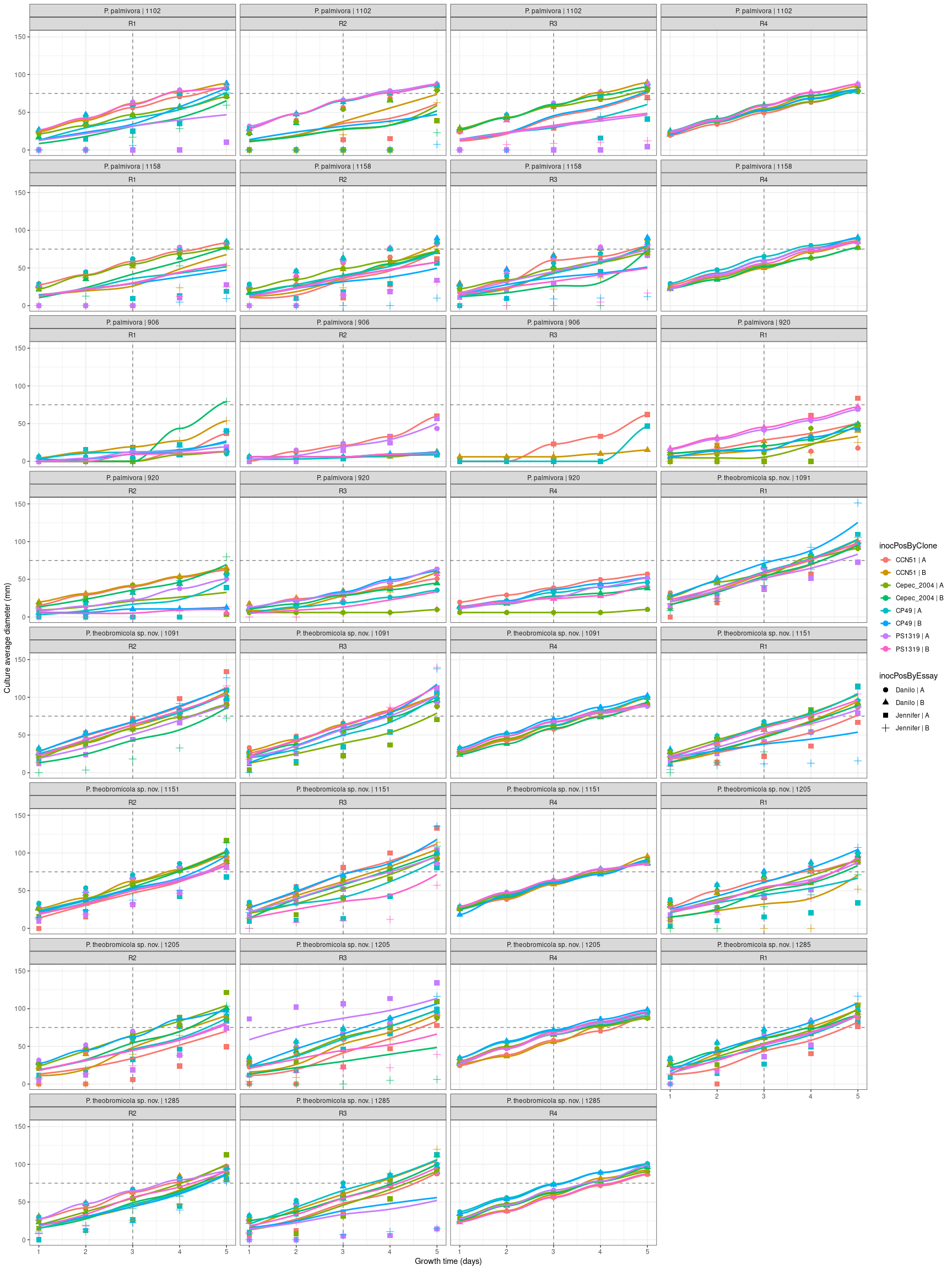

Supplement: Supplementary file 2 [file Data_Sheet_1.ZIP › essay-3/essay-3_files/figure-html/unnamed-chunk-8-1.png]

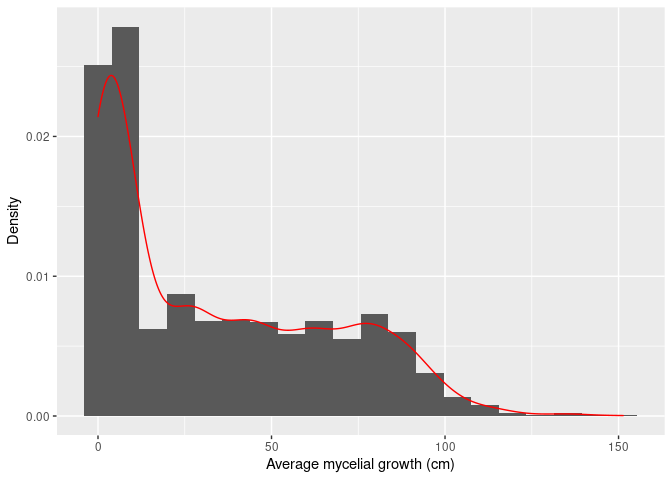

Supplement: Supplementary file 2 [file Data_Sheet_1.ZIP › essay-3/essay-3_files/figure-html/unnamed-chunk-3-1.png]

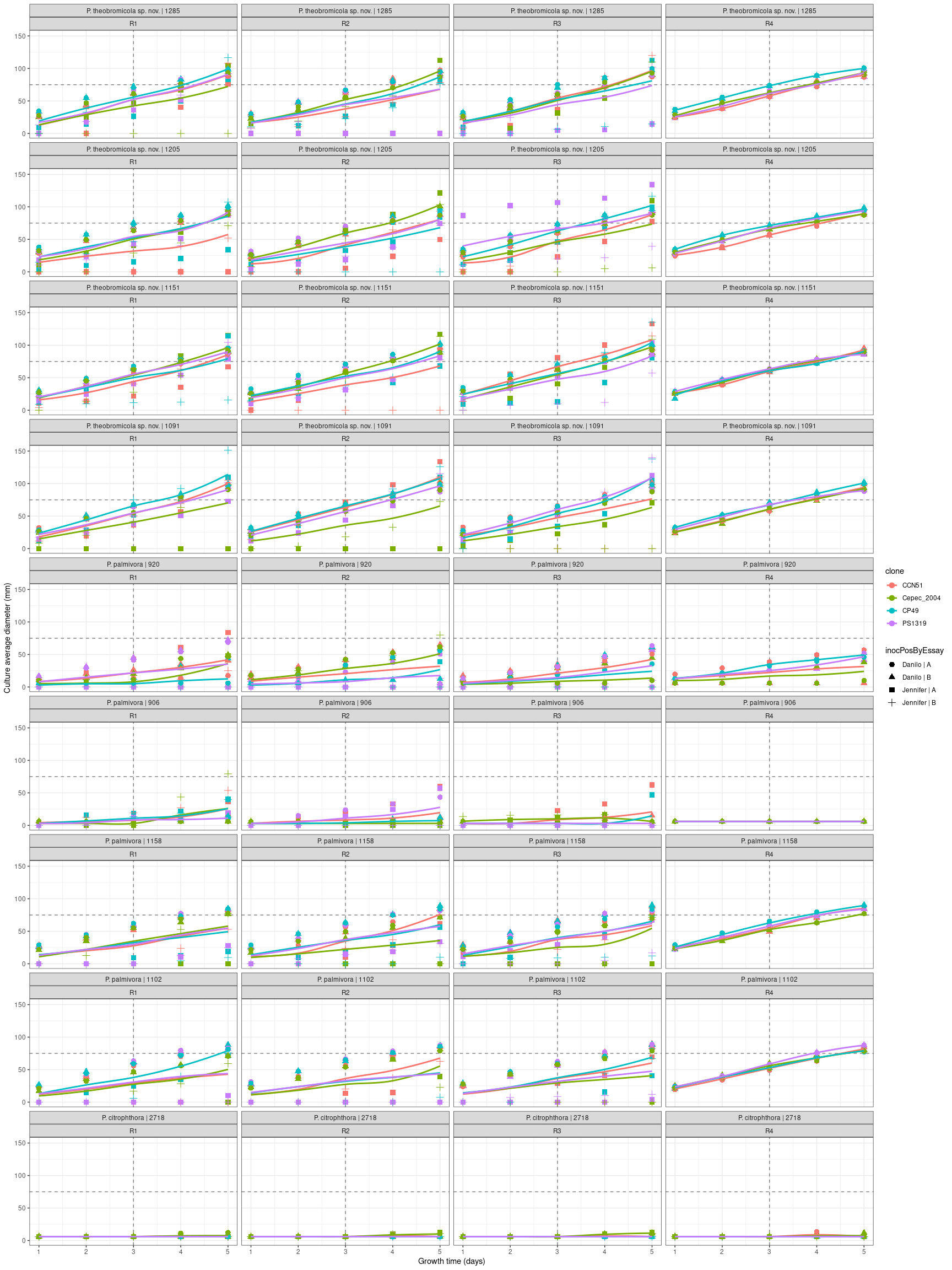

Supplement: Supplementary file 2 [file Data_Sheet_1.ZIP › essay-3/essay-3_files/figure-html/unnamed-chunk-5-1.png]

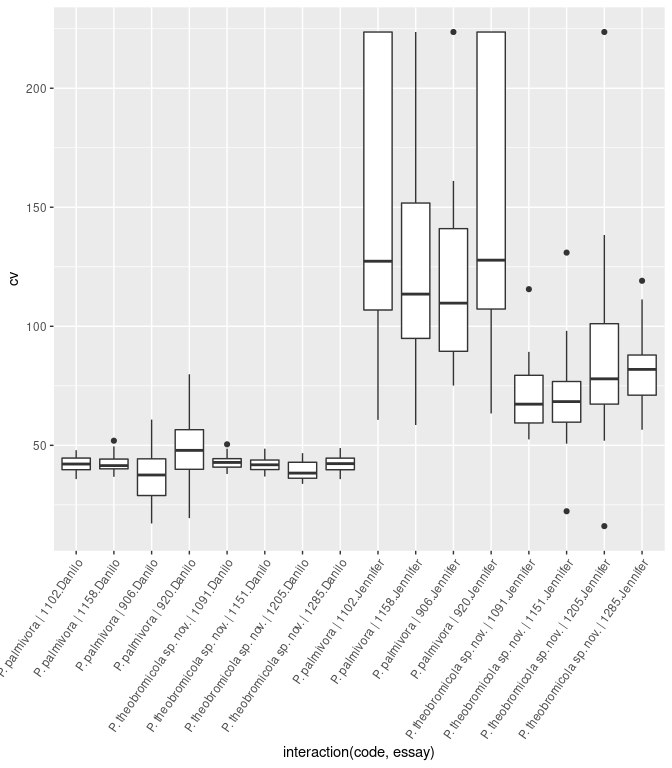

Supplement: Supplementary file 2 [file Data_Sheet_1.ZIP › essay-3/essay-3_files/figure-html/unnamed-chunk-6-1.png]

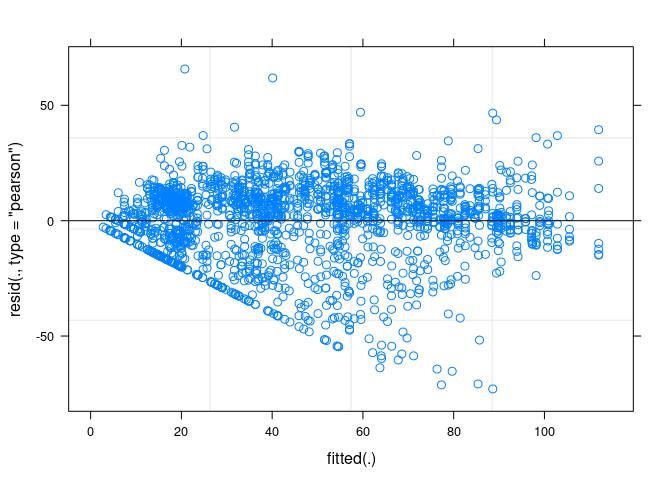

Supplement: Supplementary file 2 [file Data_Sheet_1.ZIP › essay-3/essay-3_files/figure-html/unnamed-chunk-13-1.png]

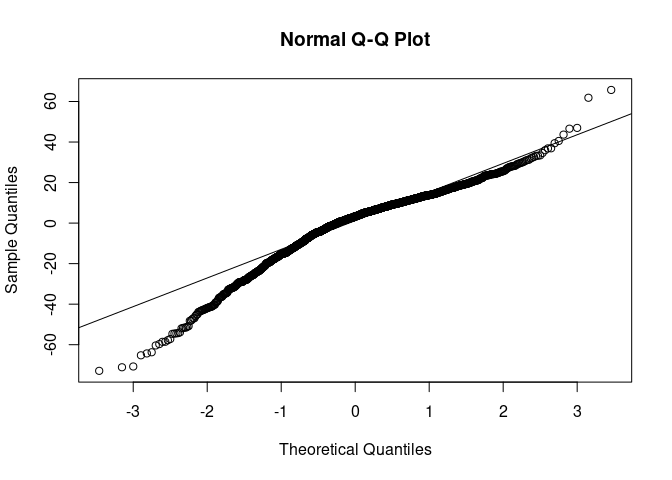

Supplement: Supplementary file 2 [file Data_Sheet_1.ZIP › essay-3/essay-3_files/figure-html/unnamed-chunk-13-2.png]

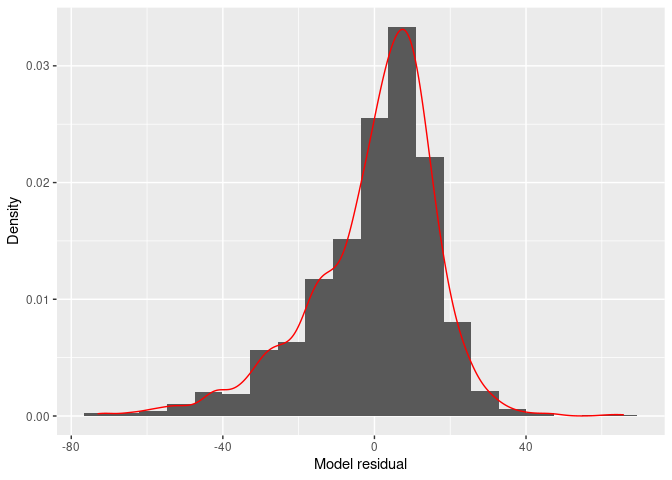

Supplement: Supplementary file 2 [file Data_Sheet_1.ZIP › essay-3/essay-3_files/figure-html/unnamed-chunk-16-1.png]

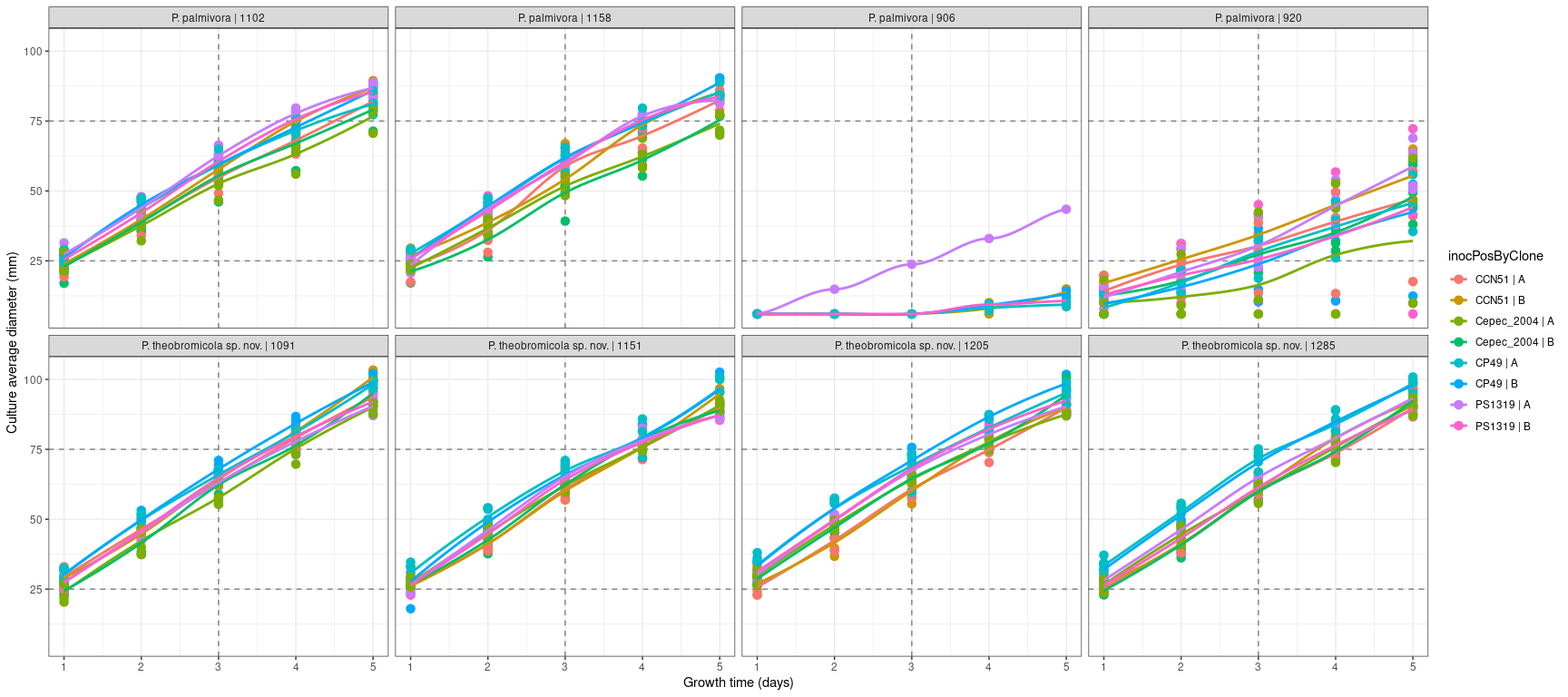

Supplement: Supplementary file 2 [file Data_Sheet_1.ZIP › essay-3/essay-3_files/figure-html/unnamed-chunk-19-1.png]

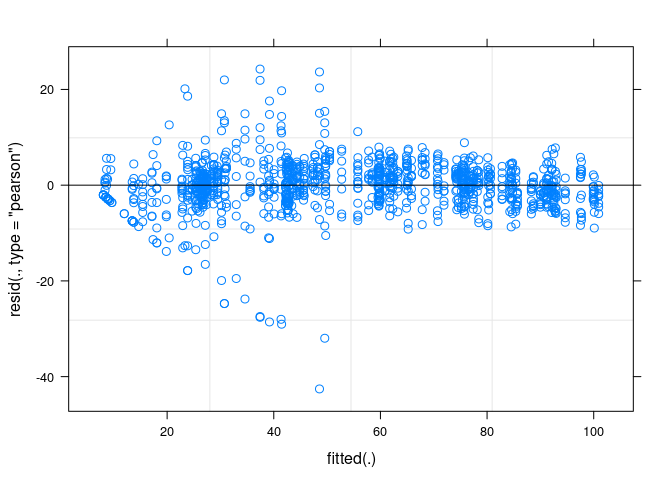

Supplement: Supplementary file 2 [file Data_Sheet_1.ZIP › essay-3/essay-3_files/figure-html/unnamed-chunk-22-1.png]

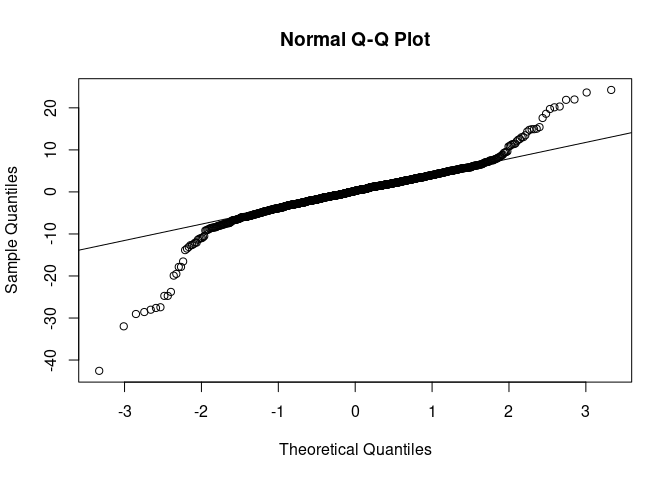

Supplement: Supplementary file 2 [file Data_Sheet_1.ZIP › essay-3/essay-3_files/figure-html/unnamed-chunk-22-2.png]

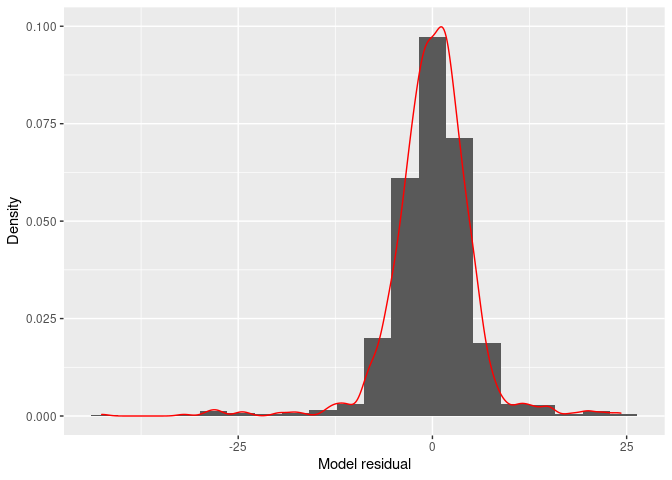

Supplement: Supplementary file 2 [file Data_Sheet_1.ZIP › essay-3/essay-3_files/figure-html/unnamed-chunk-25-1.png]

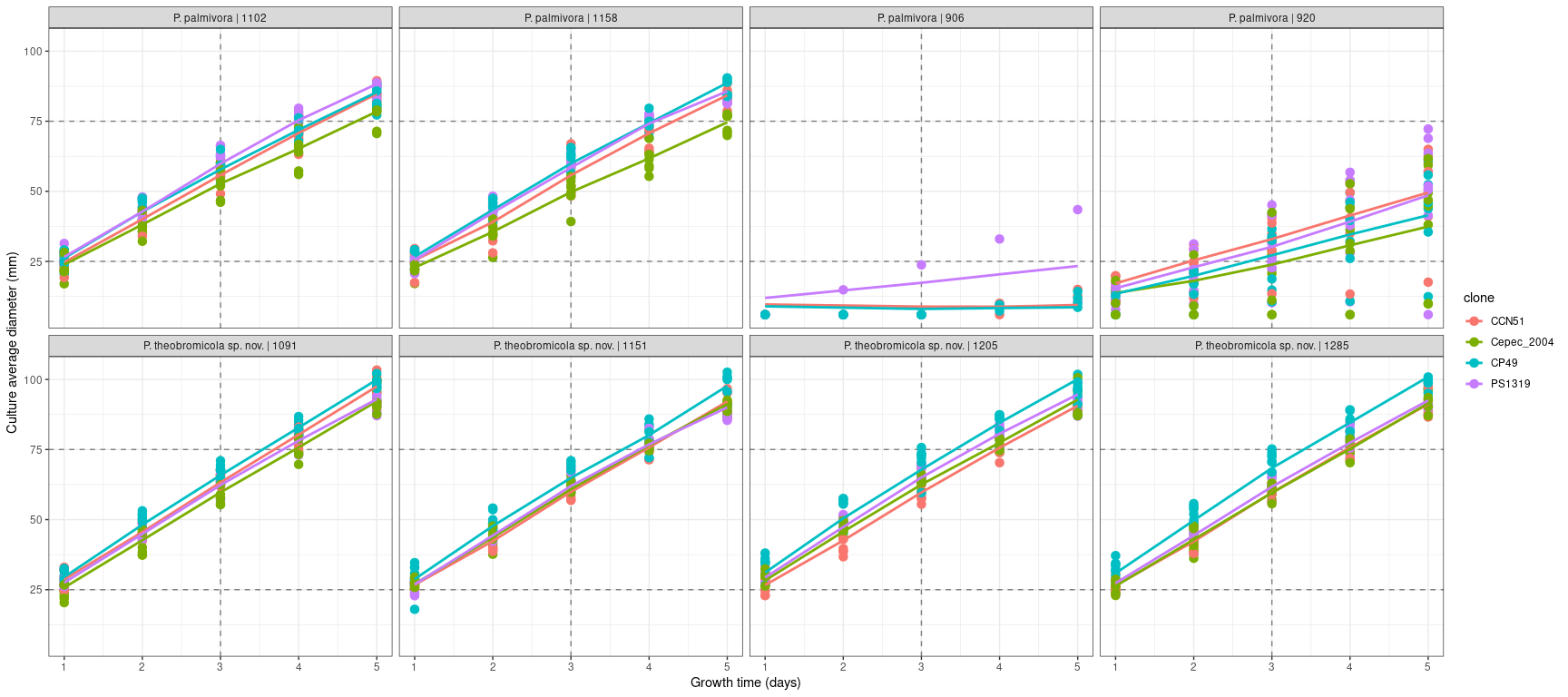

Supplement: Supplementary file 2 [file Data_Sheet_1.ZIP › essay-3/essay-3_files/figure-html/unnamed-chunk-26-1.png]

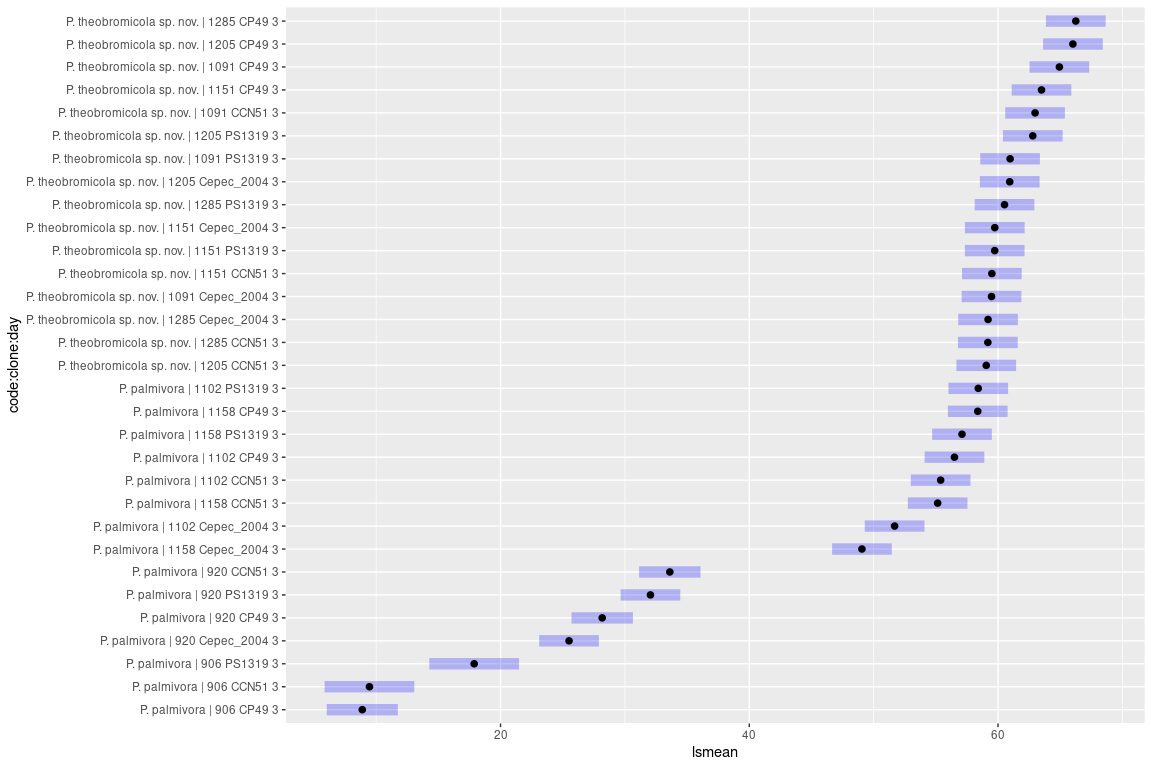

Supplement: Supplementary file 2 [file Data_Sheet_1.ZIP › essay-3/essay-3_files/figure-html/unnamed-chunk-29-1.png]

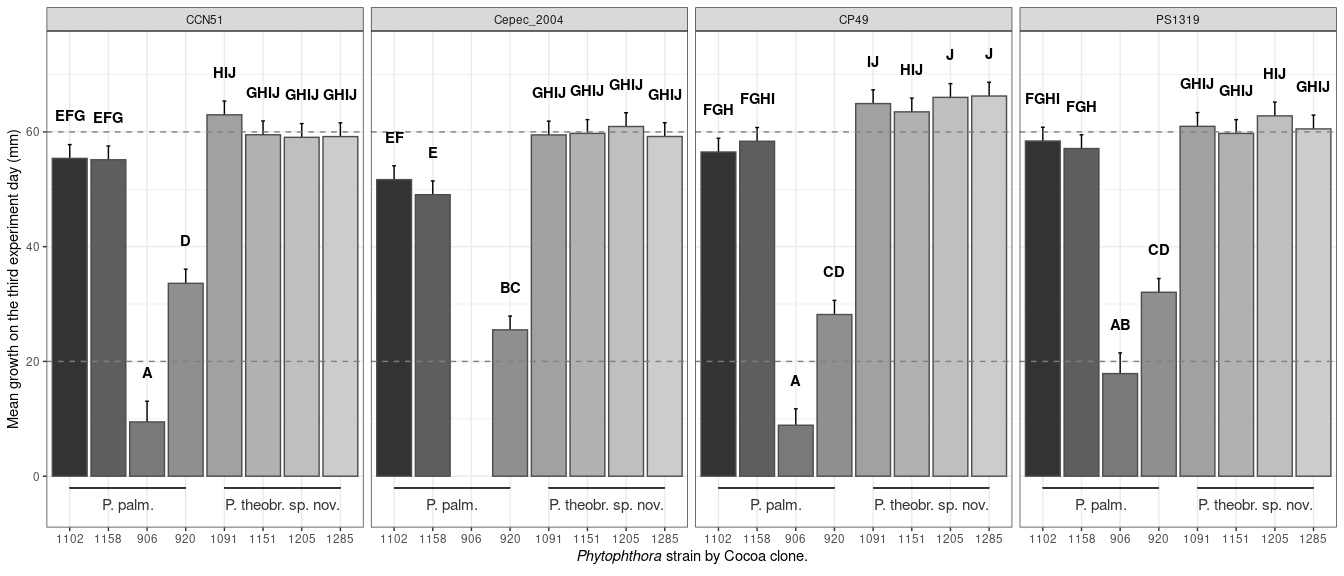

Supplement: Supplementary file 2 [file Data_Sheet_1.ZIP › essay-3/essay-3_files/figure-html/unnamed-chunk-30-1.png]

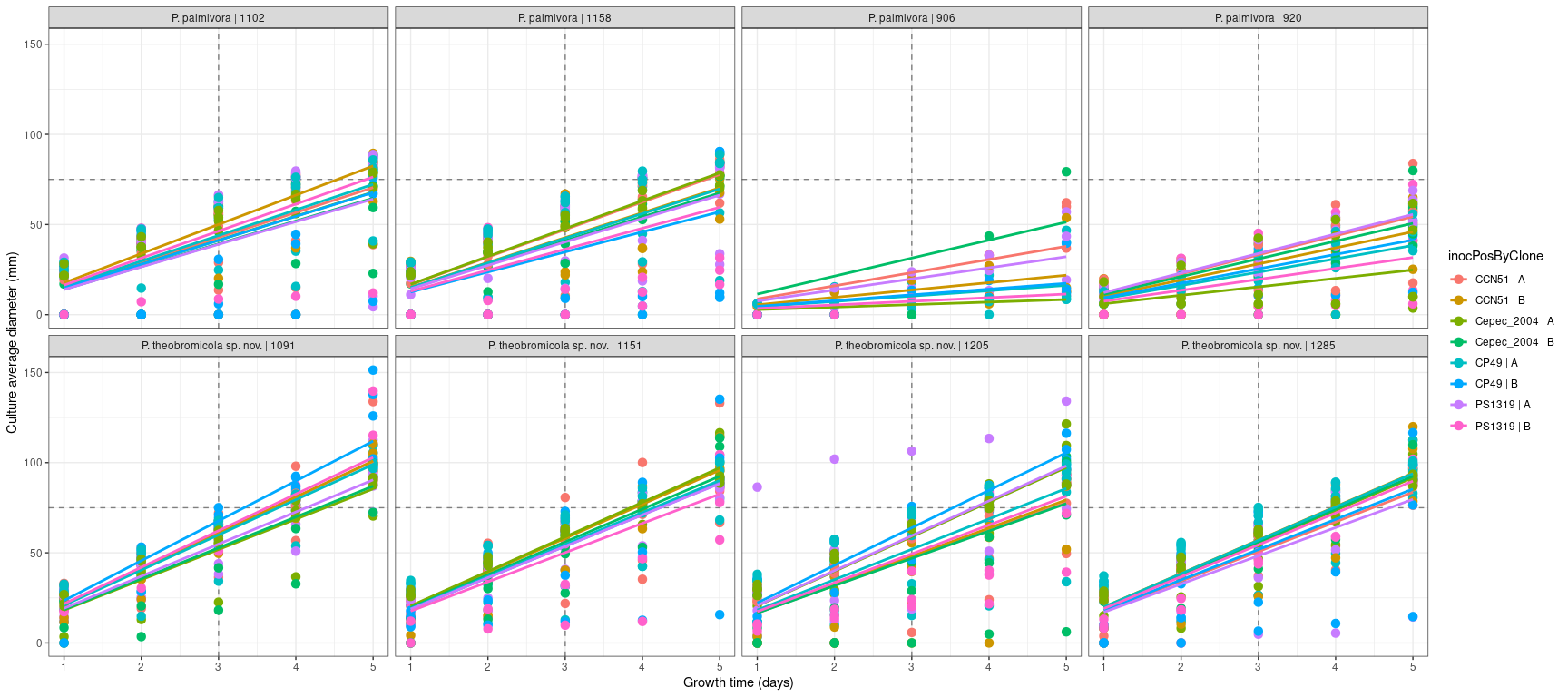

Supplement: Supplementary file 2 [file Data_Sheet_1.ZIP › essay-3/essay-3_files/figure-html/unnamed-chunk-18-1.png]

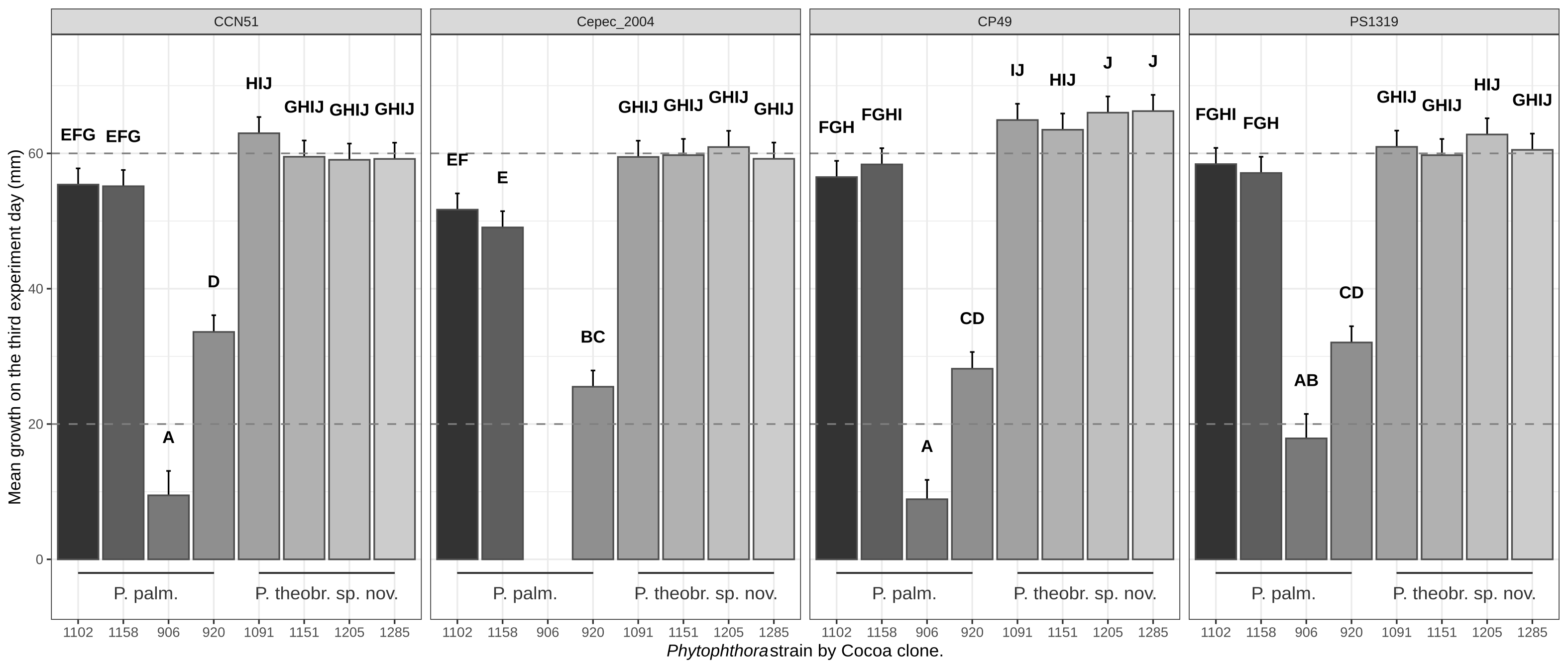

Supplement: Supplementary file 2 [file Data_Sheet_1.ZIP › essay-3/editable-figures/Barplot_predicted_reduced_model.png]
